# Additive prognostic value of functional performance to coronary artery anatomy: the ISCHEMIA trial

**DOI:** 10.1093/ehjci/jeag032

**Published:** 2026-02-04

**Authors:** Sagit Ben Zekry, Georgios Tzimas, Jonathon Leipsic, Samuel Broderick, G B John Mancini, Cameron J Hague, Matthew J Budoff, James K Min, Bernard R Chaitman, Frank W Rockhold, Derek Cyr, Leslee J Shaw, Daniel S Berman, Michael H Picard, Daniel B Mark, Jerome L Fleg, Kian Keong Poh, Ziad A Ali, Gregg W Stone, Sean M O’Brien, Judith S Hochman, David J Maron, Harmony R Reynolds

**Affiliations:** Non-invasive Cardiology Unit, Leviev Heart Center, Sheba Medical Center and Tel Aviv University, Tel Aviv, Israel; Department of Medicine and Radiology, University of British Columbia, Vancouver, St. Paul's Hospital, 1081 Burrard Street Vancouver, BC, V6Z 1Y6, Canada; Service of Cardiology, Lausanne University Hospital and University of Lausanne, Lausanne, Switzerland; Department of Medicine and Radiology, University of British Columbia, Vancouver, St. Paul's Hospital, 1081 Burrard Street Vancouver, BC, V6Z 1Y6, Canada; Duke Clinical Research Institute, Durham, NC, USA; Department of Medicine and Radiology, University of British Columbia, Vancouver, St. Paul's Hospital, 1081 Burrard Street Vancouver, BC, V6Z 1Y6, Canada; Department of Medicine and Radiology, University of British Columbia, Vancouver, St. Paul's Hospital, 1081 Burrard Street Vancouver, BC, V6Z 1Y6, Canada; Department of Medicine and Division of Cardiology, Lundquist Institute, Los Angeles, CA, USA; Cleerly Inc., New York, NY, USA; St. Louis University School of Medicine, Center for Comprehensive Cardiovascular Care, St. Louis, MO, USA; Duke Clinical Research Institute, Durham, NC, USA; Duke Clinical Research Institute, Durham, NC, USA; Division of Cardiology, Icahn School of Medicine at Mount Sinai, New York, NY, USA; Departments of Imaging and Cardiology, Cedars-Sinai Medical Center, Los Angeles, CA, USA; Department of Medicine, Cardiovascular Division, Massachusetts General Hospital, Boston, MA, USA; Department of Medicine, Cardiovascular Division, Harvard Medical School, Cambridge, MA, USA; Duke Clinical Research Institute, Durham, NC, USA; National Heart Lung and Blood Institute, Bethesda, MD, USA; National University Heart Center Singapore, Singapore, Singapore; Yong Loo Lin School of Medicine, National University of Singapore, Singapore, Singapore; Department of Cardiology, St. Francis Hospital, New York, NY, USA; Cardiovascular Research Foundation, NY, USA; Division of Cardiology, Icahn School of Medicine at Mount Sinai, New York, NY, USA; Duke Clinical Research Institute, Durham, NC, USA; Leon H. Charney Division of Cardiology, Department of Medicine, Cardiovascular Clinical Research Center, NYU Grossman School of Medicine, New York, NY, USA; Department of Medicine, Stanford University, Stanford, CA, USA; Leon H. Charney Division of Cardiology, Department of Medicine, Cardiovascular Clinical Research Center, NYU Grossman School of Medicine, New York, NY, USA

**Keywords:** ISCHEMIA trial, Stress testing, Exercise capacity, Coronary artery disease, Prognosis, CCTA

## Abstract

**Aims:**

To assess whether baseline functional performance assessed by exercise treadmill stress testing (EST) has additive value to coronary computed tomography angiography (CCTA) for risk stratification among patients with chronic coronary disease (CCD) and moderate or severe ischaemia.

**Methods and results:**

We performed a subgroup analysis of the ISCHEMIA trial including participants who underwent EST and CCTA. EST data and severity of coronary artery disease (CAD) on CCTA were evaluated by core laboratories, blinded to clinical data and results of the other tests. The primary outcome for this analysis was all-cause death. Secondary outcomes were cardiovascular death, cardiovascular death or myocardial infarction (MI), MI and a composite of cardiovascular death, MI, or hospitalization for heart failure, unstable angina, or resuscitated cardiac arrest. EST and the number of vessels diseased on CCTA were both interpretable in 1864 patients (median age 62 years, IQR 55–68, 83% males). During a median follow-up of 3.1 years, 69 patients died. Higher peak metabolic equivalents (METs) achieved on the qualifying stress test was associated with lower all-cause death (HR 0.86, 95% CI 0.76–0.98; *P* = 0.025). The addition of peak METs to CAD severity improved the predictive ability of the all-cause death and CV death models by 10–20% and 8–13% respectively, depending on the metrics used for CCTA. Adding peak METs to CCTA anatomical models resulted in better prediction of MI by 11–17%, cardiovascular death or MI by 10–14%, and 5-component composite outcome by 12–16%.

**Conclusion:**

Peak METs on EST, a marker of functional performance, added prognostic value to models including CCTA anatomical findings in patients with CCD and moderate or severe ischaemia.

## Graphical Abstract

**Graphical Abstract:**
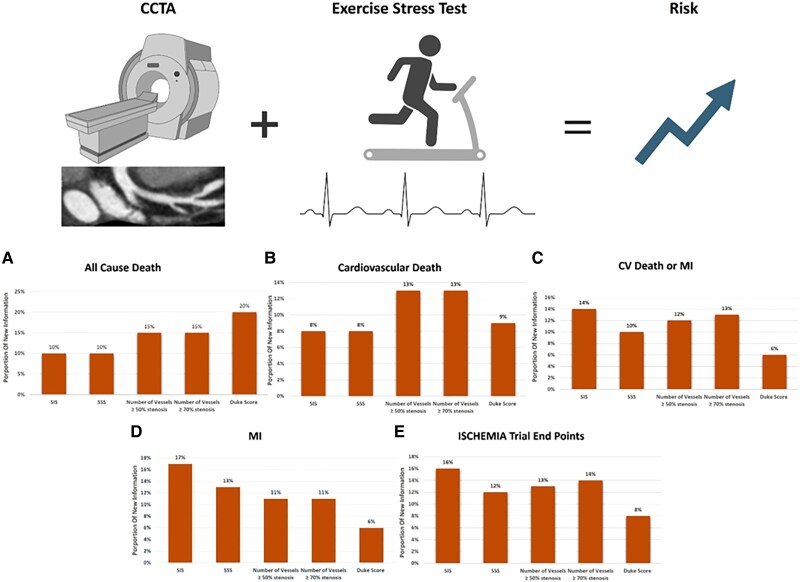
Proportion of new information with the addition of exercise data

**See the editorial comment for this article ‘Beyond ischaemia and anatomy: functional capacity as a stage-specific prognostic driver in advanced coronary atherosclerosis’, by E. Conte**  ***et al*****., https://doi.org/10.1093/ehjci/jeag050.**

## Introduction

Over the last two decades, coronary computed tomography angiography (CCTA) has been established as a first-line imaging modality in the diagnostic work-up of patients with suspected coronary artery disease (CAD), allowing for risk stratification of patients and informing clinical decision-making that improves downstream clinical outcomes.^1^ This led the ACC/AHA chest pain guidelines to recommend CCTA as a class 1, level of evidence A test for evaluation of intermediate- and high-risk patients with stable chest pain and no known CAD.^2^ Previous studies demonstrated that peak aerobic capacity achieved on exercise testing is a strong predictor of cardiac events as well as cardiac and non-cardiac mortality in patients with or without CAD.^3–5^ However, prior retrospective studies found no incremental value of electrocardiographic (ECG) findings after exercise stress to CCTA in predicting outcomes in patients with chest pain^6–8^ or in asymptomatic patients.^9^

To date, there are no prospective data to determine whether functional capacity assessed by exercise treadmill stress test (EST) provides incremental value to CCTA for stratifying long-term risk of mortality and major adverse cardiovascular events. The International Study of Comparative Health Effectiveness with Medical and Invasive Approaches (ISCHEMIA) randomized participants who underwent stress tests, most of whom underwent CCTA, with a median follow-up of 3.2 years for clinical events.^10^ Using the ISCHEMIA trial cohort, we examined whether adding exercise capacity on EST to CCTA anatomical data would improve risk stratification for patients with chronic coronary disease (CCD).

## Methods

The design and primary results of the ISCHEMIA trial have been published.^10–12^ In brief, patients with known or suspected chronic coronary disease were enrolled if moderate or severe ischaemia on an exercise or pharmacological imaging stress test or severe ischaemia on a non-imaging EST based on local interpretation was present^13^ (see Supplementary data online, *Table S1*). Prior to randomization, CCTA was performed in most patients to exclude left main stenosis ≥ 50% and to confirm the presence of obstructive CAD. Patients with estimated glomerular filtration rate (eGFR) less than 60 mL/min/1.73 m^2^ or with known coronary anatomy did not undergo CCTA. Independent core laboratories evaluated all stress tests and CCTA tests, blinded to results of the other modality. Eligible patients were randomized to a routine invasive strategy (invasive coronary angiography plus either percutaneous coronary intervention or coronary artery bypass graft surgery as appropriate, with the goal of complete revascularization, plus guideline-directed medical therapy [GDMT]) or a conservative strategy of GDMT alone without angiography, with invasive testing reserved for failure of GDMT. Key exclusion criteria for randomization were recent acute coronary syndrome, eGFR < 30 mL/min, unprotected left main stenosis of at least 50%, known coronary anatomy that was not suitable for revascularization, or non-obstructive CAD (<50% stenosis) in CCTA or invasive angiography that were performed within 12 months of enrollment, as well as percutaneous coronary intervention or coronary artery bypass graft within 12 months of enrollment, left ventricular ejection fraction less than 35%, non-ischaemic cardiomyopathy, severe valvular disease, New York Heart Association class III or IV heart failure, or unacceptable angina despite maximal medical therapy. All patients provided written informed consent. The protocol was approved by the institutional review board at the New York University Grossman School of Medicine and by the institutional review board or ethics committee at each participating site.

**Figure 1:**
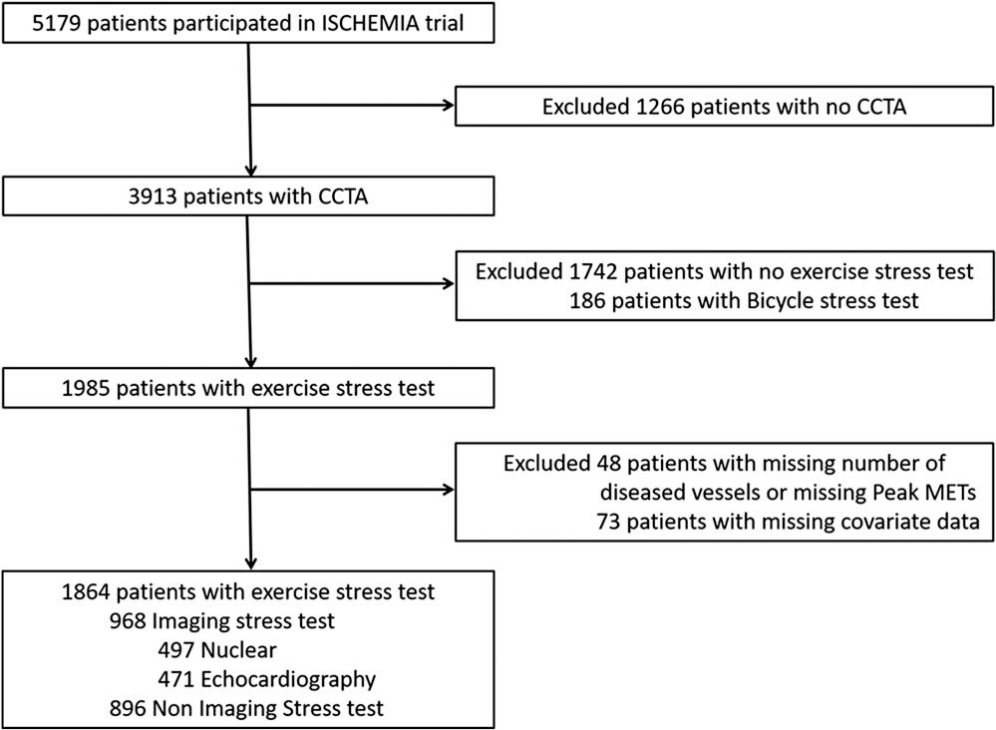
Study flow diagram.

The current analysis comprised patients who had both EST and CCTA, whether EST was with or without imaging. Imaging stress tests included exercise nuclear with single photon emission computed tomography or positron emission tomography, and exercise echocardiography. From the initial ISCHEMIA cohort, we excluded 1266 patients in whom CCTA was not done, 1742 patients without EST, 186 patients with a cycle exercise stress test, 48 patients with missing numbers of diseased vessels or missing Peak METs achieved, and 73 patients with missing covariate data (*Figure 1*). The final analysis cohort thus comprised 1864 patients (896 patients with only EST, 968 patients with exercise imaging stress test).

### Exercise treadmill stress test (EST)

ESTs were performed as part of the clinical evaluation of patients before they were ISCHEMIA trial participants. Protocols (Bruce and Modified Bruce) and symptoms during the EST were documented as well as findings at both baseline and peak exercise heart rate and blood pressure, exercise duration (the duration of exercise time in Modified Bruce protocol was adjusted to Bruce protocol by reducing 3 min), peak estimated metabolic equivalents of task (METs),^14^ maximal age-predicted heart rate (defined as 220 minus age at test), target heart rate defined as 85% of maximal age predicted heart rate, ECG changes of ST depression ≥ 1 mm, ventricular arrhythmia (defined as ≥ 7 ventricular premature beats/min, couplets, ventricular tachycardia or ventricular fibrillation). Rate pressure product was defined as peak systolic heart rate * peak systolic blood pressure. Due to a high frequency of missing values, we did not include the use of beta blockers at the time of stress testing, or the maximum severity of ST depression, in the analysis.

### Coronary CT angiography

Segmental interpretation of the CCTA was carried out according to Society of Cardiovascular Computed Tomography guidelines.^15^ Number of vessels involved, segment involvement score (number of affected coronary segments, SIS) and segment stenosis score (calculated by summing grades for all coronary segments as: 0, no plaque; 1, < 50% stenosis; 2, 50–69% stenosis; and 3, ≥70% stenosis, SSS) were collected. Obstructive CAD was defined as stenosis of ≥ 50%. A repeated analysis was done with a definition of obstructive disease ≥ 70% stenosis and modified Duke Prognostic Index.^16^

### Outcomes

The primary outcome for this analysis was all-cause death. Secondary outcomes for this analysis included cardiovascular (CV) death, CV death or myocardial infarction (MI), MI and the ISCHEMIA trial primary endpoint, which was a composite of cardiovascular death, MI, or hospitalization for heart failure, unstable angina, or resuscitated cardiac arrest.

### Statistical analysis

Baseline characteristics are presented as medians (25th, 75th percentiles) for continuous variables and as counts (percentages) for categorical variables. The relationships between the risk of an outcome and CCTA findings, as well as EST findings, were assessed using Cox proportional hazards models. Relationships were assessed without adjustment for other covariates in univariate models. Relationships were also assessed, adjusting for age, sex, eGFR, ejection fraction, and diabetes in multivariate models. These adjusted models also included the randomized treatment strategy as a stratum effect. We studied peak METs achieved^14^ as the primary EST measure of exercise performed, with total exercise time on a standard or modified Bruce protocol as a secondary analysis.

To assess the incremental value of aerobic exercise capacity beyond the combination of CCTA data and baseline characteristics, we determined the proportion of new prognostic information contributed by exercise capacity data in the model that included baseline + CCTA data + EST data.^17,18^ The amount of prognostic information contained in a model can be determined by calculating the variation of the predicted probabilities for the endpoint in the model. Higher variances indicate an increased ability of the model to assess the risk of the model endpoint and, thus, indicate a greater amount of prognostic information contained in the model. The total amount of prognostic information in the model that includes baseline + CCTA data + EST data can be split into (i) the amount of information contributed by baseline + CCTA data and (ii) the amount of information contributed by EST data. The proportion of information contributed by baseline + CCTA data (Prop_ccta_) can be calculated by taking the variance of the predicted probabilities of an endpoint for the model including baseline + CCTA data (Var_ccta_) divided by the variance of the predicted probabilities of an endpoint for the model including baseline + CCTA data + EST data (Var_ccta + est_).

Thus,


$$\mathrm{Pro}p_{\mathrm{ccta}}=\frac{\mathrm{Va}r_{\mathrm{ccta}}}{\mathrm{Va}r_{\mathrm{ccta}\;+\;\mathrm{est}}}$$

Once Prop_ccta_ is known, the proportion of information contributed by EST data (Prop_ccta + est_) can be determined by subtracting Prop_ccta_ from 1.

Thus,


$$\mathrm{Pro}p_{\mathrm{ccta}\;+\;\mathrm{est}}=1-\mathrm{Pro}p_{\mathrm{ccta}}=1-\frac{\mathrm{Va}r_{\mathrm{ccta}}}{\mathrm{Va}r_{\mathrm{ccta}\;+\;\mathrm{est}}}$$

Prop_ccta+est_, or the proportion of the total predictive information in the baseline + CCTA + EST model that was added by including EST data, will correspond to the incremental prognostic value of EST data beyond the combination of baseline and CCTA data.

We compared the incremental value of exercise duration and peak METs against four CCTA variables: (i) segment involvement score (SIS) on CCTA; (ii) segment stenosis score (SSS) on CCTA; (iii) number of diseased vessels as defined based on the 50% stenosis threshold by CCTA; and (iv) number of diseased vessels as defined based on a 70% stenosis threshold by CCTA. The baseline covariates that were included in the model, including baseline + CCTA data and the model including baseline + CCTA data + EST data, were age at randomization, sex, geographic region of enrollment, diabetes, hypertension, current smoker, prior MI, prior revascularization, new or increasing angina, eGFR, ejection fraction, and body mass index (BMI). A two-tailed *P*-value < 0.05 was considered to be statistically significant.

## Results

### Study population

Among the 1864 patients in the current analysis, 69 participants died during a median follow-up of 3.1 years (IQR: 2.1–4.1 years). *Table 1* summarizes the baseline characteristics of the overall cohort and comparison of participants who did and did not die during follow-up. Compared with patients who survived, those who died were older and more likely of North American origin. Hypertension and atrial fibrillation were more common among patients who died, although HbA1c was lower compared with survivors. Higher eGFR and more frequent aspirin use were noted among patients who survived. Three-vessel disease with ≥ 70% stenosis was more common among patients who died than in survivors (26.1% vs. 13%, *P* = 0.02). Patients who diedalso more commonly had proximal LAD disease than survivors (64.7% vs. 49.8%, *P* = 0.016). Both SSS and SIS were also considerably higher for participants who died compared to survivors. The use of imaging during the qualifying EST was not different among groups, nor was ischaemia severity. However, mean exercise times and peak METs were lower among participants who died vs. survived (5.5 min vs. 6.2 min, *P* = 0.011 and 6.8 METs vs. 7.4 METs, *P* = 0.014, respectively).

**Table 1:** Baseline characteristics

Characteristic	Overall (*N* = 1864)	Died (*N* = 69)	Survived (*N* = 1795)	*P*-value
**Demographics**				
Age at randomization (years)				<0.001
Median (interquartile)	62 (55, 68)	68 (61, 73)	62 (55, 68)	
Sex				0.48
Male	1545/1864 (82.9%)	55/69 (79.7%)	1490/1795 (83%)	
Female	319/1864 (17.1%)	14/69 (20.3%)	305/1795 (17%)	
Race				0.27
American Indian or Alaskan Native	8/1848 (0.4%)	0/69 (0%)	8/1779 (0.4%)	
Asian	886/1848 (47.9%)	25/69 (36.2%)	861/1779 (48.4%)	
Native Hawaiian or Other Pacific Islander	2/1848 (0.1%)	0/69 (0%)	2/1779 (0.1%)	
Black or African American	59/1848 (3.2%)	3/69 (4.3%)	56/1779 (3.1%)	
White	886/1848 (47.9%)	40/69 (58%)	846/1779 (47.6%)	
Multiple races reported	7/1848 (0.4%)	1/69 (1.4%)	6/1779 (0.3%)	
Region				0.004
Asia	827/1864 (44.4%)	25/69 (36.2%)	802/1795 (44.7%)	
Europe	477/1864 (25.6%)	12/69 (17.4%)	465/1795 (25.9%)	
Latin America	136/1864 (7.3%)	12/69 (17.4%)	124/1795 (6.9%)	
North America	402/1864 (21.6%)	18/69 (26.1%)	384/1795 (21.4%)	
Other	22/1864 (1.2%)	2/69 (2.9%)	20/1795 (1.1%)	
Height (cm)				0.97
Median (interquartile)	168.0 (161, 175)	168.0 (160, 175)	168.0 (161, 175)	
Weight (kg)				0.12
Median (interquartile)	75.0 (65, 86.6)	70.3 (63, 84)	75.0 (65, 87)	
BMI (kg/m^2^)				0.068
Median (Interquartile)	26.8 (24.2, 29.8)	26.2 (23.7, 29)	26.8 (24.2, 29.8)	
Body surface area (m^2^)				
Median (interquartile)	1.8 (1.7, 2)	1.8 (1.7, 2)	1.8 (1.7, 2)	0.2
**Observed follow-up time (years)**				<0.001
Median (Interquartile)	3.1 (2.1, 4.1)	1.7 (0.7, 3.3)	3.1 (2.2, 4.2)	
**Clinical history**				
Hypertension	1155/1858 (62.2%)	51/69 (73.9%)	1104/1789 (61.7%)	0.04
Diabetes	729/1864 (39.1%)	34/69 (49.3%)	695/1795 (38.7%)	0.078
Diabetes treatment				0.6
Insulin treated	107/711 (15%)	7/33 (21.2%)	100/678 (14.7%)	
Non-insulin diabetes medication	492/711 (69.2%)	21/33 (63.6%)	471/678 (69.5%)	
None/Diet controlled	112/711 (15.8%)	5/33 (15.2%)	107/678 (15.8%)	
Cigarette smoking				0.98
Never smoked	899/1864 (48.2%)	33/69 (47.8%)	866/1795 (48.2%)	
Former smoker	765/1864 (41%)	29/69 (42%)	736/1795 (41%)	
Current smoker	200/1864 (10.7%)	7/69 (10.1%)	193/1795 (10.8%)	
Family history of premature coronary heart disease	431/1647 (26.2%)	13/58 (22.4%)	418/1589 (26.3%)	0.51
Atrial fibrillation/Atrial flutter	31/1863 (1.7%)	5/69 (7.2%)	26/1794 (1.4%)	0.005
Peripheral vascular disease	32/1859 (1.7%)	3/69 (4.3%)	29/1790 (1.6%)	0.11
Cerebrovascular disease	89/1858 (4.8%)	3/69 (4.3%)	86/1789 (4.8%)	1
Prior MI	227/1856 (12.2%)	8/69 (11.6%)	219/1787 (12.3%)	0.87
Prior PCI	266/1862 (14.3%)	6/69 (8.7%)	260/1793 (14.5%)	0.18
Prior CABG	23/1864 (1.2%)	2/69 (2.9%)	21/1795 (1.2%)	0.21
New or worsening angina	559/1864 (30%)	17/69 (24.6%)	542/1795 (30.2%)	0.32
Ejection rraction (%)				0.79
Median (interquartile)	60.0 (56, 65)	60.0 (56.5, 65)	60.0 (56, 65)	
**Medications**				
Aspirin	1714/1862 (92.1%)	57/69 (82.6%)	1657/1793 (92.4%)	0.003
Thienopyridines	574/1862 (30.8%)	19/69 (27.5%)	555/1793 (31%)	0.55
Beta blockers	1521/1862 (81.7%)	57/69 (82.6%)	1464/1793 (81.7%)	0.84
Calcium channel blockers	483/1862 (25.9%)	19/69 (27.5%)	464/1793 (25.9%)	0.76
Long-acting nitrates	745/1862 (40%)	27/69 (39.1%)	718/1793 (40%)	0.88
ACE inhibitor or ARB	1061/1862 (57%)	37/69 (53.6%)	1024/1793 (57.1%)	0.566
Statins	1792/1862 (96.2%)	65/69 (94.2%)	1727/1793 (96.3%)	0.33
High-intensity statins	841/1862 (45.2%)	30/69 (43.5%)	811/1793 (45.2%)	0.77
**Laboratory data**				
eGFR from enrollment (mL/min)				0.016
Median (interquartile)	87 (74, 103)	79.0 (67, 98)	87 (74, 103)	
Creatinine at enrollment (mg/dL)				0.09
Median (interquartile)	0.9 (0.8, 1)	0.9 (0.8, 1.1)	0.9 (0.8, 1)	
Hemoglobin (g/dL)				0.63
Median (interquartile)	14.1 (13, 15)	13.9 (13, 14.8)	14.1 (13, 15)	
Platelets (10^9^/L)				0.13
Median (interquartile)	220 (181, 261)	228.0 (200, 273)	219 (180, 260)	
Total cholesterol (mg/dL)				0.62
Median (interquartile)	152.4 (127, 184)	151.2 (123.9, 173.5)	152.5 (127.6, 184)	
Trigylcerides (mg/dL)				0.18
Median (interquartile)	121.2 (91, 172)	114.2 (93, 144)	122 (91, 173)	
HDL cholesterol (mg/dL)				0.61
Median (interquartile)	42 (35, 49)	42.5 (37, 50.3)	42 (35, 49)	
LDL cholesterol (mg/dL)				0.97
Median (interquartile)	83 (64, 109.5)	85 (66, 101)	83 (63, 110)	
HbA1c for diabetics (%)				0.03
Median (interquartile)	7.3 (6.6, 8.5)	6.7 (5.9, 7.9)	7.3 (6.6, 8.6)	
**CCTA findings (≥50% stenosis)**				
Any obstructive disease	1827/1828 (99.9%)	69/69 (100.0%)	1758/1759 (99.9%)	1
Multi-vessel disease	1275/1630 (78.2%)	57/66 (86.4%)	1218/1564 (77.9%)	0.1
Number of diseased vessels				0.05
0	1/1864 (0.1%)	0/69 (0%)	1/1795 (0.1%)	
1	348/1864 (18.7%)	9/69 (13%)	339/1795 (18.9%)	
2	449/1864 (24.1%)	14/69 (20.3%)	435/1795 (24.2%)	
3	657/1864 (35.2%)	36/69 (52.2%)	621/1795 (34.6%)	
Non-evaluable	409/1864 (21.9%)	10/69 (14.5%)	399/1795 (22.2%)	
**CCTA findings (≥70% stenosis)**				
Any obstructive disease	1604/1711 (93.7%)	60/64 (93.8%)	1544/1647 (93.7%)	1
Multi-vessel disease	763/1472 (51.8%)	38/61 (62.3%)	725/1411 (51.4%)	0.095
Number of diseased vessels				0.02
0	107/1864 (5.7%)	4/69 (5.8%)	103/1795 (5.7%)	
1	530/1864 (28.4%)	14/69 (20.3%)	516/1795 (28.7%)	
2	371/1864 (19.9%)	10/69 (14.5%)	361/1795 (20.1%)	
3	251/1864 (13.5%)	18/69 (26.1%)	233/1795 (13%)	
Non-evaluable	605/1864 (32.5%)	23/69 (33.3%)	582/1795 (32.4%)	
**Modified Duke prognostic index (not mentioned in text—would omit here)**				0.042
No vessel (LAD, LCX, RCA) with at least moderate (≥50%) stenosis	1/1255 (0.1%)	0/46 (0%)	1/1209 (0.1%)	
1 vessel with at least moderate (≥50%) stenosis	67/1255 (5.3%)	2/46 (4.3%)	65/1209 (5.4%)	
2 vessels with at least moderate (≥50%) stenosis or 1 severe (≥70%) stenosis	363/1255 (28.9%)	10/46 (21.7%)	353/1209 (29.2%)	
3 vessels with at least moderate (≥50%) stenosis or 2 severe (≥70%) stenosis or proximal LAD with severe (≥ 70%) stenosis	448/1255 (35.7%)	11/46 (23.9%)	437/1209 (36.1%)	
3 vessels with severe (≥70%) stenosis or 2 severe (≥70%) stenosis including proximal LAD	361/1255 (28.8%)	21/46 (45.7%)	340/1209 (28.1%)	
Left main ≥ 50% stenosis	15/1255 (1.2%)	2/46 (4.3%)	13/1209 (1.1%)	
Segment stenosis score				
Median (interquartile)	24 (18, 30)	28.0 (22, 34)	24 (18, 30)	0.001
Segment involvement score				
Median (interquartile)	12 (9, 13)	13.0 (11, 14)	12.0 (9, 13)	0.002
**Qualifying stress test core lab interpretation**				
Exercise stress imaging overall	968/1864 (51.9%)	39/69 (56.5%)	929/1795 (51.8%)	0.437
*Severity*				0.44
Severe	530/968 (54.8%)	26/39 (66.7%)	504/929 (54.3%)	
Moderate	353/968 (36.5%)	10/39 (25.6%)	343/929 (36.9%)	
Mild	60/968 (6.2%)	2/39 (5.1%)	58/929 (6.2%)	
None	25/968 (2.6%)	1/39 (2.6%)	24/929 (2.6%)	
Non-imaging EST	896/1864 (48.1%)	30/69 (43.5%)	866/1795 (48.2%)	0.59
*Severity*				0.73
				
Severe	807/896 (90.1%)	27/30 (90%)	780/866 (90.1%)	
Moderate	60/896 (6.7%)	2/30 (6.7%)	58/866 (6.7%)	
Mild	16/896 (1.8%)	1/30 (3.3%)	15/866 (1.7%)	
None	13/896 (1.5%)	0/30 (0%)	13/866 (1.5%)	
Ischemia severity by exercise imaging modality				
***Nuclear***	497/1864 (26.7%)	22/69 (31.9%)	475/1795 (26.5%)	0.59
Severity				0.77
Severe	221/497 (44.5%)	11/22 (50.0%)	210/475 (44.2%)	
Moderate	228/497 (45.9%)	9/22 (40.9%)	219/475 (46.1%)	
Mild	34/497 (6.8%)	1/22 (4.5%)	33/475 (6.9%)	
None	14/497 (2.8%)	1/22 (4.5%)	13/475 (2.7%)	
***Echocardiogram***	471/1864 (25.3%)	17/69 (24.6%)	454/1795 (25.3%)	0.59
Severity				0.17
Severe	309/471 (65.6%)	15/17 (88.2%)	294/454 (64.8%)	
Moderate	125/471 (26.5%)	1/17 (5.9%)	124/454 (27.3%)	
Mild	26/471 (5.5%)	1/17 (5.9%)	25/454 (5.5%)	
None	11/471 (2.3%)	0/17 (0.0%)	11/454 (2.4%)	
**Exercise stress test variables**				
Protocol				0.3
Standard Bruce Treadmill Protocol	1752/1864 (94%)	63/69 (91.3%)	1689/1795 (94.1%)	
Modified Bruce Treadmill Protocol	112/1864 (6%)	6/69 (8.7%)	106/1795 (5.9%)	
Exercise duration (min)				
Median (interquartile)	6.2 (4.6, 7.8)	5.5 (4, 7)	6.2 (4.6, 7.9)	0.011
Peak METs				
Median (interquartile)	7.4 (6, 8.9)	6.8 (5.6, 8.2)	7.4 (6.1, 8.9)	0.014
Heart rate (bpm)				
Rest				
Median (interquartile)	75 (66, 86)	76 (67, 87)	75 (66, 86)	0.7
Exercise				
Median (interquartile)	139 (126, 151)	139 (123, 144)	139 (126, 151)	0.19
Maximum age-predicted heart rate				
Median (interquartile)	158 (152, 164)	152 (147, 159)	158 (152, 165)	<0.001
Systolic blood pressure (mmHg)				
Rest				
Median (interquartile)	135 (123, 148)	140 (130, 151)	134 (122, 148)	0.024
Exercise				
Median (interquartile)	162 (150, 180)	170 (140, 190)	161 (150, 180)	0.81
Diastolic blood pressure (mmHg)				
Rest				
Median (interquartile)	80 (76, 90)	80 (75, 90)	80 (76, 90)	0.79
Exercise				
Median (interquartile)	80 (79, 90)	80 (71, 90)	80 (79, 90)	0.84
Symptoms during exercise	1348/1823 (73.9%)	49/69 (71.0%)	1299/1754 (74.1%)	0.57
Type of symptom				0.2
Limiting exertional chest pain	461/1336 (34.5%)	13/49 (26.5%)	448/1287 (34.8%)	
Non-limiting exertional chest pain	377/1336 (28.2%)	13/49 (26.5%)	364/1287 (28.3%)	
Dyspnea	333/1336 (24.9%)	19/49 (38.8%)	314/1287 (24.4%)	
Claudication	18/1336 (1.3%)	0/49 (0%)	18/1287 (1.4%)	
Other	147/1336 (11%)	4/49 (8.2%)	143/1287 (11.1%)	
Target heart rate achieved	1026/1656 (62%)	38/57 (66.7%)	988/1599 (61.8%)	0.46
**ECG changes**				
ST depression ≥1.0 mm	1237/1254 (98.6%)	43/43 (100%)	1194/1211 (98.6%)	1
Frequency ventricular arrhythmia	55/1143 (4.8%)	2/37 (5.4%)	53/1106 (4.8%)	0.7

Abbreviations: 109/L, billions per liter; ACE, angiotensin-converting enzyme; ARB, angiotensin receptor blocker; bpm, beats per minute; CABG, coronary artery bypass grafting; CCTA, coronary computed tomography angiography; cm, centimeters; ECG, electrocardiogram; eGFR, estimated glomerular filtration rate; EST, exercise tolerance test; kg, kilograms; kg/m^2^, kilograms/meters squared; LAD, left anterior descending; LCX, left circumflex; m^2^, meters squared; METs, metabolic equivalence of task; mg/dL, milligrams per deciliter; MI, myocardial infarction; min, minute; mmHg, millimeters of mercury; PCI, percutaneous coronary intervention; RCA, right coronary artery; ST, ST segment.

### Associations between exercise treadmill stress test findings and outcomes

#### All-cause mortality

On adjusted analysis, higher METs achieved on the pre-randomization stress test was associated with a reduced rate of all-cause death (HR of 0.86 per MET achieved, 95% CI 0.76–0.98; *P* = 0.025). Exercise duration was inversely correlated with the primary outcome event rate (HR 0.90 per minute of exercise, 95% CI 0.82–0.98; *P* = 0.032) (*Table 2*). However, heart rate achieved, symptoms or frequent ventricular arrhythmia were not associated with mortality.

**Table 2:** Univariable and adjusted cox models for prediction of all-cause death

Characteristic	Univariable analysis		Adjusted analysis^a^	
	HR (95% CI)	*P*-value	HR (95% CI)	*P*-value
**CCTA findings (≥50% stenosis)**				
Number of diseased vessels (compared to 1-vessel disease)		0.02		0.057
2 vessel disease	1.20 (0.52, 2.78)		1.08 (0.46, 2.54)	
3 vessel disease	2.26 (1.09, 4.69)		2.06 (0.97, 4.37)	
Non-evaluable	0.97 (0.39, 2.38)		1.00 (0.40, 2.49)	
Segment stenosis score (per 5-unit increase)	1.28 (1.11, 1.48)	<0.001	1.20 (1.03, 1.40)	0.019
Segment involvement score (per 1-unit increase)	1.19 (1.07, 1.32)	0.001	1.15 (1.03, 1.28)	0.014
Duke prognostic score (compared to 2V ≥ 50% or 1V ≥ 70%)		0.039		0.11
1V ≥ 50%	1.10 (0.24, 5.01)		0.7 (0.14, 3.49)	
3V ≥ 50% or 2V ≥ 70% or ≥70% proximal LAD	0.93 (0.40, 2.2)		0.78 (0.33, 1.86)	
3V ≥ 70% or 2V ≥ 70% including proximal LAD or LM ≥ 50%	2.28 (1.09, 4.8)		1.80 (0.83, 3.92)	
**Exercise test findings**				
Ischemia severity (compared to mild or no ischaemia)		0.43		0.19
Moderate	0.67 (0.21, 2.07)		0.59 (0.19, 1.89)	
Severe	1.01 (0.37, 2.79)		1.11 (0.39, 3.17)	
Heart rate (per 10 bpm increase)				
Rest	1.01 (0.85, 1.19)	0.92	1.08 (0.90, 1.31)	0.4
Exercise	0.90 (0.78, 1.03)	0.13	0.95 (0.82, 1.11)	0.52
Target heart rate achieved	1.20 (0.69, 2.08)	0.52	1.00 (0.57, 1.78)	0.99
Systolic blood pressure (per 10 mmHg increase)				
Rest	1.19 (1.03, 1.37)	0.02	1.15 (0.99, 1.34)	0.07
Exercise	0.95 (0.86, 1.05)	0.027	0.97 (0.87, 1.09)	0.06
Diastolic blood pressure (per 10 mmHg increase)				
Rest	0.95 (0.73, 1.23)	0.68	0.99 (0.75, 1.31)	0.95
Exercise	0.97 (0.82, 1.14)	0.007	1.23 (0.98, 1.55)	0.004
Rate pressure product (per 500-unit increase)	0.99 (0.96, 1.02)	0.57	1.01 (0.98, 1.04)	0.68
METs	0.83 (0.74, 0.94)	0.003	0.86 (0.76, 0.98)	0.025
Exercise duration (per 1-min increase)	0.90 (0.83, 0.98)	0.004	0.9 (0.82, 0.98)	0.032
Symptoms during exercise	0.93 (0.55, 1.57)	0.79	1.01 (0.58, 1.76)	0.97
**ECG changes**				
ST depression ≥ 1.0 mm	N/A	N/A	N/A	N/A
Frequent ventricular arrhythmia	0.82 (0.19, 3.46)	0.79	0.99 (0.21, 4.79)	0.99

Abbreviations: 1V, 1-vessel; 2V, 2-vessel; bpm, beats per minute; CCTA, coronary computed tomography angiography; ECG, electrocardiogram; METs, metabolic equivalence of task; mmHg, millimeters of mercury; ST, ST segment.

^a^Adjusted model included age, sex, eGFR, ejection fraction, and diabetes. Randomized treatment strategy was included as a stratum effect.

#### Secondary endpoints

As shown in Supplementary data online, *Tables S2*–*S5*, higher peak METs achieved was associated with lower risk of cardiovascular death (HR 0.85 per MET, 95% CI 0.72–1.00; *P* = 0.046), MI (HR 0.89, 95% CI 0.82–0.97; *P* = 0.009), cardiovascular death or MI (HR 0.90, 95% CI 0.83–0.97; *P* = 0.007) and the ISCHEMIA trial primary composite end point (HR 0.90, 95% CI 0.83–0.97; *P* = 0.004). Exercise duration was correlated with better outcome for the end point of MI (HR 0.94, 95% CI 0.87–1.00; *P* = 0.049) and the ISCHEMIA trial composite end point (HR 0.94, 95% CI 0.88–0.99; *P* = 0.03). Moreover, higher resting systolic BP was associated with a greater risk of all trial secondary end points: MI (HR 1.11, 95% CI 1–1.23; *P* = 0.045), cardiovascular death (HR 1.24, 95% CI 1.02–1.50; *P* = 0.027), cardiovascular death or MI (HR 1.13, 95% CI 1.03–1.24; *P* = 0.009), and the primary ISCHEMIA trial composite end point (HR 1.11, 95% CI 1.01–1.21; *P* = 0.026).

### Additive value of EST data to clinical characteristics and CCTA data for prediction of outcomes

#### All-cause mortality

**Figure 2:**
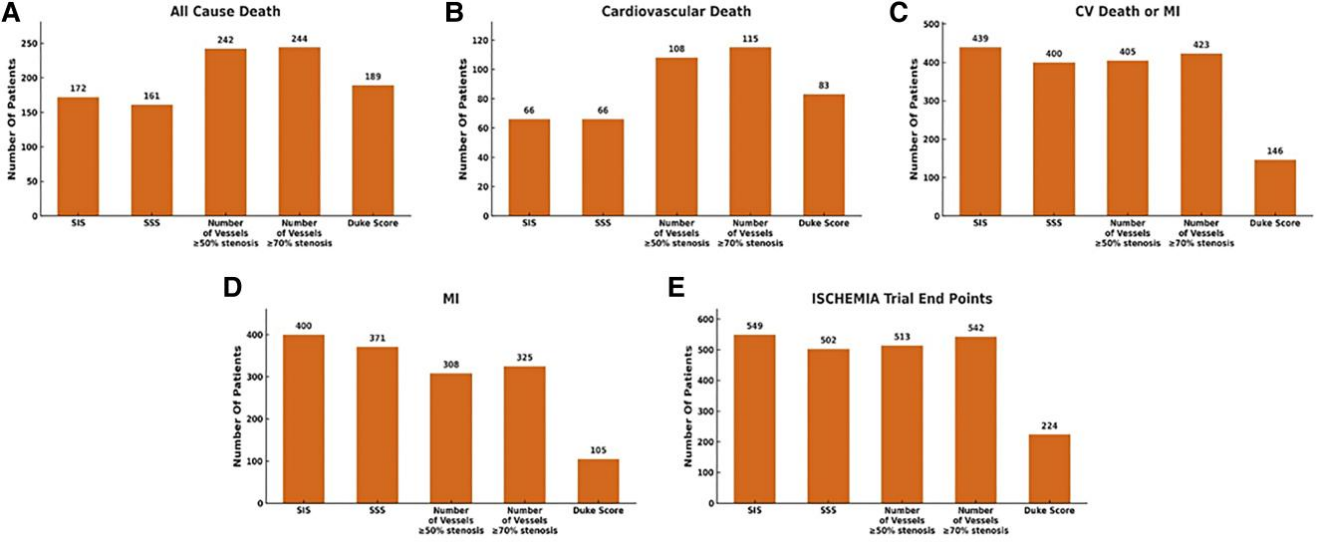
Number of patients whose predicted risk change by 2% with the addition of exercise data. The Y axis presents the number of patients in whom predicted risk have changed by 2%, SIS (segment involving score), SSS (segment stenosis score), number of vessels ≥ 50% stenosis, number of vessels ≥ 70% stenosis and Duke score are presented in X axis according to primary (*A*) and secondary end points (*B-D*). Peak METs improved our risk stratification for all-cause death, CV death and the ISCHEMIA trial composite end points. ISCHEMIA trail composite end point: cardiovascular death, myocardial infraction, hospitalization for heart failure, unstable angina, or resuscitated cardiac arrest.

The proportion of the total predictive information for prognosis that was contributed by EST data was assessed by adding EST information to models including baseline characteristics and CCTA findings. All models (based on the CCTA parameters of SIS, SSS, number of vessels ≥ 50% or ≥ 70% stenosis, and Duke score) exhibited greater ability to predict all-cause mortality with the addition of exercise information, and this was true for both peak METs and exercise duration (*Table 3*, Supplementary data online, *Table S6*, *Figure 2*, *Graphic abstract*. Adding peak METs to anatomical CCTA data provided incremental information gain of 10%, 10%, 15%, 15%, and 20% for SIS, SSS, number of vessels ≥ 50% or ≥ 70% stenosis, and Duke score, respectively. The additive value of exercise duration ranged between 8% and 25% (see Supplementary data online, *Table S6*). This can be exemplified by looking into the number of patients with a change in predicted risk. For example, adding peak METs to the SIS model resulted in at least 2% change in predicted risk in 172 patients, and at least 5% change in predicted risk in 33 patients (*Graphical Abstract, Figure 2*). Neither age nor sex modified the relationships between EST and CCTA data and death, based on interaction testing. In the C-statistics analysis, adding peak METs to CCTA data of number of vessels ≥ 50% or ≥ 70% stenosis showed up to 2% better accuracy in predicting all-cause death (HR = 0.86, CI 0.76–0.98 and HR 0.86, CI 0.76–0.98, respectively) (see Supplementary data online, *Table S11*).

**Table 3:** Number of participants whose predicted risk of all-cause death changed with the addition of exercise data: peak METs

CCTA and EST data elements included in model with clinical characteristics	*N*	≥2% absolute change	≥5% absolute change	Proportion of new information with addition of exercise data^a^
**Segment involvement score**				
Peak METs	1455	172	33	0.1
**Segment stenosis score**				
Peak METs	1455	161	29	0.1
**Number of vessels ≥ 50% stenosis**				
Peak METs	1864	242	39	0.15
**Number of vessels ≥ 70% stenosis**				
Peak METs	1864	244	40	0.15
**Duke score**				
Peak METs	1255	189	37	0.2

Abbreviations: EST, exercise stress test; CCTA, coronary computed tomography angiography; METs, metabolic equivalence of task.

^a^Proportion of the total predictive information in CCTA + exercise + baseline model that was added by including exercise data, e.g. 0.1 = 10% of the total predictive information.

^b^Adjusted for the following variables: age at randomization, sex, region, diabetes, hypertension, current smoker, prior MI, prior revascularization, new or increasing angina, eGFR, ejection fraction, BMI.

#### Secondary outcomes

Adding peak METs to the CCTA anatomical models resulted in better prediction of all secondary outcomes (*Graphical Abstract,Tables 4–7, Figure 2*). The same trend was noted for exercise time. However, the addition of exercise time to the number of vessels ≥ 50% or ≥ 70% stenosis for predicting MI and adding exercise time to SSS for predicting CV death or MI did not improve outcome prediction (see Supplementary data online, *Tables S7*–*S10*). In general, adding peak METs to CCTA anatomical data showed a more pronounced effect than the addition of exercise time. An exception was noted for the Duke score for which adding exercise time improved the ability to predict CV death (*Table 4*, Supplementary data online, *Table S7*). In assessing the risk of CV death, using baseline characteristics, EST and CCTA data (SIS, SSS, number of vessels ≥ 50% or ≥ 70% stenosis), adding peak METs provided 8–13% incremental information, while the addition of exercise duration to clinical characteristics and CCTA resulted in 3–7% incremental predictive information. Nonetheless, for the Duke score, peak METs and exercise duration showed 9% and 15% new information, respectively (*Graphical Abstract, Table 4*, Supplementary data online, *Table S7*, *Figure 2*). The strongest effect of peak METs to predict outcome was more pronounced in the models with the number of vessels ≥ 50% or ≥ 70% stenosis in which adding peak METs changed the predicted probability of a CV death by at least 2% in 108 and 115 patients respectively, or by 5% in 10 and 10 patients respectively (*Graphical Abstract*, *Figure 2*). Prediction of MI and the composite of CV death or MI were improved by adding peak METs to baseline characteristics and CCTA findings models with new information ranging 11–17% for MI (*Table 6*, Supplementary data online, *Table S9*) and 6–15% for CV death or MI (*Graphical Abstract*, *Table 5*, Supplementary data online, *Table S8*, *Figure 2*). A considerable effect (new information ranging 8–16%) on the assessment of risk of the combined end point of cardiovascular death, MI, or hospitalization for heart failure, unstable angina, or resuscitated cardiac arrest was noted by adding peak METs (*Graphical Abstract*, *Table 7*, *Figure 2*). The most pronounced effect was seen in the addition of peak METs to the SIS model, which provided 16% incremental information and resulted in a change in the predicted probability of the combined endpoint by at least 2% and 5% in 549 and 101 patients, respectively. The addition of exercise time to anatomical models showed information gain of 2–6% (see Supplementary data online, *Table S10*). In the C-statistics analysis, adding peak METs to CCTA findings, had improved the prediction of MI, death or MI, and ISCHEMIA trial combined end points by up to 1.2% (see Supplementary data online, *Table S11*) but not for CV death.

**Table 4:** Number of participants whose predicted risk of cardiovascular death changed with the addition of exercise data: peak METs

CCTA and EST data elements included in model with clinical characteristics	*N*	≥2% absolute change	≥5% absolute change	Proportion of new information with addition of exercise data^a^
**Segment involvement score**				
Peak METs	1455	66	7	0.08
**Segment stenosis score**				
Peak METs	1455	66	7	0.08
**Number of vessels ≥ 50% stenosis**				
Peak METs	1864	108	10	0.13
**Number of vessels ≥ 70% stenosis**				
Peak METs	1864	115	10	0.13
**Duke score**				
Peak METs	1255	83	25	0.09

Abbreviations: EST, exercise stress test; CCTA, coronary computed tomography angiography; METs, metabolic equivalence of task.

^a^Proportion of the total predictive information in CCTA + exercise + baseline model that was added by including exercise data, e.g. 0.1 = 10% of the total predictive information.

^b^Adjusted for the following variables: age at randomization, sex, region, diabetes, hypertension, current smoker, prior MI, prior revascularization, new or increasing angina, eGFR, ejection fraction, BMI.

**Table 5:** Number of participants whose predicted risk of cardiovascular death or MI changed with the addition of exercise data: peak METs

CCTA and EST data elements included in model with clinical characteristics	*N*	≥2% absolute change	≥5% absolute change	Proportion of new information with addition of exercise data^a^
**Segment involvement score**				
Peak METs	1455	439	73	0.14
**Segment stenosis score**				
Peak METs	1455	400	69	0.1
**Number of vessels ≥ 50% stenosis**				
Peak METs	1864	405	43	0.12
**Number of vessels ≥70% stenosis**				
Peak METs	1864	423	42	0.13
**Duke score**				
Peak METs	1255	146	15	0.06

Abbreviations: EST, exercise stress test; CCTA, coronary computed tomography angiography; METs, metabolic equivalence of task.

^a^Proportion of the total predictive information in CCTA + exercise + baseline model that was added by including exercise data, e.g. 0.1 = 10% of the total predictive information.

^b^Adjusted for the following variables: age at randomization, sex, region, diabetes, hypertension, current smoker, prior MI, prior revascularization, new or increasing angina, eGFR, ejection fraction, BMI.

**Table 6:** Number of participants whose predicted risk of MI changed with the addition of exercise data: peak METs

Data	*N*	≥2% absolute change	≥5% absolute change	Proportion of new information^a^
**Segment involvement score**				
Peak METs	1455	400	64	0.17
**Segment stenosis score**				
Peak METs	1455	371	71	0.13
**Number of vessels ≥ 50% stenosis**				
Peak METs	1864	308	21	0.11
**Number of vessels ≥ 70% stenosis**				
Peak METs	1864	325	23	0.11
**Duke score**				
Peak METs	1255	105	7	0.06

Abbreviations: EST, exercise stress test; CCTA, coronary computed tomography angiography; METs, metabolic equivalence of task.

^a^Proportion of the total predictive information in CCTA + exercise + baseline model that was added by including exercise data, e.g. 0.1 = 10% of the total predictive information.

^b^Adjusted for the following variables: age at randomization, sex, region, diabetes, hypertension, current smoker, prior MI, prior revascularization, new or increasing angina, eGFR, ejection fraction, BMI.

**Table 7:** Number of participants whose predicted risk of cardiovascular death, myocardial infarction, hospitalization for heart failure, unstable angina, or resuscitated cardiac arrest changed with the addition of exercise data: peak MET’s

CCTA and EST data elements included in model with clinical characteristics	*N*	≥2% absolute change	≥5% absolute change	Proportion of new information with addition of exercise data^a^
**Segment involvement score**				
Peak METs	1455	549	101	0.16
**Segment stenosis score**				
Peak METs	1455	502	101	0.12
**Number of vessels ≥ 50% stenosis**				
Peak METs	1864	513	66	0.13
**Number of vessels ≥ 70% stenosis**				
Peak METs	1864	542	73	0.14
**Duke score**				
Peak METs	1255	224	26	0.08

Abbreviations: EST, exercise stress test; CCTA, coronary computed tomography angiography; METs, metabolic equivalence of task.

^a^Proportion of the total predictive information in CCTA + exercise + baseline model that was added by including exercise data, e.g. 0.1 = 10% of the total predictive information.

^b^Adjusted for the following variables: age at randomization, sex, region, diabetes, hypertension, current smoker, prior MI, prior revascularization, new or increasing angina, eGFR, ejection fraction, BMI.

## Discussion

We have shown that functional performance on EST (specifically, estimated peak METs and exercise duration on the Bruce protocol) adds substantial prognostic information to anatomical CCTA findings among patients with CCD, both for all-cause mortality and composite cardiovascular endpoints.

### Functional performance and outcome

In previous EST studies, peak METs achieved and percentage of predicted heart rate were the most significant EST predictors of survival among patients without CAD.^4^ In a study of asymptomatic patients who underwent EST, the peak MET value was found to be a strong predictor for CV morbidity and mortality the when peak METs value was added to an atherosclerosis risk calculator.^5^ In another study of 1472 patients who underwent both EST and invasive coronary angiography for suspected ischaemic CAD, exercise duration improved risk stratification in patients with known coronary anatomy, with exercise duration defined by Bruce stages (3 min each) and maximal heart rate achieved.^19^ Thus, we aimed to assess the additive value of functional performance among patients with CCD, focusing on peak METs on the Bruce protocol, which is a reproducible and reliable measure. Our findings suggest that independent of CAD severity on CCTA, the ability to achieve higher peak METs is associated with better cardiovascular outcomes. Factors related to functional performance, such as physical activity habits, body composition, gait stability, frailty, muscle and bone strength, and pulmonary function,^20,21^ may underlie our observation of incremental prognostic gain.

### EST vs. CCTA

Using EST or CCTA for risk stratification of patients with stable chest pain is well established.^1^ However, few studies have compared the prognostic value of EST and CCTA in patients undergoing both tests. In a sub-analysis of patients who underwent both EST and CCTA in the SCOT-HEART trial, abnormal EST was more strongly associated with revascularization at 1 year than obstructive CAD on CCTA, while CCTA was more predictive of 5-year cardiovascular death or nonfatal MI. However, the independent contribution of EST to risk assessment with CCTA was not reported.^22^ Others have explored whether exercise ECG findings were incremental to CT anatomical findings. Cho *et al*.^6^ retrospectively studied patients with suspected CAD who performed both symptom-limited EST and CCTA. CCTA displayed superior discriminatory ability for 90-day events compared to exercise results ESTs but again, detailed measurement of exercise level achieved was not included in the analysis. Further, not all patients had obstructive CAD. In that study, exercise ECG data improved risk stratification only for patients with moderate to severe stenosis, and CCTA anatomy had greater prognostic utility than treadmill exercise findings across all anatomical disease severity subgroups.

Although METs and exercise time were already known to be predictors for major cardiac events, the added value of exercise performance beyond risk factors and CCTA findings has not been previously studied, particularly in a cohort with a high burden of CAD. Several strengths of the current study should be recognized. Within the ISCHEMIA trial, we were able to study a large group of patients with stable chronic coronary disease, all of whom had at least 50% stenosis of a major epicardial vessel and detailed exercise information. Also, the ISCHEMIA trial employed the use of a core laboratory for EST data that reported detailed findings beyond ST-segment changes and ECG findings such as peak heart rate and blood pressure, peak METs and exercise duration. Consistent with prior studies, in the ISCHEMIA trial neither ischaemia severity nor symptoms provided incremental risk prediction compared to CCTA anatomy. Through this analysis, we reaffirm the clinical value of assessment of treadmill exercise performance to improve risk stratification when added to anatomical findings on CCTA, emphasizing the importance of functional status regardless of the severity of CAD in patients with moderate or severe ischaemia. For better risk stratification, we encourage physicians to consider an EST following CCTA. Referral of patients with reduced exercise capacity to cardiac rehabilitation is recommended.

### Limitations

This analysis is limited by the inclusion criteria of the ISCHEMIA trial, which required moderate or severe ischaemia, no significant left main stenosis, and obstructive CAD with at least 50% stenosis. Our analysis was necessarily limited to participants who had both EST and CCTA, interpretable at our core laboratories. As such, nearly all patients with available data had ST-segment depression, though the maximal degree of ST-segment depression was missing in 56%; thus, our ability to assess the prediction of outcomes according to the presence and amount of ST-segment depression was limited (data collected included only the presence of ST-segment depression ≥ 1.0 mm). In addition, few patients with very severe ischaemia, such as those with a decrease in blood pressure with exercise, were enrolled. However, we were able to provide important analyses for the studied population, emphasizing the additive value of functional performance on prognosis beyond anatomical CAD findings. The CCTA findings did not include plaque characteristics that can have an impact on clinical end points. Another limitation includes no documentation of the reason for EST termination at the site. Nonetheless, the target heart rate achieved was similar in patients who survived or died, supporting similar exercise effort. Adjustment for beta blocker treatment was limited by missing data in 17% of the population, though no significant difference between groups was noted. Patients with a pharmacological functional test were excluded from the final analysis, raising the possibility of selection bias, as those participants likely had lower exercise performance. We also excluded individuals who underwent cycle stress tests to have a more objective comparison of EST markers, such as achieved METs, given that bicycle protocols used at sites were not standardized. In order to include patients who exercised on a modified Bruce protocol, we reduced the exercise time for such patients by 3 min when analyzing exercise time. A final limitation is the estimation of peak METs from a nomogram rather than the direct measurement of peak oxygen consumption, the recognized standard for quantifying aerobic capacity.

## Conclusions

Among patients with CCD who underwent both treadmill EST and CCTA, peak METs on a Bruce protocol provided incremental risk prediction beyond CCTA anatomical findings. Accordingly, risk stratification was improved when findings from both EST and CCTA were integrated.

## Perspective

Clinical Competencies: Guidelines recommend CCTA as an initial test for patients with stable chest pain to help guide medical management in a fashion that improves clinical outcomes. In our secondary analysis from the ISCHEMIA trial, exercise capacity on a treadmill exercise test, as measured by peak METs, provided incremental risk prediction beyond CCTA anatomy alone. Translational Outlook: This suggests the continued importance of assessment of functional capacity in the evaluation of patients with chronic coronary disease, even when coronary anatomy is known.

## Supplementary Material

jeag032_Supplementary_Data

## Data Availability

ISCHEMIA data have been made publicly available on the National Institutes of Health BioData Catalyst website. **ClinicalTrials.gov Identifier:** NCT01471522; https://clinicaltrials.gov/ct2/show/NCT01471522
