# Supplementary material for: Additive prognostic value of functional performance to coronary artery anatomy: the ISCHEMIA trial
Source: Eur Heart J Cardiovasc Imaging. 2026 Feb 4;27(5):952–66. doi: 10.1093/ehjci/jeag032 (PMC13014304; doi:10.1093/ehjci/jeag032)
Supplement: jeag032_Supplementary_Data [file jeag032_supplementary_data.docx]

**Supplementary Data**

**Incremental Value of Exercise Capacity and CCTA (Ben Zekry et al., 2025)**

Table of Contents

[Table S1: Definition of Ischemia Severity. 2](#_Toc167096678)

[Table S2: Univariable and Adjusted Cox Models for Prediction Cardiovascular Death 4](#_Toc167096679)

[Table S3: Univariable and Multivariable Cox Models for Prediction Cardiovascular Death or MI 6](#_Toc167096680)

[Table S4: Univariable and Adjusted Cox Models for Prediction of MI. 8](#_Toc167096681)

[Table S5: Univariable and Adjusted Cox Models for Prediction cardiovascular death, myocardial infarction, hospitalization for heart failure, unstable angina, or resuscitated cardiac arrest. 10](#_Toc167096682)

[Table S6: Number of Participants Whose Predicted Risk of All-cause Death Changed with the Addition of Exercise Data: Exercise Duration. 11](#_Toc167096683)

[Table S7: Number of Participants Whose Predicted Risk of Cardiovascular Death Changed with the Addition of Exercise Data: Exercise Duration. 13](#_Toc167096684)

[Table S8: Number of Participants Whose Predicted Risk of Cardiovascular Death or MI Changed with the Addition of Exercise Data: Exercise Duration. 14](#_Toc167096685)

[Table S9: Number of Participants Whose Predicted Risk of MI Changed with the Addition of Exercise Data: Exercise Duration. 15](#_Toc167096686)

[Table S10: Number of Participants Whose Predicted Risk of Cardiovascular Death, Myocardial Infarction, Hospitalization for Heart Failure, Unstable Angina, or Resuscitated Cardiac Arrest Changed with the Addition of Exercise Data: Exercise Time. 16](#_Toc167096687)

[Appendix I: ISCHEMIA Site Investigators 17](#_Toc167096688)

[Appendix II: ISCHEMIA Committee, CCC, Trial-Related Personnel 92](#_Toc167096689)

# Table S1: Definition of Ischemia Severity.

| **Modality Diagnostic criterion** | **Ischemia Definition** | | |
| --- | --- | --- | --- |
|  | Mild | Moderate | Severe |
| Nuclear perfusion via  SPECT or PET | 5%–9% LV ischemia | 10%–14% LV ischemia | ≥15% LV ischemia |
| Echo | Stress Echocardiography 1–2 segments with stress-induced severe hypokinesis, akinesis, or dyskinesis | Stress Echocardiography 3 segments with stress-induced  severe hypokinesis, akinesis, or dyskinesis | Stress echocardiography ≥4 segments with stress-induced  severe hypokinesis, akinesis, or dyskinesis |
| Exercise Test without Imaging | ST depression 1 mm during exercise or recovery not meeting the moderate or severe ischemia definition or not meeting the symptom criterion. An example would be 1.2 mm ST depression at 10 METs. | Either ECG or functional capacity criteria meeting  the severe ischemia definition, but not both | ST-segment depression 1.5 mm in 2 leads or 2  mm in 1 lead at ≤7 METs, with angina |
| Abbreviations: ECG – electrocardiogram ; LV – left Ventricle, METs - Metabolic Equivalent of Task; PET – positron emission tomography; SPECT – single-photon emission computed tomography; ST – ST segment  Reynolds HR, Shaw LJ, Min JK, et al. Outcomes in the ISCHEMIA Trial Based on Coronary Artery Disease and Ischemia Severity. *Circulation*. 2021;144(13):1024-1038. doi:10.1161/CIRCULATIONAHA.120.049755 | | | |

# Table S2: Univariable and Adjusted Cox Models for Prediction Cardiovascular Death

| **Characteristic** | **Univariable Analysis** | | **Adjusted Analysis** | |
| --- | --- | --- | --- | --- |
|  | **HR (95% CI)** | **P-value** | **HR (95% CI)** | **P-value** |
| **CCTA Findings (≥50% Stenosis)** |  |  |  |  |
| Specific Native Vessels ≥50% Stenosis |  |  |  |  |
| Number of Diseased Vessels (compared to 1-Vessel Disease) |  | 0.034 |  | 0.15 |
| 2 Vessel Disease | 1.25 (0.41, 3.81) |  | 1.13 (0.36, 3.52) |  |
| 3 Vessel Disease | 2.81 (1.07, 7.33) |  | 2.32 (0.87, 6.2) |  |
| Non-evaluable | 1.21 (0.38, 3.81) |  | 1.25 (0.39, 3.98) |  |
| Segment Stenosis Score (per 5-unit increase) | 1.35 (1.13, 1.62) | <.001 | 1.23 (1.02, 1.49) | 0.032 |
| Segment Involvement Score (per 1-unit increase) | 1.17 (1.03, 1.33) | 0.016 | 1.11 (0.98, 1.27) | 0.11 |
| Duke Prognostic Score (compared to 2V ≥50% or 1V ≥70%) |  | 0.09 |  | 0.39 |
| 1V ≥50% | 1.36 (0.15, 12.17) |  | 1.10 (0.11, 10.91) |  |
| 3V ≥50% or 2V ≥70% or ≥70% Proximal LAD | 2.10 (0.66, 6.7) |  | 1.66 (0.51, 5.41) |  |
| 3V ≥70% or 2V ≥70% Including Proximal LAD or LM ≥50% | 3.72 (1.23, 11.22) |  | 2.53 (0.81, 7.94) |  |
| **Exercise Test Findings** |  |  |  |  |
| Ischemia Severity (compared to mild or no ischemia) |  | 0.64 |  | 0.58 |
| Moderate | 0.63 (0.17, 2.38) |  | 0.57 (0.15, 2.2) |  |
| Severe | 0.90 (0.28, 2.92) |  | 0.85 (0.25, 2.89) |  |
| Heart Rate (per 10 bpm increase) |  |  |  |  |
| Rest | 1.07 (0.88, 1.32) | 0.49 | 1.10 (0.88, 1.39) | 0.39 |
| Stress | 0.88 (0.74, 1.05) | 0.16 | 0.89 (0.74, 1.07) | 0.23 |
| Target Heart Rate Achieved | 1.07 (0.54, 2.12) | 0.84 | 0.89 (0.44, 1.81) | 0.75 |
| Systolic Blood Pressure (per 10 mmHg increase) |  |  |  |  |
| Rest | 1.32 (1.10, 1.58) | 0.003 | 1.24 (1.02, 1.5) | 0.027 |
| Stress | 1.12 (0.97, 1.29) | 0.13 | 1.14 (0.98, 1.34) | 0.1 |
| Diastolic Blood Pressure (per 10 mmHg increase) |  |  |  |  |
| Rest | 1.03 (0.74, 1.45) | 0.85 | 0.99 (0.69, 1.42) | 0.98 |
| Stress | 1.07 (0.78, 1.48) | 0.66 | 1.20 (0.82, 1.75) | 0.35 |
| Rate Pressure Product (per 500-unit increase) | 1.01 (0.97, 1.05) | 0.5 | 1.02 (0.98, 1.07) | 0.28 |
| Peak METs Achieved | 0.85 (0.74, 0.99) | 0.035 | 0.85 (0.72, 1) | 0.046 |
| Exercise Duration (per 1-minute increase) | 0.94 (0.84, 1.06) | 0.34 | 0.92 (0.83, 1.03) | 0.14 |
| Symptoms During Stress | 1.15 (0.58, 2.27) | 0.69 | 1.14 (0.55, 2.33) | 0.73 |
| **ECG Changes** |  |  |  |  |
| ST Depression ≥1.0mm | NA | NA | NA | NA |
| Frequent Ventricular Arrhythmia | NA | NA | NA | NA |

*Adjusted model included age, sex, eGFR, ejection fraction and diabetes. Randomized treatment strategy was included as a stratum effect.

Abbreviations: 1V – 1-vessel; 2V- 2-vessel ; CCTA – coronary computed tomography angiography; CI – confidence interval; ECG – electrocardiogram; HR – hazard ratio; LAD – left anterior descending; LM – left main; mmHg- millimeters of mercury; ST – ST segment

# Table S3: Univariable and Multivariable Cox Models for Prediction Cardiovascular Death or MI

| **Characteristic** | **Univariable Analysis** | | **Adjusted Analysis** | |
| --- | --- | --- | --- | --- |
|  | **HR (95% CI)** | **P-value** | **HR (95% CI)** | **P-value** |
| **CCTA Findings (≥50% Stenosis)** |  |  |  |  |
| Specific Native Vessels ≥50% Stenosis |  |  |  |  |
| Number of Diseased Vessels (compared to 1-Vessel Disease) |  | <.001 |  | <.001 |
| 2 Vessel Disease | 1.40 (0.8, 2.46) |  | 1.28 (0.73, 2.25) |  |
| 3 Vessel Disease | 2.89 (1.76, 4.73) |  | 2.66 (1.61, 4.39) |  |
| Non-evaluable | 1.57 (0.9, 2.76) |  | 1.47 (0.83, 2.59) |  |
| Segment Stenosis Score (per 5-unit increase) | 1.30 (1.19, 1.43) | <.001 | 1.27 (1.15, 1.40) | <.001 |
| Segment Involvement Score (per 1-unit increase) | 1.12 (1.05, 1.19) | <.001 | 1.08 (1.02, 1.16) | 0.015 |
| Duke Prognostic Score (compared to 2V ≥50% or 1V ≥70%) |  | 0.01 |  | 0.003 |
| 1V ≥50% | 0.39 (0.09, 1.66) |  | 0.28 (0.06, 1.18) |  |
| 3V ≥50% or 2V ≥70% or ≥70% Proximal LAD | 1.20 (0.73, 1.97) |  | 1.09 (0.66, 1.80) |  |
| 3V ≥70% or 2V ≥70% Including Proximal LAD or LM ≥50% | 1.87 (1.17, 2.99) |  | 1.93 (1.18, 3.15 |  |
| **Exercise Test Findings** |  |  |  |  |
| Ischemia Severity (compared to mild or no ischemia) |  | 0.36 |  | 0.83 |
| Moderate | 1.25 (0.63, 2.47) |  | 1.21 (0.61, 2.40) |  |
| Severe | 0.98 (0.52, 1.87) |  | 1.12 (0.58, 2.16) |  |
| Heart Rate (per 10 bpm increase) |  |  |  |  |
| Rest | 1.01 (0.91, 1.12) | 0.9 | 1.07 (0.96, 1.19) | 0.23 |
| Stress | 0.91 (0.84, 0.99) | 0.038 | 0.95 (0.87, 1.04) | 0.26 |
| Target Heart Rate Achieved | 0.91 (0.66, 1.25) | 0.56 | 0.84 (0.61, 1.18) | 0.32 |
| Systolic Blood Pressure (per 10 mmHg increase) |  |  |  |  |
| Rest | 1.18 (1.08, 1.29) | <.001 | 1.13 (1.03, 1.24) | 0.009 |
| Stress | 1.07 (1.00, 1.14) | 0.05 | 1.03 (0.96, 1.10) | 0.46 |
| Diastolic Blood Pressure (per 10 mmHg increase) |  |  |  |  |
| Rest | 1.02 (0.87, 1.21) | 0.78 | 1.02 (0.86, 1.21) | 0.78 |
| Stress | 1.06 (0.94, 1.2) | 0.15 | 1.06 (0.91, 1.24) | 0.6 |
| Rate Pressure Product (per 500-unit increase) | 1.00 (0.99, 1.02) | 0.077 | 1.01 (0.99, 1.02) | 0.28 |
| Peak METs Achieved | 0.92 (0.86, 0.99) | 0.022 | 0.9 (0.83, 0.97) | 0.007 |
| Exercise Duration (per 1-minute increase) | 0.96 (0.91, 1.02) | 0.17 | 0.94 (0.89, 1.00) | 0.05 |
| Symptoms During Stress | 0.97 (0.70, 1.35) | 0.86 | 1.26 (0.89, 1.77) | 0.19 |
| **ECG Changes** |  |  |  |  |
| ST Depression ≥1.0mm | 1.86 (0.26, 13.33) | 0.54 | 1.45 (0.19, 11.18) | 0.72 |
| Frequent Ventricular Arrhythmia | 2.06 (1.07, 3.97) | 0.032 | 1.52 (0.74, 3.13) | 0.25 |

*Adjusted model included age, sex, eGFR, ejection fraction and diabetes. Randomized treatment strategy was included as a stratum effect.

Abbreviations: 1V – 1-vessel; 2V- 2-vessel ; CCTA – coronary computed tomography angiography; CI – confidence interval; ECG – electrocardiogram; HR – hazard ratio; LAD – left anterior descending; LM – left main; mmHg- millimeters of mercury; ST – ST segment

# Table S4: Univariable and Adjusted Cox Models for Prediction of MI.

| **Characteristic** | **Univariable Analysis** | | **Adjusted Analysis** | |
| --- | --- | --- | --- | --- |
|  | **HR (95% CI)** | **P-value** | **HR (95% CI)** | **P-value** |
| **CCTA Findings (≥50% Stenosis)** |  |  |  |  |
| Specific Native Vessels ≥50% Stenosis |  |  |  |  |
| Number of Diseased Vessels (compared to 1-Vessel Disease) |  | <.001 |  | <.001 |
| 2 Vessel Disease | 1.32 (0.71, 2.45) |  | 1.18 (0.63, 2.2) |  |
| 3 Vessel Disease | 2.86 (1.67, 4.9) |  | 2.63 (1.52, 4.54) |  |
| Non-evaluable | 1.48 (0.8, 2.75) |  | 1.34 (0.72, 2.51) |  |
| Segment Stenosis Score (per 5-unit increase) | 1.31 (1.18, 1.45) | <.001 | 1.28 (1.15, 1.43) | <.001 |
| Segment Involvement Score (per 1-unit increase) | 1.12 (1.05, 1.2) | <.001 | 1.08 (1.01, 1.16) | 0.029 |
| Duke Prognostic Score (compared to 2V ≥50% or 1V ≥70%) |  | 0.036 |  | 0.004 |
| 1V ≥50% | 0.45 (0.11, 1.88) |  | 0.31 (0.07, 1.33) |  |
| 3V ≥50% or 2V ≥70% or ≥70% Proximal LAD | 1.03 (0.60, 1.77) |  | 0.95 (0.55, 1.64) |  |
| 3V ≥70% or 2V ≥70% Including Proximal LAD or LM ≥50% | 1.72 (1.04, 2.84 |  | 1.89 (1.12, 3.19) |  |
| **Exercise Test Findings** |  |  |  |  |
| Ischemia Severity (compared to mild or no ischemia) |  | 0.048 |  | 0.39 |
| Moderate | 1.70 (0.77, 3.77) |  | 1.61 (0.73, 3.59) |  |
| Severe | 1.11 (0.51, 2.39) |  | 1.33 (0.61, 2.9) |  |
| Heart Rate (per 10 bpm increase) |  |  |  |  |
| Rest | 0.98 (0.87, 1.10) | 0.69 | 1.05 (0.93, 1.19) | 0.41 |
| Stress | 0.86 (0.77, 0.95) | 0.013 | 1.02 (0.91, 1.15) | 0.07 |
| Target Heart Rate Achieved | 0.80 (0.57, 1.13) | 0.21 | 0.74 (0.52, 1.06) | 0.097 |
| Systolic Blood Pressure (per 10 mmHg increase) |  |  |  |  |
| Rest | 1.16 (1.05, 1.28) | 0.004 | 1.11 (1, 1.23) | 0.045 |
| Stress | 1.06 (0.99, 1.14) | 0.094 | 1.01 (0.94, 1.09) | 0.82 |
| Diastolic Blood Pressure (per 10 mmHg increase) |  |  |  |  |
| Rest | 1.05 (0.88, 1.26) | 0.59 | 1.04 (0.87, 1.25) | 0.63 |
| Stress | 1.09 (0.94, 1.26) | 0.26 | 1.05 (0.89, 1.22) | 0.58 |
| Rate Pressure Product (per 500-unit increase) | 1.00 (0.98, 1.02) | 0.038 | 1.00 (0.98, 1.02) | 0.22 |
| Peak METs Achieved | 0.93 (0.86, 1) | 0.065 | 0.89 (0.82, 0.97) | 0.009 |
| Exercise Duration (per 1-minute increase) | 0.96 (0.91, 1.03) | 0.26 | 0.94 (0.87, 1) | 0.049 |
| Symptoms During Stress | 0.91 (0.64, 1.3) | 0.61 | 1.28 (0.88, 1.86) | 0.19 |
| **ECG Changes** |  |  |  |  |
| ST Depression ≥1.0mm | 1.47 (0.2, 10.54) | 0.703 | 1.02 (0.13, 7.86) | 0.99 |
| Frequent Ventricular Arrhythmia | 2.80 (1.44, 5.45) | 0.002 | 1.85 (0.89, 3.84) | 0.097 |

*Adjusted model included age, sex, eGFR, ejection fraction and diabetes. Randomized treatment strategy was included as a stratum effect.

Abbreviations: 1V – 1-vessel; 2V- 2-vessel ; CCTA – coronary computed tomography angiography; CI – confidence interval; ECG – electrocardiogram; HR – hazard ratio; LAD – left anterior descending; LM – left main; mmHg- millimeters of mercury; ST – ST segment

# Table S5: Univariable and Adjusted Cox Models for Prediction cardiovascular death, myocardial infarction, hospitalization for heart failure, unstable angina, or resuscitated cardiac arrest.

| **Characteristic** | **Univariable Analysis** | | **Adjusted Analysis** | |
| --- | --- | --- | --- | --- |
|  | **HR (95% CI)** | **P-value** | **HR (95% CI)** | **P-value** |
| **CCTA Findings (≥50% Stenosis)** |  |  |  |  |
| Specific Native Vessels ≥50% Stenosis |  |  |  |  |
| Number of Diseased Vessels (compared to 1-Vessel Disease) |  | <.001 |  | <.001 |
| 2 Vessel Disease | 1.28 (0.75, 2.17) |  | 1.14 (0.67, 1.95) |  |
| 3 Vessel Disease | 2.66 (1.68, 4.23) |  | 2.44 (1.53, 3.91) |  |
| Non-evaluable | 1.43 (0.84, 2.43) |  | 1.34 (0.78, 2.28) |  |
| Segment Stenosis Score (per 5-unit increase) | 1.28 (1.17, 1.4) | <.001 | 1.26 (1.14, 1.38) | <.001 |
| Segment Involvement Score (per 1-unit increase) | 1.11 (1.05, 1.18) | <.001 | 1.08 (1.02, 1.15) | 0.014 |
| Duke Prognostic Score (compared to 2V ≥50% or 1V ≥70%) |  | 0.021 |  | 0.007 |
| 1V ≥50% | 0.56 (0.17, 1.83) |  | 0.41 (0.12, 1.36) |  |
| 3V ≥50% or 2V ≥70% or ≥70% Proximal LAD | 1.23 (0.77, 1.98) |  | 1.16 (0.72, 1.87) |  |
| 3V ≥70% or 2V ≥70% Including Proximal LAD or LM ≥50% | 1.81 (1.15, 2.85) |  | 1.89 (1.17, 3.04) |  |
| **Exercise Test Findings** |  |  |  |  |
| Ischemia Severity (compared to mild or no ischemia) |  | 0.11 |  | 0.5 |
| Moderate | 1.46 (0.74, 2.85) |  | 1.41 (0.72, 2.77) |  |
| Severe | 1.05 (0.55, 2) |  | 1.21 (0.63, 2.32) |  |
| Heart Rate (per 10 bpm increase) |  |  |  |  |
| Rest | 1.01 (0.91, 1.11) | 0.87 | 1.07 (0.97, 1.19) | 0.18 |
| Stress | 0.92 (0.85, 1) | 0.06 | 0.96 (0.88, 1.05) | 0.35 |
| Target Heart Rate Achieved | 0.94 (0.69, 1.28) | 0.68 | 0.87 (0.63, 1.2) | 0.4 |
| Systolic Blood Pressure (per 10 mmHg increase) |  |  |  |  |
| Rest | 1.15 (1.06, 1.26) | 0.001 | 1.11 (1.01, 1.21) | 0.026 |
| Stress | 1.05 (0.99, 1.12) | 0.1 | 1.01 (0.95, 1.08) | 0.72 |
| Diastolic Blood Pressure (per 10 mmHg increase) |  |  |  |  |
| Rest | 1.02 (0.87, 1.2) | 0.8 | 1.03 (0.87, 1.21) | 0.72 |
| Stress | 1.04 (0.91, 1.2) | 0.54 | 1.03 (0.89, 1.19) | 0.71 |
| Rate Pressure Product (per 500-unit increase) | 1.00 (0.99, 1.02) | 0.082 | 1.01 (0.99, 1.02) | 0.31 |
| Peak METs Achieved | 0.92 (0.86, 0.99) | 0.017 | 0.90 (0.83, 0.97) | 0.004 |
| Exercise Duration (per 1-minute increase) | 0.96 (0.91, 1.01) | 0.13 | 0.94 (0.88, 0.99) | 0.03 |
| Symptoms During Stress | 0.99 (0.72, 1.36) | 0.94 | 1.28 (0.92, 1.78) | 0.15 |
| **ECG Changes** |  |  |  |  |
| ST Depression ≥1.0mm | 1.98 (0.28, 14.16) | 0.5 | 1.51 (0.2, 11.52) | 0.69 |
| Frequent Ventricular Arrhythmia | 1.94 (1.01, 3.73) | 0.048 | 1.46 (0.72, 2.98) | 0.3 |

*Adjusted model included age, sex, eGFR, ejection fraction and diabetes. Randomized treatment strategy was included as a stratum effect.

Abbreviations: 1V – 1-vessel; 2V- 2-vessel ; CCTA – coronary computed tomography angiography; CI – confidence interval; ECG – electrocardiogram; HR – hazard ratio; LAD – left anterior descending; LM – left main; mmHg- millimeters of mercury; ST – ST segment

# Table S6: Number of Participants Whose Predicted Risk of All-cause Death Changed with the Addition of Exercise Data: Exercise Duration.

| **CCTA and EST Data Elements included in Model with Clinical Characteristics** | **N** | **≥2% Absolute Change** | **≥5% Absolute Change** | **Proportion of New Information with Addition of Exercise Data*** |
| --- | --- | --- | --- | --- |
| **Segment Involvement Score** |  |  |  |  |
| Exercise Duration | 1445 | 96 | 17 | 0.08 |
| **Segment Stenosis Score** |  |  |  |  |
| Exercise Duration | 1445 | 86 | 11 | 0.09 |
| **Number of Vessels ≥50% stenosis** |  |  |  |  |
| Exercise Duration | 1852 | 141 | 18 | 0.13 |
| **Number of Vessels ≥70% stenosis** |  |  |  |  |
| Exercise Duration | 1852 | 142 | 22 | 0.13 |
| **Duke Score** |  |  |  |  |
| Exercise Duration | 1247 | 219 | 52 | 0.25 |

*Proportion of the total predictive information in CCTA + Exercise + Baseline model that was added by including exercise data. Exercise duration for patients who were tested using a modified Bruce protocol was actual exercise duration minus 3 minutes.

†Adjusted for the following variables: Age at randomization, Sex, Region, Diabetes, Hypertension, Current smoker, Prior MI, Prior revascularization, New or increasing angina, eGFR, Ejection fraction, BMI.

Abbreviations: EST – exercise stress test; CCTA – coronary computed tomography angiography

# Table S7: Number of Participants Whose Predicted Risk of Cardiovascular Death Changed with the Addition of Exercise Data: Exercise Duration.

| **CCTA and EST Data Elements included in Model with Clinical Characteristics** | **N** | **≥2% Absolute Change** | **≥5% Absolute Change** | **Proportion of New Information with Addition of Exercise Data*** |
| --- | --- | --- | --- | --- |
| **Segment Involvement Score** |  |  |  |  |
| Exercise Duration | 1445 | 8 | 0 | 0.03 |
| **Segment Stenosis Score** |  |  |  |  |
| Exercise Duration | 1445 | 7 | 0 | 0.04 |
| **Number of Vessels ≥50% stenosis** |  |  |  |  |
| Exercise Duration | 1852 | 19 | 0 | 0.06 |
| **Number of Vessels ≥70% stenosis** |  |  |  |  |
| Exercise Duration | 1852 | 21 | 0 | 0.07 |
| **Duke Score** |  |  |  |  |
| Exercise Duration | 1247 | 94 | 18 | 0.15 |

*Proportion of the total predictive information in CCTA + Exercise + Baseline model that was added by including exercise data. Exercise duration for patients who were tested using a modified Bruce protocol was actual exercise duration minus 3 minutes.

†Adjusted for the following variables: Age at randomization, Sex, Region, Diabetes, Hypertension, Current smoker, Prior MI, Prior revascularization, New or increasing angina, eGFR, Ejection fraction, BMI.

Abbreviations: EST – exercise stress test; CCTA – coronary computed tomography angiography

# Table S8: Number of Participants Whose Predicted Risk of Cardiovascular Death or MI Changed with the Addition of Exercise Data: Exercise Duration.

| **CCTA and EST Data Elements included in Model with Clinical Characteristics** | **N** | **≥2% Absolute Change** | **≥5% Absolute Change** | **Proportion of New Information with Addition of Exercise Data*** |
| --- | --- | --- | --- | --- |
| **Segment Involvement Score** |  |  |  |  |
| Exercise Duration | 1445 | 204 | 26 | 0.02 |
| **Segment Stenosis Score** |  |  |  |  |
| Exercise Duration | 1445 | 181 | 26 | 0 |
| **Number of Vessels ≥50% stenosis** |  |  |  |  |
| Exercise Duration | 1852 | 138 | 14 | 0.01 |
| **Number of Vessels ≥70% stenosis** |  |  |  |  |
| Exercise Duration | 1852 | 151 | 16 | 0.01 |
| **Duke Score** |  |  |  |  |
| Exercise Duration | 1247 | 264 | 32 | 0.02 |

*Proportion of the total predictive information in CCTA + Exercise + Baseline model that was added by including exercise data. Exercise duration for patients who were tested using a modified Bruce protocol was actual exercise duration minus 3 minutes.

†Adjusted for the following variables: Age at randomization, Sex, Region, Diabetes, Hypertension, Current smoker, Prior MI, Prior revascularization, New or increasing angina, eGFR, Ejection fraction, BMI.

Abbreviations: EST – exercise stress test; CCTA – coronary computed tomography angiography

# Table S9: Number of Participants Whose Predicted Risk of MI Changed with the Addition of Exercise Data: Exercise Duration.

| **Data** | **N** | **>= 2% Absolute Change** | **>= 5% Absolute Change** | **Proportion of New Information*** |
| --- | --- | --- | --- | --- |
| **Segment Involvement Score** |  |  |  |  |
| Exercise Duration | 1445 | 230 | 30 | 0.05 |
| **Segment Stenosis Score** |  |  |  |  |
| Exercise Duration | 1445 | 213 | 28 | 0.01 |
| **Number of Vessels ≥ 50% stenosis** |  |  |  |  |
| Exercise Duration | 1852 | 120 | 9 | -0.01 |
| **Number of Vessels ≥ 70% stenosis** |  |  |  |  |
| Exercise Duration | 1852 | 125 | 11 | -0.01 |
| **Duke Score** |  |  |  |  |
| Exercise Duration | 1247 | 209 | 24 | 0.01 |

*Proportion of the total predictive information in CCTA + Exercise + Baseline model that was added by including exercise data. Negative values indicate the predictive information that was lost by including exercise data. Exercise duration for patients who were tested using a modified Bruce protocol was actual exercise duration minus 3 minutes.

†Adjusted for the following variables: Age at randomization, Sex, Region, Diabetes, Hypertension, Current smoker, Prior MI, Prior revascularization, New or increasing angina, eGFR, Ejection fraction, BMI.

Abbreviations: EST – exercise stress test; CCTA – coronary computed tomography angiography

# Table S10: Number of Participants Whose Predicted Risk of Cardiovascular Death, Myocardial Infarction, Hospitalization for Heart Failure, Unstable Angina, or Resuscitated Cardiac Arrest Changed with the Addition of Exercise Data: Exercise Time.

| **CCTA and EST Data Elements included in Model with Clinical Characteristics** | **N** | **≥2% Absolute Change** | **≥5% Absolute Change** | **Proportion of New Information with Addition of Exercise Data*** |
| --- | --- | --- | --- | --- |
| **Segment Involvement Score** |  |  |  |  |
| Exercise Duration | 1445 | 324 | 49 | 0.06 |
| **Segment Stenosis Score** |  |  |  |  |
| Exercise Duration | 1445 | 289 | 40 | 0.02 |
| **Number of Vessels ≥50% stenosis** |  |  |  |  |
| Exercise Duration | 1852 | 227 | 28 | 0.03 |
| **Number of Vessels ≥70% stenosis** |  |  |  |  |
| Exercise Duration | 1852 | 243 | 28 | 0.03 |
| **Duke Score** |  |  |  |  |
| Exercise Duration | 1247 | 328 | 44 | 0.06 |

*Proportion of the total predictive information in CCTA + Exercise + Baseline model that was added by including exercise data. Exercise duration for patients who were tested using a modified Bruce protocol was actual exercise duration minus 3 minutes.

†Adjusted for the following variables: Age at randomization, Sex, Region, Diabetes, Hypertension, Current smoker, Prior MI, Prior revascularization, New or increasing angina, eGFR, Ejection fraction, BMI.

Abbreviations: EST – exercise stress test; CCTA – coronary computed tomography angiography

**Table S11: Hazard Ration and C-statistics Analysis**

| **CCTA and EST Data Elements included in Model with Clinical Characteristics** | **N** | **Exercise Data Variable HR (95% CI)** | **CCTA**  **C-Statistic** | **Exercise**  **C-Statistic** | **C-Statistic Difference** |
| --- | --- | --- | --- | --- | --- |
| ***End Point: All Cause Death***  **Segment Involvement Score** |  |  |  |  |  |
| Exercise Duration  Peak Mets | 1445  1455 | 0.93 (0.84, 1.03)  0.87 (0.76, 1) | 0.7326  0.7326 | 0.738  0.7428 | 0.0054  0.0102 |
| **Segment Stenosis Score** |  |  |  |  |  |
| Exercise Duration  Peak Mets | 1445  1455 | 0.93 (0.84, 1.03)  0.88 (0.76, 1.01) | 0.7314  0.7314 | 0.7361  0.7403 | 0.0047  0.0089 |
| **Number of Vessels ≥50% stenosis** |  |  |  |  |  |
| Exercise Duration  Peak Mets | 1852  1864 | 0.92 (0.83, 1.01)  0.86 (0.76, 0.98) | 0.6978  0.6978 | 0.7088  0.7139 | 0.011  0.0161 |
| **Number of Vessels ≥70% stenosis** |  |  |  |  |  |
| Exercise Duration  Peak Mets | 1852  1864 | 0.92 (0.83, 1.01)  0.86 (0.76, 0.98) | 0.7023  0.7023 | 0.7109  0.7175 | 0.0086  0.0152 |
| ***End Point: Cardiovascular Death***  **Segment Involvement Score** |  |  |  |  |  |
| Exercise Duration  Peak Mets | 1445  1455 | 0.98 (0.87, 1.09)  0.89 (0.75, 1.06) | 0.743  0.743 | 0.7473  0.7601 | 0.0043  0.0171 |
| **Segment Stenosis Score** |  |  |  |  |  |
| Exercise Duration  Peak Mets | 1445  1455 | 0.97 (0.87, 1.09)  0.89 (0.75, 1.06) | 0.7509  0.7509 | 0.7538  0.7617 | 0.0029  0.0108 |
| **Number of Vessels ≥50% stenosis** |  |  |  |  |  |
| Exercise Duration  Peak Mets | 1852  1864 | 0.96 (0.86, 1.07)  0.87 (0.74, 1.02) | 0.7092  0.7092 | 0.7158  0.7295 | 0.0066  0.0203 |
| **Number of Vessels ≥70% stenosis** |  |  |  |  |  |
| Exercise Duration  Peak Mets | 1852  1864 | 0.96 (0.86, 1.07)  0.87 (0.74, 1.02) | 0.7159  0.7159 | 0.7232  0.7343 | 0.0073  0.0184 |
|  |  |  |  |  |  |
| ***End Point:***  ***Cardiovascular Death or MI***  **Segment Involvement Score** |  |  |  |  |  |
| Exercise Duration  Peak Mets | 1445  1455 | 0.94 (0.88, 1.01)  0.9 (0.83, 0.98) | 0.6426  0.6426 | 0.6471  0.6532 | 0.0045  0.0106 |
| **Segment Stenosis Score** |  |  |  |  |  |
| Exercise Duration  Peak Mets | 1445  1455 | 0.95 (0.89, 1.01)  0.9 (0.83, 0.98) | 0.6635  0.6635 | 0.6617  0.669 | -0.0018  0.0055 |
| **Number of Vessels ≥50% stenosis** |  |  |  |  |  |
| Exercise Duration  Peak Mets | 1852  1864 | 0.96 (0.9, 1.02)  0.92 (0.85, 0.99) | 0.6421  0.6421 | 0.6426  0.6481 | 0.0005  0.006 |
| **Number of Vessels ≥70% stenosis** |  |  |  |  |  |
| Exercise Duration  Peak Mets | 1852  1864 | 0.96 (0.9, 1.02)  0.91 (0.85, 0.98) | 0.6406  0.6406 | 0.6407  0.6468 | 0.0001  0.0062 |
|  |  |  |  |  |  |
| ***End Point: MI***  **Segment Involvement Score**  Exercise Duration  Peak Mets | 1445  1455 | 0.93 (0.86, 1)  0.88 (0.81, 0.97) | 0.6516  0.6516 | 0.6587  0.6591 | 0.0071  0.0075 |
| **Segment Stenosis Score** |  |  |  |  |  |
| Exercise Duration  Peak Mets | 1445  1455 | 0.93 (0.86, 1)  0.89 (0.81, 0.98) | 0.6692  0.6692 | 0.6685  0.6716 | -0.0007  0.0024 |
| **Number of Vessels ≥50% stenosis** |  |  |  |  |  |
| Exercise Duration  Peak Mets | 1852  1864 | 0.95 (0.89, 1.02)  0.91 ((0.84, 0.99) | 0.6625  0.6625 | 0.6629  0.6663 | 0.0004  0.0038 |
| **Number of Vessels ≥70% stenosis** |  |  |  |  |  |
| Exercise Duration  Peak Mets | 1852  1864 | 0.95 (0.89, 1.02)  0.91 (0.84, 0.99) | 0.661  0.661 | 0.6608  0.6642 | -0.0002  0.0032 |
| ***End Point: Cardiovascular Death, Myocardial Infarction, Hospitalization for Heart Failure, Unstable Angina, or Resuscitated Cardiac Arrest***  **Segment Involvement Score**  Exercise Duration  Peak Mets | 1445  1455 | 0.93 (0.87, 1)  0.89 (0.82, 0.96) | 0.644  0.644 | 0.6488  0.6543 | 0.0048  0.0103 |
| **Segment Stenosis Score** |  |  |  |  |  |
| Exercise Duration  Peak Mets | 1445  1455 | 0.93 (0.88,1)  0.89 (0.82,0.97) | 0.6625  0.6625 | 0.6622  0.6682 | -0.0003  0.0057 |
| **Number of Vessels ≥50% stenosis** |  |  |  |  |  |
| Exercise Duration  Peak Mets | 1852  1852 | 0.95 (0.9, 1.01)  0.91 (0.84, 0.98) | 0.6461  0.6461 | 0.6486  0.6517 | 0.0025  0.0056 |
| **Number of Vessels ≥70% stenosis** |  |  |  |  |  |
| Exercise Duration  Peak Mets | 1852  1864 | 0.95 (0.89, 1.01)  0.91 (0.84, 0.98) | 0.644  0.644 | 0.6466  0.6493 | 0.0026  0.0053 |

# Appendix I: ISCHEMIA Site Investigators

| Country (No.Randomizations) | Investigator(s) | Study Coordinator(s) | City & State | Institution (No. Randomizations) |
| --- | --- | --- | --- | --- |
|  |  |  | (if applicable) |  |
| *United States (853) |  |  |  |  |
| Country Leader |  |  |  |  |
| David J. Maron, MD |  |  |  |  |
| Regional Leader for VA Sites | |  |  |  |
| William E. Boden, MD |  |  |  |  |
|  | Kreton Mavromatis, MD | John Doan, MD | Decatur, GA | Atlanta VA Medical Center (139) |
|  | Jason Linefsky, MD | Raven Lee, CCRP |  |  |
|  |  | Risha Patel |  |  |
|  | Todd Miller, MD | So Yang Cho | Rochester, MN | Mayo Clinic (50) |
|  |  | Susan Milbrandt |  |  |
|  |  | Dawn Shelstad |  |  |
|  | Subhash Banerjee, MD | Preeti Kamath, BDS, MHA, CCRP | Dallas, TX | V.A. North Texas Health Care System (35) |
|  |  | Ishita Tejani, BDS, MS, MSPH |  |  |
|  | Harmony R. Reynolds, MD | Stanley E. Cobos, BA | New York, NY | NYU Langone Medical Center-Bellevue Hospital (26) |
|  | Jonathan D. Newman, MD, MPH | Kirsten J. Quiles, MS |  |  |
|  | Sripal Bangalore, MD | Raven R. Dwyer, MPH |  |  |
|  | Robert  M. Donnino, MD | Dalisa Espinosa, MBS |  |  |
|  | Lawrence M. Phillips, MD |  |  |  |
|  | Muhamed Saric, MD, PhD |  |  |  |
|  | Khaled Abdul-Nour, MD | Allison Schley, BS | Detroit, MI | Henry Ford Health System (21) |
|  |  | Heather Golden |  |  |
|  | Peter H. Stone, MD | Hermine Osseni, MS | Boston, MA | Brigham & Women's Hospital, Harvard Medical School (21) |
|  |  | Charlene Wiyarand |  |  |
|  |  | Peter Douglass, BA |  |  |
|  |  | Hayley Pomeroy, BA |  |  |
|  |  | Alexandra Craft, BA |  |  |
|  |  | Bethany Harvey, BA |  |  |
|  | James J. Jang, MD | Olivia Anaya | San Jose, CA | Kaiser Permanente San Jose (18) |
|  | Gennie Yee, MD | Phoebe Goold, RN |  |  |
|  | Steven Weitz, MD | Steven Giovannone | Schenectady, NY | Cardiology Associates of Schenectady P.C. (17) |
|  |  | Lori Pritchard, RN |  |  |
|  | Suzanne Arnold, MD | Rosann Gans, RN | Kansas City, MO | Saint Luke's Hospital (17) |
|  | James Henry O’Keefe, Jr, MD (PI from 2012-2016) | Paul Kennedy, RN |  |  |
|  | Michael D. Shapiro, DO | Shobana Ganesan, PhD | Portland, OR | Oregon Health & Science University (17) |
|  |  | David Schlichting, LPN |  |  |
|  |  | Aynun Naher |  |  |
|  | Mohammad El-Hajjar, MD |  | Albany, NY | Albany Medical Center Hospital (16) |
|  | Mandeep S. Sidhu, MD, MBA |  |  |  |
|  | Steven A. Fein, MD | Wendy L. Stewart, MS |  |  |
|  | Mikhail T. Torosoff, MD, PhD | Kristin M. Salmi, BS |  |  |
|  | Radmila Lyubarova, MD |  |  |  |
|  | Sulagna Mookherjee, MD |  |  |  |
|  | Krzysztof Drzymalski, MD |  |  |  |
|  | Edward O. McFalls, MD, PhD |  | Minneapolis, MN | Minneapolis VAMC (15) |
|  | Santiago A. Garcia, MD |  |  |  |
|  | Stefan C. Bertog, MD | Debra K. Johnson, RN |  |  |
|  | Rizwan A. Siddiqui, MD | Rebekah R. Herrmann, RN |  |  |
|  | Areef Ishani, MD |  |  |  |
|  | Ronnell A. Hansen, MD |  |  |  |
|  | Michel Georges Khouri, MD | Kristine Arges | Durham, NC | Duke University Medical Center (15) |
|  |  | Melissa LeFevre |  |  |
|  |  | Jennifer Tomfohr |  |  |
|  | Jonathan L. Goldberg, MS, MD | Kimberly Ann Byrne | Cleveland, OH | Louis Stokes Cleveland Veterans Affairs Medical Center (14) |
|  |  | Taissa Zappernick |  |  |
|  | Richard Goldweit, MD | Sallie Canada | Englewood, NJ | Englewood Hospital and Medical Center (13) |
|  |  | Meghana Kakade |  |  |
|  |  | Patricia Mieses |  |  |
|  |  | Stanley E. Cobos, BA | Brooklyn, NY | NYU-HHC Woodhull Hospital (12) |
|  |  | Raven R. Dwyer, MPH |  |  |
|  | Ronny A. Cohen, MD | Dalisa Espinosa, MBS |  |  |
|  | Brooks Mirrer, MD | Kirsten J. Quiles, MS |  |  |
|  | Victor Navarro, MD | Magdalena Rantinella, BS |  |  |
|  |  | Jessica Rodriguez, BS |  |  |
|  |  | Olivia Mancilla, BS |  |  |
|  | David E. Winchester, MD, MS | Susan Stinson, RN | Gainesville, FL | Malcom Randall VAMC (11) |
|  | Marvin Kronenberg, MD | Terry Weyand | Nashville, TN | Vanderbilt University Medical Center (11) |
|  | Philip Rogal, MD | Sherron C. Crook |  |  |
|  | Christopher McFarren, MD |  |  |  |
|  | John F. Heitner, MD | Jean Ho | Brooklyn, NY | New York -Presbyterian/Brooklyn Methodist Hospital (10) |
|  |  | Saadat Khan |  |  |
|  |  | Mahmoud Mohamed |  |  |
|  | Ira M. Dauber, MD | Mary R. Soltau, RN | Littleton, CO | South Denver Cardiology Associates, P.C. (10) |
|  |  | Delsa K. Rose, RN |  |  |
|  |  | Rebecca J. Wimmer, RN |  |  |
|  |  | Kathy E. Siegel, RN |  |  |
|  |  | Susan Derbyshire |  |  |
|  | Charles Cannan, MD | Michelle Dixon | Portland, OR | Providence Heart and Vascular Institute (10) |
|  |  | Gerald Leonard |  |  |
|  | Sriram Sudarshan, MD | Ciarra Heard, LVN | Wichita Falls, TX | Wichita Falls Heart Clinic (9) |
|  |  | Viviana Gabriel, LVN |  |  |
|  |  | Sukie Desire |  |  |
|  | Puja K. Mehta, MD |  | Atlanta, GA | Emory University (9) |
|  | Michael McDaniel, MD | Fauzia Rashid, PhD |  |  |
|  | Stamatios Lerakis, MD | Senait Asier |  |  |
|  | Arshed Quyyumi, MD | Keyur Patel |  |  |
|  | Nanette K. Wenger, MD |  |  |  |
|  | Chester M. Hedgepeth, MD, PhD | Jennifer Gillis, APRN | Warwick, RI | Kent Hospital (9) |
|  | Heather Hurlburt, MD | Megan Manocchia, RN |  |  |
|  | Alan Rosen, MD | Susan Moore, RN |  |  |
|  |  | Elizabeth Congdon |  |  |
|  | Zakir Sahul, MD | Gail Brandt | Ypsilanti, MI | Michigan Heart, PC (9) |
|  |  | Nora Marchelletta |  |  |
|  |  | Kristina Wippler |  |  |
|  | David Booth, MD | Yvonne Taul, RN | Lexington, KY | University of Kentucky (8) |
|  | Steve Leung, MD | Jennifer Isaacs, MS |  |  |
|  | Ahmed Abdel-Latif, MD, PhD | Viktoria Bulkley, RN |  |  |
|  | Hassan Reda, MD | Caroline Rodgers |  |  |
|  | Khaled Ziada, MD |  |  |  |
|  | Sampoornima Setty, MD | Kimberly E. Halverson, RHIT | La Crosse, WI | Gundersen Lutheran Medical Center (8) |
|  |  | Christine Roraff, RN |  |  |
|  |  | Jonean Thorsen, RN |  |  |
|  | Rajat S. Barua, MD, PhD | Amarachi Ojajuni | Kansas City, MO | Kansas City VA Medical Center (8) |
|  |  | Oni Olurinde |  |  |
|  |  | Kamalakar Surineni |  |  |
|  | Fadi Hage, MD | Badhma Valaiyapathi, MD | Birmingham, AL | UAB Vascular Biology and Hypertension Program (8) |
|  | Christiano Caldeira, MD |  |  |  |
|  | James E. Davies, MD |  |  |  |
|  | Massoud Leesar, MD |  |  |  |
|  | Jaekyeong Heo, MD |  |  |  |
|  | Amy Iskandrian, MD |  |  |  |
|  | Firas Al Solaiman, MD |  |  |  |
|  | Satinder Singh, MD |  |  |  |
|  | Khaled Dajani, MD | Carol M. Kartje, BSN | Maywood, IL | Loyola University Medical Center (8) |
|  | Mohammad El-Hajjar, MD |  | Albany, NY | Samuel Stratton VA Medical Center of Albany NY (7) |
|  | Paul Der Mesropian, MD |  |  |  |
|  | Joseph Sacco, MD | Michele Rawlins, NP |  |  |
|  | Brian McCandless, MD | Jennifer Thomson, MA |  |  |
|  | Marisa Orgera, MD |  |  |  |
|  | Mandeep S. Sidhu, MD, MBA (2012-2016 ) |  |  |  |
|  |  | Mary Colleen Rogge, RN | Cincinnati, OH | Cincinnati VA Medical Center (7) |
|  | Imran Arif, MD | Julie Bunke , BA |  |  |
|  | Hanan Kerr, MD | Kendra Unterbrink , PA |  |  |
|  |  | Jacqueline Fannon, RN |  |  |
|  |  | Cynthia Burman, NP |  |  |
|  | Jorge F. Trejo (Gutierrez), MD | Marcia F. Dubin, CCRP | Jacksonville, FL | Mayo Clinic Florida (7) |
|  | Gerald Fletcher, MD |  |  |  |
|  | Gary E. Lane, MD |  |  |  |
|  | Lynn M. Neeson, DNP |  |  |  |
|  | Pragnesh P. Parikh, MD |  |  |  |
|  | Peter M. Pollak, MD |  |  |  |
|  | Brian P. Shapiro, MD |  |  |  |
|  | Kevin Landolfo, MD |  |  |  |
|  | Anthony Gemignani, MD | Sarah Beaudry, RN | White River Junction, VT | VAMC-White River Junction (7) |
|  | Daniel O'Rourke, MD |  |  |  |
|  | Judith L. Meadows, MD | Stephanie A. Tirado, RN | West Haven, CT | VA Connecticut Healthcare System (7) |
|  |  | Janet Halliday |  |  |
|  |  | Pamela Julian |  |  |
|  | Jason T. Call, MD | Stephanie, M. Lane, RN, BSN, CCRN | Winchester, VA | Winchester Cardiology and Vascular Medicine, PC (7) |
|  |  | Jennifer L. Stanford, RN, MSN |  |  |
|  | Joseph Hannan, MD |  | Worcester, MA | Saint Vincent Hospital at Worcester Medical Center (7) |
|  | Robert Bojar, MD | Patricia Arsenault, RN |  |  |
|  | Deepti Kumar, MD | Pamela Sigel, RN |  |  |
|  | John Mukai, MD |  |  |  |
|  | Edward T. Martin, MS, MD | Miriam Brooks | Tulsa, OK | Oklahoma Heart Institute (7) |
|  | Gabriel Vorobiof, MD | Ladda Douangvila | Los Angeles, CA | Ronald Reagan UCLA Medical Center (7) |
|  |  | Rubine Gevorgyan |  |  |
|  | Alec Moorman, MD | Fatima Ranjbaran, RN | Seattle, WA | University of Washington Medical Center (7) |
|  |  | Bryn Smith, BS |  |  |
|  |  | Carly Ohmart |  |  |
|  | Scott Kinlay, MBBS, PhD |  | West Roxbury, MA | VA Boston Healthcare System (6) |
|  | Robert J. Hamburger, MD |  |  |  |
|  | Thomas P. Rocco, MD | Samantha Ly, MA |  |  |
|  | Deepak L. Bhatt, MD, MPH | Margot C. Quinn, BA |  |  |
|  | Kevin Croce, MD, PhD | Sara Temiyasathit, PhD |  |  |
|  | Jacquelyn A Quin, MD | Jacquelyn Do, MPH |  |  |
|  | Jati Anumpa, MD | Desiree Tobin, MPH |  |  |
|  | Marco Zenati, MD, MSc |  |  |  |
|  | David P Faxon, MD |  |  |  |
|  | Glenn Rayos, MD | Jennifer Langdon | Daytona Beach, FL | Daytona Heart Group (6) |
|  |  | Marcia Werner Bayer |  |  |
|  | Ashraf Seedhom, MD | Amanda O'Malley | Albany, NY | Capital Cardiology Associates (6) |
|  | Lance Sullenberger, MD | Erin Orvis |  |  |
|  | Gregory Kumkumian, MD | Mandy Murphy, RN | Bethesda, MD | NIH Heart Center at Suburban Hospital (6) |
|  |  | Ann Greenberg, RN |  |  |
|  |  | Margaret Iraola, RN |  |  |
|  | Steven P. Sedlis, MD | Leandro C.Maranan, CCRC | New York, NY | VA New York Harbor Health Care System (6) |
|  | Robert M. Donnino, MD |  |  |  |
|  | Jeffrey Lorin, MD |  |  |  |
|  | Jacqueline E. Tamis-Holland, MD | Ammy Malinay, RN | Ridgewood, NJ | Mount Sinai Saint Luke's Hospital (6) |
|  | Robert Kornberg, MD |  |  |  |
|  | Robert Leber, MD |  |  |  |
|  | Souheil Saba, MD | Candice P. Edillo, RN | Southfield, MI | Providence - Providence Park Hospital (6) |
|  | Michael W. Lee, MD |  |  |  |
|  | Delano R. Small, MD |  |  |  |
|  | Wassim Nona, MD |  |  |  |
|  | Patrick B. Alexander, MD |  |  |  |
|  | Iram Rehman, MD |  |  |  |
|  | Umesh Badami, MD | Ann Ostrander, RN | Saginaw, MI | Covenant Medical Center, Inc. (5) |
|  |  | Stephanie Wasmiller, RN |  |  |
|  | Kevin Marzo, MD | Wendy Drewes, RN | Mineola, NY | NYU Winthrop (5) |
|  |  | Dipti Patel, RN |  |  |
|  | Inga H. Robbins, MD |  | Pomona, NJ | AtlantiCare Regional Medical Center (5) |
|  | Howard A. Levite, MD | Jackie M White, RN, BSN CCRC |  |  |
|  | Sanjay Shetty, MD | Alison Hallam |  |  |
|  | Mayuri Patel, MD |  |  |  |
|  | Glenn S. Hamroff, MD | Benjamin J Spooner, RPA-C | Cortlandt Manor, NY | NYP Medical Medical Group Hudson Valley Cardiology (5) |
|  |  | Linda M Hollenweger, LPN,CCRC |  |  |
|  | Raymond W. Little, MD | Holly Little | Houston, TX | Houston Heart & Vascular Associates (5) |
|  | Brandi D. Zimbelman, FNP-C | Tiffany Little |  |  |
|  | Charles Y. Lui, MD | Nona A Eskelson, RN | Salt Lake City, UT | Salt Lake City VA Medical Center (4) |
|  | Brigham R. Smith, MD |  |  |  |
|  | Daniel P. Vezina, MD, MSC |  |  |  |
|  | Lillian L. Khor, MBBCh, MSc |  |  |  |
|  | Josephine D. Abraham, MD, MPH |  |  |  |
|  | David A. Bull, MD |  |  |  |
|  | Stephen H. McKellar, MD, MSc |  |  |  |
|  | David Booth, MD | Yvonne Taul, RN | Lexington, KY | Lexington VA Medical Center (4) |
|  | John Kotter, MD | Caroline Rodgers, RN |  |  |
|  | Ahmed Abdel-Latif, MD, PhD | Jennifer Isaacs, MS |  |  |
|  |  | Viktoria Bulkley |  |  |
|  | Bob Hu, MD | Renee Kaneshiro | Palo Alto, CA | Palo Alto Medical Foundation Research Institute (4) |
|  | Arthur J. Labovitz, MD |  | Tampa, FL | University of South Florida (4) |
|  | Michael Berlowitz, MD | Bonnie J. Kirby, RN, MSN |  |  |
|  | Philip Rogal, MD | Nhi N. Tran, MS |  |  |
|  | Christopher McFarren, MD | Catherine Jahrsdorfer, RN, BSN |  |  |
|  | Fadi Matar, MD |  |  |  |
|  | Christiano Caldeira, MD |  |  |  |
|  | David J. Maron, MD |  | Stanford, CA | Stanford University School of Medicine (4) |
|  | Fatima Rodriguez, MD, MPH | Reem Yunis, PhD |  |  |
|  | Ingela Schnittger, MD | Jhina Patro |  |  |
|  | William F. Fearon, MD |  |  |  |
|  | Prakash Deedwania, MD | Antonia Vega | Fresno, CA | UCSF - Fresno Community Regional Medical Center (4) |
|  | Kiran Reddy, MD |  |  |  |
|  | Joseph Sweeny, MD | Hugo Bloise-Adames | New York, NY | Icahn School of Medicine at Mount Sinai (4) |
|  |  | Santa Jimenez |  |  |
|  |  | Nicole Saint Vrestil |  |  |
|  |  | Reyna Bhandari |  |  |
|  | Christopher Spizzieri, MD | Danielle Schade | Camp Hill, PA | Holy Spirit Hospital Cardiovascular Institute (4) |
|  |  | Roxanne Yost |  |  |
|  | Claudia P Hochberg, MD | Paula Beardsley | Boston, MA | Boston Medical Center (4) |
|  |  | Denise Fine |  |  |
|  | William D. Salerno, MD | Jana Tancredi, RN, MA/MSN, CCRN | Saddle Brook, NJ | Hackensack University Medical Center (4) |
|  |  | Patricia Arakelian |  |  |
|  |  | Susan Mathus |  |  |
|  |  | Deborah O'Neill |  |  |
|  | Ray Wyman, MD | Joy Burkhardt, CCRP | Torrance, CA | Torrance Memorial Medical Center (4) |
|  |  | Suellen Hosino, RN, BSN, CCRP |  |  |
|  |  |  |  |  |
|  |  | Oksana A. Lubyanaya, BA | Santa Ana, CA | Coastal Heart Medical Group (4) |
|  |  | Jose D. Salas, BS |  |  |
|  | Amer Zarka, MD | Maria Aguirre |  |  |
|  | Anil V. Shah, MD | Manu Dhawan |  |  |
|  |  | Diana Parra |  |  |
|  |  | Tri Tran |  |  |
|  | Thomas Haldis, DO | Catherine Weick, BSRT(R)(VI) | Fargo, ND | Sanford Health (4) |
|  |  | Katie Fowler-Lehman, BSN |  |  |
|  |  | Natalie Spitzer, BSN |  |  |
|  |  | Casey Riedberger |  |  |
|  |  | Catherine Weick |  |  |
|  | Jeffrey A. Kohn, MD | Stanley E. Cobos, BA | New York, NY | NYU New York Medical Associates (4) |
|  |  | Raven R. Dwyer, MPH |  |  |
|  |  | Dalisa Espinosa, MBS |  |  |
|  |  | Kirsten J. Quiles, MS |  |  |
|  | Saket Girotra, MD | Carrie Drum, RN | Iowa City, IA | University of Iowa Hospitals and Clinics (4) |
|  |  | Kimberly Miller-Cox, RN |  |  |
|  |  | Amy Ollinger, RN |  |  |
|  | Omar Almousalli, MD | Elizabeth Capasso-Gulve | Fairview Heights, IL | Advanced Heart Care Group (4) |
|  |  | Alaine Melanie Loehr |  |  |
|  |  | Marlowe Mosley |  |  |
|  | Mayil S. Krishnam, MD | Shirin Heydari, MS | Orange, CA | University of California Irvine Medical Center (3) |
|  | Jeffrey C. Milliken, MD | Andrea M. Lundeen, MA |  |  |
|  | Pranav M. Patel, MD | Edgar Karanjah, MD |  |  |
|  | Arnold H. Seto, MD | Wanda C. Marfori, MD |  |  |
|  | Kevin T. Harley, MD | Eduardo Hernandez-Rangel, MD |  |  |
|  | Michael A. Gibson, MD | Pam Singh |  |  |
|  | Byron J. Allen, MD |  |  |  |
|  | Rita Coram, MD | Anne Marie Webb, BSN | Louisville, KY | University of Louisville (3) |
|  |  | Ellie Fridell, BS |  |  |
|  |  | Heidi Wilson, BS |  |  |
|  | Sabu Thomas, MD, MSc | Angela Kim, BS | Rochester, NY | University of Rochester (3) |
|  | Ronald G Schwartz, MD, MS | Patrick Wilmot, BS |  |  |
|  | Wei Chen, MD, MS |  |  |  |
|  | Mahfouz El Shahawy, MD | Ramona Stevens | Sarasota, FL | Cardiovascular Center of Sarasota (3) |
|  | James Stafford, MD | Loriane Black | Baltimore, MD | University of Maryland Medical Center (3) |
|  | William B. Abernethy, MD | Amber B. Hull, RN | Asheville, NC | Asheville Cardiology Associates (3) |
|  |  | Olivia J. Lim, RN |  |  |
|  |  | Helen C. Tucker |  |  |
|  |  | Natasha C. Putnam, RN |  |  |
|  |  | Linda L. Hall |  |  |
|  |  | Tia Cauthren |  |  |
|  |  | Trish Tucker |  |  |
|  | Andrew Zurick, MD | Hollie Horton | Nashville, TN | Saint Thomas Hospital (3) |
|  |  | Jan Orga |  |  |
|  | Thomas M. Meyer, MD | Joyce R. White, MSN NP-C | Lynchburg, VA | Stroobants Cardiovascular Center (3) |
|  | Ronald G. Morford, MD | Cynthia Baumann, RN |  |  |
|  | Bruce Rutkin, MD | Vidya Seeratan | Manhasset, NY | Northwell Health - Manhasset (3) |
|  | Sabahat Bokhari, MD | Magnolia Jimenez | New York, NY | Columbia University Medical Center (3) |
|  | Seth I. Sokol, MD | Cidney Schultz, RN | Bronx, NY | Jacobi Medical Center (3) |
|  | Jay Meisner, MD | Jeanne Russo, RN |  |  |
|  | Ihab Hamzeh, MD |  | Houston, TX | Baylor College of Medicine (3) |
|  | Arunima Misra, MD | Zohra Huda, RN, BSN, CCRP |  |  |
|  | Matthew Wall Jr., MD | Araceli Boan |  |  |
|  | Veronica Lenges De Rosen, MD |  |  |  |
|  | Mahboob Alam, MD |  |  |  |
|  | Michael C. Turner, MD | Christine R Hinton | Lake Charles, LA | Cardiovascular Specialists of Southwest Louisiana (3) |
|  | Thomas J. Mulhearn, MD |  |  |  |
|  | Arnold P. Good, MD | Beth A. Archer, BSN, RN | Columbus, OH | Ohio Health Grant Medical Center (3) |
|  |  | Julia S. Dionne, BA |  |  |
|  |  | Cheryl A. Allardyce, BSN, RN |  |  |
|  |  | Lindsey N. Sikora, BSN, RN |  |  |
|  |  | Jennifer H. Czerniak, RN |  |  |
|  |  | Jennifer A. Mull, MSN, RN |  |  |
|  |  | Elizabeth Ferguson |  |  |
|  |  | Frances Laube |  |  |
|  | Nicolas W. Shammas, MD, MS | Gail A Shammas, BSN, RN | Davenport, IA | Midwest Cardiovascular Research Foundation (3) |
|  |  | Lori Christensen |  |  |
|  |  | Holly Park |  |  |
|  | Robert Chilton, MD | Joan Hecht | San Antonio, TX | Audie Murphy V.A. (2) |
|  | Patricia K. Nguyen, MD | Davis Vo, BS | Palo Alto, CA | VA Palo Alto Healthcare System (2) |
|  |  | James Hirsch |  |  |
|  | Matthew Jezior, MD | Jody Bindeman | Bethesda, MD | Walter Reed National Military Medical Center (2) |
|  |  | Sara Salkind |  |  |
|  |  | Dalisa Espinosa, MBS | Providence, RI | Miriam Hospital (2) |
|  |  | Lori-Ann Desimone, BSN |  |  |
|  | Paul C. Gordon, MD | Lina Felix-Stern |  |  |
|  | Thomas Crain, MD | Jassira Gomes |  |  |
|  |  | Catherine Gordon, BSN |  |  |
|  | Robert Stenberg, MD | Aimee Mann | Johnstown, PA | Conemaugh Valley Memorial Hospital (2) |
|  |  | Theresa McCreary |  |  |
|  | Ronald P. Pedalino, MD | Stanley E. Cobos, BA | Brooklyn, NY | NYU-HHC Kings County Hospital Center (2) |
|  |  | Raven R. Dwyer, MPH |  |  |
|  |  | Dalisa Espinosa, MBS |  |  |
|  |  | Kirsten J. Quiles, MS |  |  |
|  | Joseph Wiesel, MD | Stanley E. Cobos, BA | Flushing, NY | New York University - Langone Cardiovascular Associates (2) |
|  |  | Raven R. Dwyer, MPH |  |  |
|  |  | Dalisa Espinosa, MBS |  |  |
|  |  | Kirsten J. Quiles, MS |  |  |
|  | George J. Juang, MD | Candace Gopaul, BS | Brooklyn, NY | Coney Island Hospital (2) |
|  |  | Karen Hultberg |  |  |
|  |  | Tauqir Huk |  |  |
|  |  | Afshan Hussain |  |  |
|  | Mohammed Al-Amoodi, MD | Yesenia Zambrano, BS | Yuma, AZ | Yuma Regional Medical Center (2) |
|  |  | Sarah Medina Rodriguez |  |  |
|  |  | Trudie Milner |  |  |
|  | David Wohns, MD | Abbey Mulder, RN | Grand Rapids, MI | Spectrum Health (2) |
|  |  | Stacie Van Oosterhout, MEd |  |  |
|  | Ellis W. Lader, MD | Martha Meyer, RN, MSN | Kingston, NY | Mid Valley Cardiology (1) |
|  | Michael Mumma, MD | Nancy L. Clapp, RN, BA, CCRC | Sarasota, FL | Sarasota Memorial Hospital (1) |
|  |  | Heather Barrentine |  |  |
|  | Lekshmi Dharmarajan , MD | Jenne M. Jose, PA | Bronx, NY | NYU-HHC Lincoln Medical and Mental Health Center (1) |
|  |  | Stanley E. Cobos, BA |  |  |
|  |  | Raven R. Dwyer, MPH |  |  |
|  |  | Dalisa Espinosa, MBS |  |  |
|  |  | Kirsten J. Quiles, MS |  |  |
|  |  | Jenne Manchery |  |  |
|  | Joseph F.X. McGarvey Jr, MD | Vera McKinney, RN | Doylestown, PA | Doylestown Health Cardiology (1) |
|  |  | Linda Schwarz, RN |  |  |
|  | Thomas R. Downes, MD (till Dec. 2016) | Scott M. Kaczkowski | Loveland, CO | Medical Center of the Rockies (1) |
|  | Gary J. Luckasen, MD (from Dec. 2016) | Adam J. Jaskowiak |  |  |
|  |  | Joel Klitch |  |  |
|  | Benjamin Cheong, MD | Debra Dees | Houston, TX | Baylor St. Luke's Medical Center (1) |
|  | Srinivasa Potluri, MD | Precilia Vasquez | Plano, TX | Baylor Research Institute at Legacy Heart Center (1) ** |
|  | Ronald A. Mastouri, MD |  | Indianapolis, IN | Indiana University/Krannert Institute of Cardiology (1) |
|  | Jeffery A. Breall, MD, PhD | Elise L. Hannemann, RN,CCRC |  |  |
|  | George E. Revtyak, MD | Judy Mae Foltz, RN,CCRC |  |  |
|  | Jonathan W. Bazeley, MD |  |  |  |
|  | Dayuan Li, MD | Emily DeRosa | St. Paul, MN | HealthEast Saint Joseph's Hospital (1) |
|  |  | Beth Jorgenson |  |  |
|  |  | Joyce Riestenberg-Smith |  |  |
|  | Kenneth Giedd, MD |  | New York, NY | Beth Israel Medical Center (1) |
|  | Wayne Old, MD | Rebecca Bariciano | Chesapeake, VA | Cardiovascular Associates, Ltd. (1) |
|  | Francis Burt, MD |  | Bethlehem, PA | Saint Luke's Hospital and Health Network (1) |
|  | Kozhaya Sokhon, MD | Jessica Waldron | Sugar land, TX | Medicus Alliance Clinical Research Org., Inc. (1) |
|  |  | Michelle Mayon |  |  |
|  | Deepika Gopal, MD |  | Plano, TX | The Heart Hospital Baylor (1) |
|  | Uma S. Valeti, MD | Gretchen Ann Peichel, RN | Minneapolis, MN | University of Minnesota (1) |
|  | Jon Kobashigawa, MD | Brandy Starks | Beverly Hills, CA | Cedars Sinai Medical Center (1) |
|  |  | Lucilla Garcia |  |  |
|  |  | Maria Thottam |  |  |
| India (941) |  |  |  |  |
| Country Leader |  |  |  |  |
| Balram Bhargava, DM |  |  |  |  |
|  |  | Anjali Anand, MSc | Calicut | Government Medical College (208) |
|  | Sajeev Chakanalil Govindan, MD, DNB, DM, PhD | Janitha Raj, B.Tech |  |  |
|  | Rajesh Gopalan Nair, MD, DNB, DM | Reshma Ravindran, MSc |  |  |
|  |  | Rajalekshmi VS, MSc, MScCRRA |  |  |
|  | Cholenahally Nanjappa Manjunath, MD, DM | Nandita Nataraj, BE(Biotech) PGDICRCDM | Bengaluru | Sri Jayadeva Institute of Cardiovascular Sciences and Research (149) |
|  | Nagaraja Moorthy, MD, DM | Soundarya Nayak, BE(Biotech) PGDICRCDM | |  |
|  | Satvic Cholenahally Manjunath, MD,DM | Mahevamma Mylarappa, GNM (General Nursing) | |  |
|  | Suryaprakash Narayanappa, MBBS |  |  |  |
|  | Neeraj Pandit, MD, DM | Sheromani Bajaj | New Delhi | Dr Ram Manohar Lohia Hospital (101) |
|  | Ranjit Kumar Nath, MD, DM | Vandana Yadav, Msc,PGDACR |  |  |
|  |  | Girish Mishra, Msc, PGDACR |  |  |
|  | S.K. Dwivedi, DM | Roma Tewari, PG | Lucknow | King George's Medical University, Department of Cardiology (100) |
|  | V.S. Narain, DM | Meenakshi Mishra, PG |  |  |
|  | Sharad Chandra, DM | Shivali Patel |  |  |
|  |  | Suman Singh, PG |  |  |
|  | Gurpreet S. Wander, DM |  | Ludhiana | Hero DMC Heart Institute, Dayanand Medical College and Hospital (83) |
|  | Rohit Tandon, MD |  |  |  |
|  | Sarju Ralhan, M.Ch (CTVS) | Baljeet Kaur, MSc (Biotechnology) |  |  |
|  | Naved Aslam, DM | Sonika Gupta , MBA, B. Pharmacy |  |  |
|  | Abhishek Goyal, DM |  |  |  |
|  | Balram Bhargava, DM | Chandini Suvarna, BDS | New Delhi | All India Institute Of Medical Sciences (67) |
|  | G.Karthikeyan, DM |  |  |  |
|  | S.Ramakrishnan, DM |  |  |  |
|  | Sandeep Seth, DM |  |  |  |
|  | Rakesh Yadav, DM |  |  |  |
|  | Sandeep Singh, DM |  |  |  |
|  | Ambuj Roy, DM |  |  |  |
|  | Neeraj Parakh, DM |  |  |  |
|  | Sunil Kumar Verma, DM |  |  |  |
|  | Rajiv Narang, DM |  |  |  |
|  | Sundeep Mishra, DM |  |  |  |
|  | Nitish Naik, DM |  |  |  |
|  | Gautam Sharma, DM |  |  |  |
|  | Shiv Kumar Choudhary, M.Ch |  |  |  |
|  | Chetan Patel, DNB |  |  |  |
|  | Gurpreet Gulati, MD |  |  |  |
|  | Sanjeev Sharma, MD |  |  |  |
|  | V K Bahl, DM |  |  |  |
|  | Anoop Mathew, MD | Binoy Mannekkattukudy Kurian | Kolenchery | MOSC Medical College Hospital (39) |
|  | Eapen Punnoose, MD |  |  |  |
|  | Milind Avdhoot Gadkari, MD | Sheetal Rupesh Karwa, BHMS | Pune | KEM Hospital Pune (35) |
|  | Siddharth Gadage, MD DNB | Suvarna Kolhe, MSc |  |  |
|  | Tapan Umesh Pillay, BHMS MSc |  |  |  |
|  | Santhosh Satheesh, MBBS, MD, DM | R. J. Vindhya, B.Sc. (Bio-Technology), MSc(Bio-Informatics) | Pondicherry | Jawaharlal Institute of Postgraduate Medical Education & Research (JIPMER) (31) |
|  |  | Peeyush Jain, MD | New Delhi | Fortis Escort Heart Institute |
|  |  | Ashok Seth, MD |  | -31 |
|  |  | Zile Singh Meharwal, MD |  |  |
|  | Atul Mathur, MD | Atul Verma, MD |  |  |
|  | Upendra Kaul, MD | Mona Bhatia, MD |  |  |
|  |  | Ankush Sachdeva, MD |  |  |
|  |  | Thounaojam Indira Devi, RN |  |  |
|  |  | Nungshi Jungla, RN |  |  |
|  | Johann Christopher, MD, DNB | K. Manjula Rani, MSc. | Hyderabad | Gurunanak CARE Hospital (27) |
|  | Rajeev Menon, MD, DNB | M. Sowjanya Reddy, BSc |  |  |
|  | Nirmal Kumar, MD, DNB | K. Preethi, BSc |  |  |
|  | Abraham Oomman, MD,DM,DNB | Rinu R sidh, MSc(Clinical Research) | Chennai | Apollo Research and Innovation (23) |
|  | Robert Mao, MD, DM | Ramakrishnan T., B.Tech(Biotechnology) | |  |
|  | Hilda Solomon, PhD | Rajesh Francis, MSc(Clinical Research) | |  |
|  | Sudhir Naik, MD, DM | Vamshi Priya P., MSc | Hyderabad | Apollo Research & Innovations (13) |
|  | Sajeeda Parveen Khan, MBBS, (Dip.Card) |  |  |  |
|  | Johann Christopher, MD | Kotiboinna Preethi | Hyderabad | CARE Nampally (11) |
|  | Nirmal Kumar, MD |  |  |  |
|  | Purvez Grant, MD | Shweta Hande, BHMS, PGDCR | Pune | Ruby Hall Clinic,Grant Medical Foundation (10) |
|  |  | Poonam Sonawane, B.ScMicrobiology, ACCR | |  |
|  | Ranjan Kachru, MD | Abhishek Dubey | New Delhi | Fortis Healthcare Fl.t Lt. Rajan Dhall Hospital (4) |
|  |  | Kavita Rawat |  |  |
|  | Ajit Kumar VK, MD, DM |  | Trivandrum | Sree Chitra Tirunal Institute for Medical Sciences and Technology (3) |
|  | Sanjay Ganapathi, MD, DM |  |  |  |
|  | Jayakumar K, MS, M.Ch | Vineeth CP |  |  |
|  | Harikrishnan Sivadasanpillai, MD, DM | Manas Chacko, RN |  |  |
|  | Bijulal Sasidharan, MD, DM | Suresh Babu |  |  |
|  | Kapilamoorthy TR, MD |  |  |  |
|  | Johann Christopher, MD | Sowjanya Reddy | Hyderabad | CARE Hospital (3) |
|  | Praneeth Polamuri, MD | Manjula Rani |  |  |
|  | Upendra Kaul, MD | Priyadarshani Arambam | New Delhi | Batra Hospital and Medical Research Centre (BHMRC) (3) |
|  |  | Bebek Singh |  |  |
| United Kingdom (539) |  |  |  |  |
| Country Leaders |  |  |  |  |
| Roxy Senior, MBBS, MD, DM |  |  |  |  |
| Keith AA Fox, MBChB *(past)* |  |  |  |  |
| Country Coordinators |  |  |  |  |
| Grace M. Young , MSc, BSc (Hons) | |  |  |  |
| Kathryn Carruthers *(past)* |  |  |  |  |
|  | Roxy Senior, MBBS, MD, DM |  | Harrow | Northwick Park Hospital Harrow/ Royal Brompton Hospital London (202) |
|  | Ahmed Elghamaz, MB BCh |  |  |  |
|  | Sothinathan Gurunathan, MBChB |  |  |  |
|  | Nikolaos Karogiannis, MBBS | Grace M. Young , MSc, BSc (Hons) |  |  |
|  | Benoy N Shah, MD, MBBS, BSc (Hons) | Christopher Kinsey |  |  |
|  | Richard HJ Trimlett, MBBS, CCST | Raisa Kavalakkat, MSc, BSc, RN |  |  |
|  | Michael B Rubens, LRCP, MRCS, MBBS, DMRD | Jo Evans, RN |  |  |
|  | Edward D Nicol, MD, BMedSci, MBBS, DTM&H | Ikraam Hassan, RN |  |  |
|  | Tarun K Mittal, MD |  |  |  |
|  | Reinette Hampson, BSc (Hons), BA (Hons) | |  |  |
|  | Reto Andreas Gamma, MBBS | Sarah Williams, RN | Chelmsford | Broomfield Hospital (39) |
|  |  | Kim Holland, RN |  |  |
|  |  | Karen Swan, RN |  |  |
|  | Mark A de Belder, MD | Bev Atkinson, RN | Middlesbrough | The James Cook University Hospital, Middlesbrough (37) |
|  | Jeet Thambyrajah, MD |  |  |  |
|  | Thuraia Nageh, BSc(Hons) MBBS MD MRCP | Swapna Kunhunny, MRes Clin Res, BSc (N), RN | Westcliffe on Sea | Southend University Hospital (34) |
|  | John R Davies, MBBS, PhD |  |  |  |
|  | Steven J. Lindsay, MD | Craig Atkinson, RN | Bradford | Bradford Royal Infirmary (20) |
|  | John Kurian, MD | Carita Krannila, RN |  |  |
|  | Haqeel Jamil, MD | Manitha Vinod, RN |  |  |
|  | Osama Raheem, MD |  |  |  |
|  | Angela Hoye, MD | Lisa Chaytor | Cottingham | The University of Hull/Castle Hill Hospital (19) |
|  |  | Leanne Cox |  |  |
|  |  | Julie Morrow |  |  |
|  |  | Kay Rowe |  |  |
|  | Patrick Donnelly, MD | Stephanie Kelly, RN | Belfast | South Eastern Health and Social Care (17) |
|  | Bernardas Valecka, MD | Susan Regan, RN |  |  |
|  |  | Dawn Turnbull |  |  |
|  | Anoop Chauhan, MD | Catherine Fleming | Blackpool | Blackpool Teaching Hospitals (16) |
|  |  | Arijit Ghosh |  |  |
|  |  | Karen Gratrix |  |  |
|  |  | Stephen Preston |  |  |
|  | Craig Barr, MD | Anne Cartwright | Dudley | Russells Hall Hospital (15) |
|  | Khaled Alfakih, MBBS, MD | Abigail Knighton, BSc., PG Dip. | London | King's College NHS Foundation Hospital (14) |
|  | Jonathan Byrne, PhD | Katherine Martin, RGN, Dip. N, MSc |  |  |
|  | Ian Webb, PhD, MA |  |  |  |
|  | Peter Henriksen, PhD, MB ChB, BSc(Hons) | Laura Flint, RGN | Edinburgh | Royal Infirmary of Edinburgh (13) |
|  |  | James Harrison, BSc(Hons), PG dip |  |  |
|  | Peter OKane, MD | Nicki Lakeman | Bourneouth | Royal Bournemouth Hospital (13) |
|  |  | Anja Ljubez |  |  |
|  | Ramesh de Silva, MB ChB, MD |  | Bedford | Bedford Hospital NHS Trust (11) |
|  | Dwayne S. G. Conway, MD | Judith Wright | Wakefield | Pinderfields Hospital (11) |
|  |  | Donna Exley |  |  |
|  | Alexander A Sirker, MB BChir, PhD |  | London |  |
|  |  | Mervyn Andiapen, RN |  | University College London Hospitals NHS Foundation Trust |
|  |  | Amy J. Richards, BSc |  | BartsHealth NHS Trust |
|  |  |  |  |  |
|  |  |  |  | -11 |
|  | Stephen P Hoole, MD | Lisa Wong, MSc | Cambridge | Papworth Hospital (10) |
|  | Fraser N. Witherow, MD | Melanie J. Munro, RGN | Dorchester | Dorset County Hospital (8) |
|  | Nicola Johnston, MB, Bch BAO, MRCP, MD | | Belfast | Belfast Trust (7) |
|  | Mark Harbinson, MB, Bch BAO, MRCP, MD | Michelle McEvoy, RN |  |  |
|  | Simon Walsh, MB, Bch BAO, MD | Caroline Brown, RN |  |  |
|  | Hanna Douglas, MB, Bch BAO, MRCP, MD | |  |  |
|  | Matthew Luckie, MD | Thabitha Charles | Manchester | Central Manchester University Hospital (7) |
|  |  | Laurel Kolakaluri |  |  |
|  |  | Hannah Phillips |  |  |
|  | Jolanta Sobolewska, MD | Louise Morby, RN | Oldham | The Pennine Acute Hospitals NHS Trust (6) |
|  |  | Karen Hallett, RN |  |  |
|  |  | Carolyn Corbett, RN |  |  |
|  |  | Lynne Winstanley |  |  |
|  | Paramjit Jeetley, MD | Angelique Smit, RN | London | Royal Free London NHS Foundation Trust (6) |
|  | Niket Patel, MD |  |  |  |
|  | Tushar Kotecha, MBChB, Mpharm |  |  |  |
|  | Christopher Travill, MBBS, MD | Susan Gent, SRN RGN | Luton | Luton and Dunstable University Hospital NHS FT (5) |
|  | Iqbal Karimullah, MBBS | Nafisa Hussain, BSc |  |  |
|  | Mahmud Al-Bustami, MBBS |  |  |  |
|  | Denise Braganza, MD | Fiona Haines | Peterborough | Peterborough City Hospital (5) |
|  |  | Joanne Taaffe |  |  |
|  | Robert Henderson, MD | Jane Burton | Nottingham | Nottingham University Hospitals (4) |
|  | Kate Pointon, MBBS | Maria Colton |  |  |
|  | Surendra Naik, PhD | Rachel King |  |  |
|  | Thomas Mathew, MBBS, MD, DM |  |  |  |
|  |  | Ammani Brown, MSc BA RN | Clydebank | University of Glasgow (4) |
|  |  | Andrew Docherty, RN |  |  |
|  | Colin Berry, BSc MB ChB, PhD | Lisa McCloy, RN |  |  |
|  | Damien Collison, MB ChB | Kate Robb, RN |  |  |
|  | Giles Roditi, MB ChB | Craig Paterson, PhD |  |  |
|  |  | Wenda Crawford, RN |  |  |
|  |  | Joanne Kelly, RN |  |  |
|  |  | Lorraine McGregor, RN |  |  |
|  | Andrew J Moriarty, BSc MB PhD | Anne Mackin, RN, BSc | Craigavon | Cardiovascular Research Unit, Craigavon Area Hospital (2) |
|  | Jason D. Glover, MBBS | Janet P Knight, RN | Basingstoke | Hampshire Hospitals NHS Foundation Trust (2) |
|  | Jiwan Pradhan, MBBS |  |  |  |
|  | Ghada Mikhail, MD | Tuhina Bose | London | Imperial College Healthcare NHS Trust (1) |
|  | Darrel P. Francis, MD, MA |  |  |  |
| *Canada (447) |  |  |  |  |
| Country Leaders |  |  |  |  |
| Vladimir Dzavik, MD |  |  |  |  |
| Shaun Goodman, MD, MSc |  |  |  |  |
| Gilbert Gosselin, MD |  |  |  |  |
|  | Gilbert Gosselin, MD | Anna Proietti, RN | Montreal, QC | Montreal Heart Institute (90) |
|  |  | Myriam Brousseau, RN |  |  |
|  |  | Magalie Corfias, RN |  |  |
|  |  | Patricia Blaise |  |  |
|  |  | Luc Harvey |  |  |
|  | Ariel Diaz, MD |  | Trois-Rivieres, QC | Centre Hospitalier de Regional Trois-Rivieres (71) |
|  | Philippe Rheault, MD |  |  |  |
|  | Miguel Barrero, MD |  |  |  |
|  | Carl-Éric Gagné, MD | Patricia Alarie |  |  |
|  | Yanek Pépin-Dubois, MD | Linda Arcand |  |  |
|  | Ricardo Costa, MD | Isabelle Roy |  |  |
|  | Ying Tung Sia, MD | Estelle Montpetit |  |  |
|  | Catherine Lemay, MD |  |  |  |
|  | Alejandro Gisbert, MD |  |  |  |
|  | Pierre Gervais, MD |  |  |  |
|  | Alain Rheault, MD |  |  |  |
|  |  | Katia Drouin, RN | Terrebonne, QC | CISSSL - Hopital Pierre-Le Gardeur (42) |
|  | Denis Carl Phaneuf, MD | Christine Bergeron, RN |  |  |
|  | Gilbert Gosselin, MD | Christine Shelley |  |  |
|  |  | Christine Masson |  |  |
|  | Pallav Garg, MBBS, MSc | Sandy Carr, RN | London, ON | London Health Sciences Centre (35) |
|  |  | Catherine Bone, RN |  |  |
|  | Benjamin J.W. Chow, MD | Ermina Moga | Ottawa, ON | University of Ottawa Heart Institute (29) |
|  | Renee C. Hessian, MD | Janetta Kourzenkova |  |  |
|  | Rob S. Beanlands, MD | Olga Walter |  |  |
|  | Richard F. Davies, MD |  |  |  |
|  | Kevin R. Bainey, MD, MSc | Norma Hogg, RN | Edmonton, AB | University of Alberta (28) |
|  |  | Suzanne Welsh, RN |  |  |
|  | Asim N. Cheema, MD, PhD |  | Toronto, ON | St. Michael's Hospital (27) |
|  | Akshay Bagai, MD, MHS |  |  |  |
|  | Ron Wald, MDCM, MPH |  |  |  |
|  | Shaun Goodman, MD, MSc | Khrystyna Kushniriuk, HBSc, MD |  |  |
|  | John Joseph Graham, MRCP, MB ChB, BSc | Mohammed Hussain |  |  |
|  | Mark Peterson, MD, FRCSC, PhD | Olugbenga Bello |  |  |
|  | Chi-Ming Chow, MD, CM, MSc |  |  |  |
|  | Beth Abramson, MD, MSc |  |  |  |
|  | Asim Nazir Cheema, MD | Ishba Syed, MBBS | Mississauga, ON | Dixie Medical Group (24) |
|  | Mohammad Tariq Vakani, MD | Mohammed Hussain, BSc(H) |  |  |
|  |  | Khrystyna Kushniriuk, MBBS |  |  |
|  | James Cha, MD | Judy Otis, CRC | Oshawa, ON | Dr. James Cha (21) |
|  |  | Rebecca Otis, CRC |  |  |
|  | Andrew G Howarth, MD, PhD | Michelle M Seib, RN | Calgary, AB | University of Calgary (15) |
|  |  | Sandra M Rivest, RN |  |  |
|  |  | Rosa Sandonato, BSCN |  |  |
|  | Graham Wong, MD | Jackie Chow | Vancouver, BC | Vancouver General Hospital (15) |
|  |  | Andrew Starovoytov |  |  |
|  |  | Naomi Uchida |  |  |
|  |  | Ngaire Meadows |  |  |
|  | Amar Uxa, MD | Nadia Asif | Toronto, ON | University Health Network (14) |
|  |  | Suzana Tavares |  |  |
|  | Paul Galiwango, MD | Bev Bozek, RN, CCRC | Scarborough, ON | Scarborough Cardiology Research (9) |
|  | Saleem Kassam, MD | Maria Shier |  |  |
|  | Ashok Mukherjee, MD | Lori-Ann Larmand |  |  |
|  | A. Joseph Ricci, MD | Amir Janmohamed |  |  |
|  |  | Brenda Hart |  |  |
|  | Andy Lam, MD | Jane Marucci | East Grimsby, ON | West Lincoln Memorial Hospital (8) |
|  |  | Sharon Tai |  |  |
|  | Shamir Mehta, MD | Sonya Brons, RN | Hamilton, ON | Hamilton General Hospital (7) |
|  |  | Chris Beck, RN |  |  |
|  |  | Glenda Wong, RN |  |  |
|  |  | Krystal Etherington |  |  |
|  |  | Thippeekaa Arumairajah |  |  |
|  | Jacob Udell, MD | Maria Aprile | Toronto, ON | Women's College Hospital (7) |
|  |  | Sara Karlsson |  |  |
|  |  | Susan Webber |  |  |
|  | Philippe Généreux, MD | Chantale Mercure | Montréal, QC | Centre Intégré Universitaire de Santé et de Services Sociaux du Montréal (2) |
|  | Adnan Hameed, MD | Nancy Aedy | St. Catharines, ON | Saint Catharines General Hospital (2) |
|  |  |  |  |  |
|  | Ledjalem Daba, MD | Fran Farquharson | Vaughan, ON | Northwest GTA Cardiovascular and Heart Rhythm Program (1) |
|  |  | Anam Siddiqui |  |  |
| Brazil (399) |  |  |  |  |
| Country Leaders |  |  |  |  |
| Antonio Carlos Carvalho, MD, PhD | |  |  |  |
| Renato D. Lopes, MD, PhD |  |  |  |  |
|  | Whady Hueb, MD | Myrthes Emy Takiuti, RN | Sao Paulo | Heart Institute (InCor) University of São Paulo (127) |
|  | Paulo Cury Rezende, MD |  |  |  |
|  | Expedito Eustáquio Ribeiro Silva, MD |  |  |  |
|  | Alexandre Ciappina Hueb, MD |  |  |  |
|  | Paola Emanuela Poggio Smanio, MD, PhD | Leonardo Pizzol Caetano, PhD | São Paulo | Instituto Dante Pazzanese de Cardiologia (98) |
|  | Alexandre Schaan de Quadros, MD |  | Porto Alegre | Instituto de Cardiologia de Porto Alegre (41) |
|  | Renato Abdala Karam Kalil, MD | Aline Peixoto Deiro |  |  |
|  | José Luiz da Costa Vieira, MD | Alice Manica Muller |  |  |
|  | Gabriel Grossmann , MD | Maria Antonieta Pereira de Moraes |  |  |
|  | Pedro Píccaro de Oliveira, MD | Bruna Maria Ascoli |  |  |
|  | Leonardo Bridi, MD | Sílvia Zottis Poletti |  |  |
|  | Simone Savaris, MD |  |  |  |
|  | João V Vitola, MD, PhD |  | Curitiba | Quanta Diagnostico & Terapia (33) |
|  | Rodrigo J Cerci, MD, Msc | Sandra S. Zier, BSc |  |  |
|  | Fabio R Farias, MD, Msc | Vilmar Veiga Jr, BSc |  |  |
|  | Miguel M Fernandes, MD, PhD |  |  |  |
|  | José Antonio Marin-Neto, MD, PhD |  | Ribeirao Preto | Hospital das Clinicas da Faculdade de Medicina de Ribeirão Preto da Universidade de São Paulo (31) |
|  | André Schmidt, MD, PhD |  |  |  |
|  | Moysés de Oliveira Lima Filho, MD, PhD | Diego Franca da Cunha |  |  |
|  | Ricardo Mendes Oliveira, MD |  |  |  |
|  | João Reynaldo Abbud Chierice, MD |  |  |  |
|  | Carísi A. Polanczyk, MD | Guilherme G Rucatti, PsyD | Porto Alegre | Hospital de Clínicas de Porto Alegre |
|  | Mariana V. Furtado, MD | Fernanda Igansi, BSc |  | -12 |
|  | Luis F. Smidt, MD | Mauren P Haeffner, BSc |  |  |
|  | Antonio Carlos Carvalho, MD | Viviane Almeida | Sao Paulo | Unifesp - Hospital Sao Paulo (9) |
|  | Gustavo Pucci, MD | Gabriela Sanchez de Souza |  |  |
|  | Flavio Lyra, MD |  |  |  |
|  | Alvaro Rabelo Alves Junior, MD | Mayana Almeida | Salvador | Fundacao Bahiana de Cardilogia (9) |
|  |  | Viviane dos Santos |  |  |
|  | Marianna D. A. Dracoulakis, MD, PhD | Natalia S Oliveira, RN | Salvador | Hospital da Bahia (8) |
|  | Rodolfo G. S. D Lima, MD |  |  |  |
|  | Estevao Figueiredo, MD | Bruna Edilena Paulino Azevedo | Belo Horizonte | Hospital Lifecenter (8) |
|  | Paulo Ricardo Caramori, MD | Marco Bizzaro Santos | Porto Alegre | Hospital Sao Lucas da Pontificia Universidade Catolica do Rio Grande do Sol (7) |
|  |  | Amanda Germann |  |  |
|  |  | Vitor Gomes |  |  |
|  |  | Rosa Homem |  |  |
|  |  | Ellen Magedanz |  |  |
|  | Rogerio Tumelero, MD | Rosane Laimer | Fundo | Hospital Sao Vicente de Paulo (5) |
|  |  | Alexandre Tognon |  |  |
|  | Frederico Dall’Orto, MD |  | Pocos de Caldas | Hospital Maternidade e Pronto Socorro Santa Lucia (4) |
|  | Claudio T. Mesquita, MD | Roberta P Santos, RN | Botafogo | Hospital Pró-Cardíaco (3) |
|  | Alexandre S. Colafranseschi, MD |  |  |  |
|  |  |  |  |  |
|  | Amarino C. Oliveira Jr., MD |  |  |  |
|  | Luiz A. Carvalho, MD |  |  |  |
|  | Isabella C. Palazzo, MD |  |  |  |
|  | Andre S. Sousa, MD |  |  |  |
|  | Expedito Eustáquio Ribeiro da Silva, MD, PhD | | Sao Paulo | Hospital TotalCor (2) |
|  | Pedro Gabriel Melo de Barros e Silva, MD, PhD | Mariana Yumi Okada, RN |  |  |
|  | Luciana de Pádua Silva Baptista, MD, PhD | Ana Paula Batista, RN |  |  |
|  | Marcelo Jamus Rodrigues, MD | Aline Nogueira Rabaça, BS |  |  |
|  | Marcos Valério Coimbra de Resende, MD, PhD | |  |  |
|  | Jose Francisco Saraiva, MD | Larissa Miranda Trama | Sao Paulo | Hospital Celso Pierro (1) |
|  |  | Talita Silva |  |  |
|  |  | Camila Thais de Souza Ormundo |  |  |
|  |  | Carla Vicente |  |  |
|  | Costantino Costantini, MD, PhD | Caroline Pinheiro | Curitiba | Hospital Cardiologico Costantini (1) |
|  |  | Daniele Komar |  |  |
| Poland (333) |  |  |  |  |
| Country Leaders |  |  |  |  |
| Witold Ruzyllo, MD |  |  |  |  |
| Hanna Szwed, MD, PhD |  |  |  |  |
| Country Coordinator |  |  |  |  |
| Radoslaw Pracon, MD, PhD |  |  |  |  |
|  | Marcin Demkow, MD, PhD |  | Warsaw | Coronary and Structural Heart Diseases Department, Institute of Cardiology (127) |
|  | Radoslaw Pracon, MD, PhD |  |  |  |
|  | Cezary Kepka, MD PhD |  |  |  |
|  | Anna Teresinska, MD PhD | Olga Walesiak |  |  |
|  | Karolina Kryczka, MD PhD | Katarzyna Malinowska |  |  |
|  | Jan Henzel, MD PhD |  |  |  |
|  | Mateusz Solecki, MD PhD |  |  |  |
|  | Edyta Kaczmarska, MD PhD |  |  |  |
|  | Tomasz Mazurek, MD, PhD | Jakub Maksym, MD | Warszawa | Medical University of Warsaw (48) |
|  |  | Karolina Wojtera, MD |  |  |
|  |  | Anna Fojt, MD |  |  |
|  |  | Ewa Szczerba, MD |  |  |
|  | Jaroslaw Drozdz, PhD |  | Lodz | Cardiology Clinic, Medical University in Lodz (43) |
|  | Bartosz Czarniak, MD |  |  |  |
|  | Malgorzata Frach (formerly Stasiak), MD |  |  |  |
|  | Konrad Szymczyk, MD |  |  |  |
|  | Iwona Niedzwiecka, MD |  |  |  |
|  | Sebastian Sobczak, MD |  |  |  |
|  | Tomasz Ciurus, MD |  |  |  |
|  | Piotr Jakubowski, MD |  |  |  |
|  | Magdalena Misztal-Teodorczyk, MD |  |  |  |
|  | Dawid Teodorczyk, MD | Marta Swiderek, MA |  |  |
|  | Aleksandra Fratczak, MD | Ewelina Wojtala, MA |  |  |
|  | Marcin Szkopiak, MD |  |  |  |
|  | Patrycja Lebioda, MD |  |  |  |
|  | Michal Wlodarczyk, MD |  |  |  |
|  | Anna Plachcinska, MD |  |  |  |
|  | Jacek Kusmierek, MD |  |  |  |
|  | Magdalena Miller, MD |  |  |  |
|  | Halina Marciniak, MD |  |  |  |
|  | Karolina Wojtczak-Soska, MD |  |  |  |
|  | Katarzyna Łuczak, MD |  |  |  |
|  | Tomasz Tarchalski, MD |  |  |  |
|  | Anna Cichocka-Radwan, MD |  |  |  |
|  | Hanna Szwed, MD, PhD | Jaroslaw Karwowski, MD | Warsaw | National Institute of Cardiology, Warsaw (35) |
|  | Grazyna Anna Szulczyk, MD |  |  |  |
|  | Adam Witkowski, MD, PhD |  | Warsaw | Department of Interventional Cardiology & Angiology, Institute of Cardiology (20) |
|  | Krzysztof Kukuła, MD, PhD |  |  |  |
|  | Małgorzta Celińska-Spodar, MD |  |  |  |
|  | Joanna Zalewska, MD |  |  |  |
|  | Grzegorz Gajos, MD, PhD |  | Krakow | Department of Coronary Disease, John Paul II Hospital, Jagiellonian University Medical College  (16) |
|  | Krzysztof Bury, MD, PhD |  |  |  |
|  | Piotr Pruszczyk, MD, PhD | Andrzej Łabyk, MD | Warszawa | Department of Internal Medicine and Cardiology, Infant Jesus Teaching Hospital, Medical University of Warsaw (15) |
|  | Marek Roik, MD, PhD | Agnieszka Szramowska, MD |  |  |
|  |  | Olga Zdończyk, MD |  |  |
|  | Krystyna Łoboz-Grudzień, MD, PhD | Joanna Jaroch, MD, PhD | Wrocław | T.Marciniak Hospital (11) |
|  | Leszek Sokalski, MD, PhD |  |  |  |
|  | Barbara Brzezińska, MD, PhD |  |  |  |
|  | Maciej Lesiak, Professor, MD |  | Poznan | Szpital Kliniczny Przemienienia Pańskiego (10) |
|  | Magdalena Łanocha, MD |  |  |  |
|  | Krzysztof W. Reczuch, MD | Adam Kolodziej, MD | Wroclaw | Military Hospital / Medical University (4) |
|  | Zbigniew Kalarus, MD |  | Zabrze | Medical University of Silesia, School of Medicine with the Division of Dentistry, Department of Cardiology, Congenital Heart Diseases and Electrotherapy, Silesian Center for Heart Diseases (3) |
|  | Andrzej Swiatkowski, MD |  |  |  |
|  | Mariola Szulik, MD |  |  |  |
|  | Wlodzimierz J. Musial, MD | Marta Marcinkiewicz-Siemion, MD | Bialystok | University Hospital in Bialystok (1) |
| Russia (303) |  |  |  |  |
| Country Coordinator |  |  |  |  |
| Olga Bockeria, MD, PhD |  |  |  |  |
|  | Leo Bockeria, MD, PhD | Olga Bockeria, MD, PhD | Moscow | National Medical Research Center for Cardiovascuar Surgery (113) |
|  | Karen Petrosyan, MD, PhD | Zalina Kudzoeva, MD |  |  |
|  | Tatiana Trifonova, MD | Nodira Aripova, MD |  |  |
|  | Alexander M. Chernyavskiy, MD, PhD | Ivan A. Naryshkin, MD | Novosibirsk | E.Meshalkin National Medical Research Center of the Ministry of Health of the Russian Federation (101) |
|  | Evgeniy I. Kretov, MD | Alena Kuleshova, MD |  |  |
|  | Igor O. Grazhdankin, MD | Dastan Malaev, MD |  |  |
|  | Leonid L. Bershtein, MD, PhD |  | Saint Petersburg | North-Western State Medical University (50) |
|  | Sergey A. Sayganov, MD, PhD | Irina Subbotina |  |  |
|  | Anastasia M. Kuzmina-Krutetskaya, MD | Victoria Gumerova |  |  |
|  | Elizaveta V. Zbyshevskaya, MD, PhD |  |  |  |
|  | Nana O. Katamadze, MD, PhD |  |  |  |
|  | Elena A. Demchenko, MD, PhD | Olga B. Nikolaeva, MD | Saint Petersburg | Federal Almazov North-West Medical Research Centre (39) |
|  | Pavel S. Kozlov, MD |  |  |  |
|  | Vikentiy Y. Kozulin, MD |  |  |  |
|  | Ekaterina I. Lubinskaya, MD |  |  |  |
| *Spain (286) |  |  |  |  |
| Country Leader |  |  |  |  |
| Jose Luis Lopez-Sendon, MD, PhD | |  |  |  |
| Country Coordinator |  |  |  |  |
| Almudena Castro, MD |  |  |  |  |
|  | Jose Lopez-Sendon, MD, PhD | Virginia Fernández-Figares, Pharm | Madrid | Hospital La Paz. IdiPaz (118) |
|  | Almudena Castro, MD |  |  |  |
|  | Elena Refoyo Salicio, MD |  |  |  |
|  | Gabriela Guzman, MD |  |  |  |
|  | Gabriel Galeote, MD |  |  |  |
|  | Silvia Valbuena, MD |  |  |  |
|  | Jesús Peteiro, MD, PhD |  | A Coruna | Complexo Hospitalario Universitario A Coruña (CHUAC) Sergas, Department of Cardiology. INIBIC A Coruña. CIBER-CV. Universidad de A Coruña, Spain (112) |
|  | María Dolores Martínez-Ruíz, MD |  |  |  |
|  | Ruth Pérez-Fernández, MD | Moisés Blanco-Calvo, PhD |  |  |
|  | José J Cuenca-Castillo, MD | Encarnación Alonso-Álvarez, BSc |  |  |
|  | Xacobe Flores-Ríos, MD | Paula García-González, BSc |  |  |
|  | Óscar Prada-Delgado, MD |  |  |  |
|  | Gonzalo Barge-Caballero, MD |  |  |  |
|  | Jose Ramon Gonzalez Juanatey, MD, PhD | Jose Seijas Amigo, Pharm | Santiago de Compostela | Hospital Clinico Universitario de Santiago (17) |
|  | Miguel Souto Bayarri, MD, PhD |  |  |  |
|  | Virginia Pubull Nuñez, MD |  |  |  |
|  | Raymundo Ocaranza Sanchez, MD, PhD |  |  |  |
|  | Belen Cid Alvarez, MD |  |  |  |
|  | Carlos Peña Gil, MD, PhD |  |  |  |
|  | Amparo Martinez Monzonis, MD |  |  |  |
|  | Alessandro Sionis, MD | Ana Fernández Martínez, RN | Barcelona | Hospital de la Santa Creu i Sant Pau (11) |
|  | Montserrat Vila Perales, MD |  |  |  |
|  | Josep Maria Padró, MD |  |  |  |
|  | Antonio Serra Peñaranda, MD |  |  |  |
|  | Joan García Picart, MD |  |  |  |
|  | Antonino Ginel Iglesias, MD |  |  |  |
|  | Xavier Garcia-Moll Marimon, MD |  |  |  |
|  | Guillem Pons Lladó, MD |  |  |  |
|  | Francesc Carreras Costa, MD |  |  |  |
|  | Vicente Miro, MD | Begoña Igual, MD | Valencia | Hospital Universitario y Politecnico La Fe (10) |
|  | Jose L Diez, MD |  |  |  |
|  | Pilar Calvillo, MD |  |  |  |
|  | F. Marin Ortuño, MD, PhD |  | Murcia | HUVA, Hospital Clínico Universitario Virgen De La Arrixaca (8) |
|  | M. Valdés Chávarri, MD, PhD | M. Quintana Giner, MD |  |  |
|  | A. Tello Montolliu, MD, PhD | A.I. Romero Aniorte, MD |  |  |
|  | E. Pinar Bermudez, MD, PhD | JM. Rivera Caravaca, MD |  |  |
|  | G. De La Morena, MD, PhD |  |  |  |
|  | Montserrat Gracida Blancas, MD | Olga Cañavate | Barcelona | Hospital De Bellvitge (4) |
|  |  | Sonia Guerrero |  |  |
|  |  | Silvia Riera |  |  |
|  | Jose Enrique Castillo Luena, MD | Jose Enrique Castillo Luena | Zaragoza | Hospital Universitario Miguel Servet (4) |
|  |  | Maria Lasala |  |  |
|  | Francisco Fernandez-Aviles, MD | Maria Lorenzo | Madrid | Hospital General Universitario Gregorio Maranon (2) |
|  |  | Olga Sobrino |  |  |
|  |  | Alexandra Vazquez |  |  |
| China (246) |  |  |  |  |
| Country Leader |  |  |  |  |
| Lixin Jiang, MD, PhD |  |  |  |  |
|  | Jiyan Chen, MD | Haojian Dong | Guangzhou | Guangdong General Hospital (102) |
|  |  | Peiyu He |  |  |
|  |  | Chunli Xia |  |  |
|  |  | Junqing Yang |  |  |
|  |  | Qi Zhong |  |  |
|  | Yongjian Wu, MD, PhD | Yanmeng Tian, MD | Beijing | Chinese Academy of Medical Sciences, Fuwai Hospital (17) |
|  |  | Dongze Li | Urumqi | First Affiliated Hospital of Xinjiang Medical University (15) |
|  | Yitong Ma, MD | Xiaomei Li |  |  |
|  | Yining Yang, MD | Xiang Ma |  |  |
|  |  | Zixiang Yu |  |  |
|  |  | Qian Zhao |  |  |
|  | Zheng Ji, MD | Chunguang Li | Tangshan | Tangshan Gongren Hospital (15) |
|  |  | Lei Zhang |  |  |
|  |  | Yu Zhao |  |  |
|  |  | Bolin Zhu |  |  |
|  | Xinchun Yang, MD | Mulei Chen | Beijing | Beijing Chao-yang Hospital, Capital Medical University (12) |
|  |  | Hongjie Chi |  |  |
|  |  | Yang Wang |  |  |
|  |  | Jing Zhang |  |  |
|  | Wenhua Lin, MD | Rui Jing | Tianjing | TEDA International Cardiovascular Hospital (12) |
|  |  | Jingjing Liu |  |  |
|  | Hesong Zeng, MD | Qiang Zhou, MD | Wuhan | Tongji Medical College (11) |
|  |  | Chang Xu, MD |  |  |
|  |  | Zhuxi Li, MD |  |  |
|  |  | Junhua Li, MD |  |  |
|  |  | Luyang Xiong, MD |  |  |
|  | Xin Fu, MD | Dan Gao | Zhengzhou | The First Affiliated Hospital of Zhengzhou University (11) |
|  |  | Dengke Jiang |  |  |
|  |  | Ran Leng |  |  |
|  |  | Xutong Wang |  |  |
|  |  | Qianqian Yuan |  |  |
|  |  | Lili Zhang |  |  |
|  | Bin Yang, MD | Ziliang Bai | Taiyuan | Shanxi Cardiovascular Hospital (10) |
|  |  | Jianhua Li |  |  |
|  |  | Jie Qi |  |  |
|  |  | Fei Wang |  |  |
|  |  | Haitao Wang |  |  |
|  |  | Bin Yang |  |  |
|  |  | Zhou Yue |  |  |
|  |  | Zhulin Zhang |  |  |
|  | Songtao Wang, MD | Yumei Dong | Qingdao | Qingdao Fuwai Hospital (8) |
|  |  | Jiajia Mao |  |  |
|  |  | Bin Zhang |  |  |
|  | Gong Cheng, MD | Xiuhong Li | Xian | Shanxi Provincial People’s Hospital (6) |
|  |  | Xiaowei Yao |  |  |
|  |  | Nier Zhong |  |  |
|  |  | Ning Zhou |  |  |
|  | Yulan Zhao, MD | Yaping Huang, MS | Zhengzhou | The Second Affiliated Hospital of Zhengzhou University (6) |
|  |  | Panpan Zhou, MS |  |  |
|  | Xuehua Fang, MD | Wei Su | Beijing | Liangxiang Hospital, Beijing Fangshan District (6) |
|  | Qiutang Zeng, MD | Yu Kunwu | Wuhan | Wuhan Union Hospital, Tongji Medical College, Huazhong Science and Tech University (3) |
|  |  | Yudong Peng |  |  |
|  |  | Xin Su |  |  |
|  | Xi Su, MD | Chen Wang | Wuhan | Wuhan Asia Heart Hospital (3) |
|  |  | Yunhai Zhao |  |  |
|  | Qingxian Li, MD | Yaming Geng | Jining | Affiliated Hospital of Jining Medical University (3) |
|  |  | Yanfu Wang |  |  |
|  | Shao-ping Nie, MD, PhD | Jing-yao Fan, MD | Beijing | Beijing Anzhen Hospital (2) |
|  |  | Si-ting Feng, MD,PhD |  |  |
|  |  | Xiao Wang, MD,PhD |  |  |
|  |  | Yan Yan, MD,PhD |  |  |
|  |  | Hui-min Zhang, MD,PhD |  |  |
|  | Qin Yu, MD | Lingping Chi | Dalian | Affiliated Zhongshan Hospital of Dalian University (2) |
|  |  | Fang Liu |  |  |
|  | Jian'an Wang, MD | Han Chen | Hangzhou | The Second Affiliated Hospital Zhejiang University School of Medicine (1) |
|  |  | Jun Jiang |  |  |
|  |  | Huajun Li |  |  |
|  |  | Jian'an Wang |  |  |
|  |  | Yechen Han, MM | Beijing | Peking Union Medical College Hospital (1) |
|  |  | Lihong Xu, RN |  |  |
|  | Shuyang Zhang, MD, PhD | Zhenyu Liu |  |  |
|  | Zhenyu Liu, MD | Gang Chen |  |  |
|  |  | Rongrong Hu |  |  |
| *Italy (139) |  |  |  |  |
| Country Leader |  |  |  |  |
| Aldo P. Maggioni, MD |  |  |  |  |
|  |  |  |  |  |
|  | Gian Piero Perna, MD | Francesca Pietrucci, PhD | Ancona | Cardiology and CCU - Ospedali Riuniti Ancona (54) |
|  | Marco Marini, MD |  |  |  |
|  | Gabriele Gabrielli, MD |  |  |  |
|  | Stefano Provasoli, MD | Anna Di Donato | Varese | Ospedale di Circolo e Fondazione Macchi (23) |
|  | Edoardo Verna, MD |  |  |  |
|  | Lorenzo Monti, MD |  | Rozzano | Humanitas Research Hospital, Rozzano (MI) (17) |
|  | Barbara Nardi, MD |  |  |  |
|  | Antonio Di Chiara, MD | Francesca Pezzetta, MD | Tolmezzo | Azienda Servizi Sanitaria n.3 Alto Friuli-Collinare-Medio Friuli (9) |
|  | Andrea Mortara, MD | Valentina Casali, MD | Monza | Policlinico di Monza, Monza MB (8) |
|  | Marcello Galvani, MD | Chiara Attanasio | Forli | Ospedale “G.B. Morgagni – L. Pierantoni” Forli (AUSL della Romagna) (8) |
|  | Filippo Ottani, MD |  |  |  |
|  | Marco Sicuro, MD | Gianpiero Leone, MD | Aosta | Ospedale Regionale Umberto Parini (5) |
|  |  | Francesco Pisano, MD |  |  |
|  |  | Cristina Bare, BSc |  |  |
|  | Paolo Calabro, MD | Fabio Fimiani | Napoli | AORN Dei Colli "V. Monaldi" UOC Cardiologia Università della Campania "L.Vanvitelli" (4) |
|  | Tiziana Formisano, MD |  |  |  |
|  | Giuseppe Tarantini, MD | Alberto Barioli, MD | Padua | University of Padua- Cardiology Clinic (3) |
|  | Umberto Cucchini, MD | Federica Ramani |  |  |
|  | Anto Luigi Andres, MD |  |  |  |
|  | Emanuela Racca, MD | Fabrizio Rolfo, MD | Cuneo | Azienda Ospedaliera S. Croce e Carle (3) |
|  |  | Cecilia Goletto |  |  |
|  | Carlo Briguori, MD | Francesca De Micco | Naples | Clinica Mediterranea (2) |
|  | Roberto Amati, MD | Stefano Di Marco, MD | Pescia | UO Cardiologia Ospedale SS Cosma e Damiano (2) |
|  | William Vergoni, MD | Martina Tricoli |  |  |
|  | Aldo Russo, MD | Massimo Villella, MD | San Giovanni Rotondo | IRCCS "Casa Sollievo della Sofferenza" (1) |
|  | Raffaele Fanelli, MD |  |  |  |
| *Singapore (61) |  |  |  |  |
| Country Leader |  |  |  |  |
| Harvey Douglas White, MD |  |  |  |  |
| Country Coordinator |  |  |  |  |
| Caroline Alsweiler |  |  |  |  |
|  | Kian-Keong Poh, MD |  | Singapore | National University Heart Center Singapore (33) |
|  | Ping Chai, MD |  |  |  |
|  | Titus Lau, MD |  |  |  |
|  | Joshua P. Loh, MD |  |  |  |
|  | Edgar L. Tay, MD |  |  |  |
|  | Kristine Teoh, MD | Sik-Yin V Tan, BSc |  |  |
|  | Lynette L. Teo, MD | Winnie C Sia, BSc |  |  |
|  | Ching-Ching Ong, MD | Audrey W Leong, BSc |  |  |
|  | Raymond C. Wong, MD |  |  |  |
|  | Poay-Huan Loh, MD |  |  |  |
|  | Theodoros Kofidis, MD |  |  |  |
|  | Wan Xian Chan, MD |  |  |  |
|  | Koo Hui Chan, MD |  |  |  |
|  | David Foo, MBBS | Li Hai Yan, RN | Singapore | Tan Tock Seng Hospital (22) |
|  | Jason Loh Kwok Kong, MD |  |  |  |
|  | Ching Min Er, MD |  |  |  |
|  | Fahim Haider Jafary, MD |  |  |  |
|  | Terrance Chua, MD | Nasrul Ismail | Singapore | National Heart Centre Singapore (6) |
|  |  | Min Tun Kyaw |  |  |
|  |  | Deborah Yip |  |  |
| Germany (54) |  |  |  |  |
| Country Leader |  |  |  |  |
| Rolf Doerr, MD |  |  |  |  |
|  | Rolf Doerr, MD |  | Dresden | Praxisklinik Herz und Gefaesse (29) |
|  | Juergen Stumpf, MD | Dorit Grahl |  |  |
|  | Klaus Matschke, MD, PhD | Franziska Guenther |  |  |
|  | Gregor Simonis, MD, PhD | Kerstin Bonin |  |  |
|  | Clemens T. Kadalie, MD |  |  |  |
|  | Udo Sechtem, MD | Ina Wenzelburger | Stuttgart | Robert-Bosch-Krankenhaus (22) |
|  | Peter Ong, MD | Susanne Gruensfelder, RN |  |  |
|  | P. Christian Schulze, MD, PhD |  | Jena | University Hospital Jena (2) |
|  | Bjoern Goebel, MD |  |  |  |
|  | Karsten Lenk, MD |  |  |  |
|  | Georg Nickenig, MD | Jan-Malte Sinning, MD | Bonn | Universitatsklinikum Bonn (1) |
|  |  | Marcel Weber, MD |  |  |
|  |  | Nikos Werner, MD |  |  |
| Austria (50) |  |  |  |  |
| Country Leaders |  |  |  |  |
| Irene Marthe Lang, MD |  |  |  |  |
| Kurt Huber, MD |  |  |  |  |
|  | Herwig Schuchlenz, MD | Gudrun Steinmaurer | Graz | LKH Graz West Austria (35) |
|  | Stefan Weikl, MD |  |  |  |
|  | Irene Marthe Lang, MD | Max-Paul Winter, MD | Vienna | Medical University of Vienna, Department of Cardiology (8) |
|  |  | Tijana, Andric, MD | Vienna | Wilhelminen Hospital Vienna (7) |
|  | Kurt Huber, MD | Maximilian, Tscharre, MD |  |  |
|  | Gabriele, Jakl-Kotauschek, MD | Claudia, Wegmayr, MSc |  |  |
|  |  | Bernhard, Jäger, MD |  |  |
|  |  | Florian, Egger, MD |  |  |
| Hungary (49) |  |  |  |  |
| Country Leader |  |  |  |  |
| Matyas Keltai, MD, PhD, DSc |  |  |  |  |
|  | Andras Vertes, MD | Judit Sebo, MD | Budapest | Eszszk- Szent Istvan Hospital (20) |
|  |  | Zoltan Davidovits, MD |  |  |
|  |  | Laszlone Matics |  |  |
|  | Albert Varga, MD, PhD | Gergely Ágoston, MD | Szeged | University of Szeged (12) |
|  | Geza Fontos, MD | Gabor Dekany, MD | Budapest | George Gottsegen National Institute of Cardiology (9) |
|  | Bela Merkely, MD, PhD, DSc | Andrea Bartykowszki, MD | Budapest | Heart and Vascular Center, Semmelweis University (8) |
|  |  | Pal Maurovich-Horvat, MD, PhD, MPH | |  |
|  | Gabor Kerecsen, MD | Agnes Jakal | Budapest | Military Hospital, Budapest (1) |
| Serbia (47) |  |  |  |  |
|  | Sasa Hinic, MD, BSc | Jelena Djokic, MD | Belgrade | University Hospital Center Bezanijska Kosa (13) |
|  | Marija Zdravkovic, MD, PhD |  |  |  |
|  | Vladan Mudrenovic, MD |  |  |  |
|  | Bogdan Crnokrak, MD |  |  |  |
|  | Branko D. Beleslin, MD, PhD |  | Belgrade | Faculty of Medicine, University of Belgrade; Cardiology Clinic, Clinical Center of Serbia (10) |
|  | Nikola N. Boskovic, MD | Ana D. Djordjevic-Dikic, MD, PhD |  |  |
|  | Marija T. Petrovic, MD | Vojislav L. Giga, MD, PhD |  |  |
|  | Milan R. Dobric, MD | Jelena J. Stepanovic, MD, PhD |  |  |
|  | Zeljko Z. Markovic, MD, PhD |  |  |  |
|  | Ana S. Mladenovic, MD, PhD |  |  |  |
|  | Nada Cemerlic-Adjic, MD | Lazar Velicki, MD | Sremska Kamenica | Institute of Cardiovascular Diseases Vojvodina, Sremska Kamenica, Serbia and Faculty of Medicine, University of Novi Sad (9) |
|  |  | Ljiljana Pupic |  |  |
|  | Goran Davidović, MD, PhD | Stefan M. Simović, MD | Kragujevac | Clinical Center Kragujevac (7) |
|  | Rada Vučić, MD |  |  |  |
|  | Milica Nikola Dekleva, MD PhD | Miroslav Stevo Martinovic, MD | Belgrade | University Clinical Hospital Zvezdara (6) |
|  |  | Gordana Stevanovic |  |  |
|  | Goran Stankovic, MD | Milan Dobric | Belgrade | Clinical Center of Serbia (1) |
|  | Svetlana Apostolovic, MD | Sonja Salinger Martinovic | Nis | Clinic for Cardiovascular Diseases, Clinical Center Nis (1) |
|  |  | Dragana Stanojevic |  |  |
| Mexico (46) |  |  |  |  |
|  | Jorge Escobedo, MD | Ramon de Jesús-Pérez, RN | Benito Juarez | Instituto Mexicano del Seguro Social (35) |
|  | Rubén Baleón-Espinosa, MD |  |  |  |
|  | Arturo S Campos-Santaolalla, MD |  |  |  |
|  | Elihú Durán-Cortés, MD |  |  |  |
|  | José M Flores-Palacios, MD |  |  |  |
|  | Andrés García-Rincón, MD |  |  |  |
|  | Moisés Jiménez-Santos, MD |  |  |  |
|  | Joaquín V Peñafiel, MD |  |  |  |
|  | José A Ortega-Ramírez, MD |  |  |  |
|  | Aquiles Valdespino-Estrada, MD |  |  |  |
|  | Erick Alexánderson Rosas, MD | María Fernanda Canales Brassetti, MD | Mexico City | Instituto Nacional de Cardiología "Ignacio Chávez" (11) |
|  |  | Diego Adrián Vences Anaya, MD |  |  |
|  |  | María Pérez García |  |  |
|  |  | Isabel Estela Carvajal Juarez , MD |  |  |
|  |  | Magdalena Madero Rovalo, MC |  |  |
|  |  | Erick Donato Morales Rodríguez, MD | |  |
| Australia (45) |  |  |  |  |
| Country Leaders |  |  |  |  |
| Joseph B. Selvanayagam, MBBS (Hons), DPhil | |  |  |  |
| Jamie Rankin, MBBS *(past)* |  |  |  |  |
| Country Coordinator |  |  |  |  |
| Deirdre Murphy |  |  |  |  |
|  | Joseph B. Selvanayagam, MBBS (Hons), DPhil | Sau Lee, PhD | Adelaide | Flinders Medical Centre (30) |
|  | Majo X. Joseph, MBBS | Prince Thomas, RN |  |  |
|  | Suku T. Thambar, MBBS | Melissa D Chaplin, RN | New Lambton Heights | John Hunter Hospital (8) |
|  |  | Stephanie C Boer, B Biotechnology (Honours) | |  |
|  | John F. Beltrame, MD | Jeanette K. Stansborough, RN | Woodville South | The Queen Elizabeth Hospital (5) |
|  |  | Marilyn Black, RN |  |  |
|  | Graham S. Hillis, PhD | Michelle M. Bonner, B. Nursing | Perth | Royal Perth Hospital (2) |
|  |  | Kim F. Ireland, RN |  |  |
|  |  | Clare Venn-Edmonds, RN |  |  |
| France (42) |  |  |  |  |
| Country Leader |  |  |  |  |
| Philippe-Gabriel Steg, MD |  |  |  |  |
| Country Coordinators |  |  |  |  |
| Helene Abergel |  |  |  |  |
| Jean-Michel Juliard |  |  |  |  |
|  |  | Corine Thobois, RN | Chartres | C.H. Louis Pasteur (21) |
|  | Christophe Thuaire, MD | Emilie Tachot, RN |  |  |
|  | Téodora Dutoiu, MD | Christophe Laure, RN |  |  |
|  |  | Christel Vassaliere, RN |  |  |
|  | Philippe Gabriel Steg, MD | Helene Abergel, MSc | Paris | Bichat Hospital (9) |
|  | Jean-Michel Juliard, MD | Axelle Fuentes, MSc |  |  |
|  | Michel S. Slama, MD | Ludivine Eliahou, MD | Clamart Cedex | Antoine-Beclere Hospital (5) |
|  | Rami El Mahmoud, MD | Olivier Dubourg, MD | Boulogne | Ambroise Pare Hospital (2) |
|  |  | Pierre Michaud, MD |  |  |
|  | Eric Nicollet, MD | Sarah Hadjih | Corbeil-Essonnes Cedex | Centre Hospitalier Sud Francilien (2) |
|  | Pascal Goube, MD | Patricia Brito |  |  |
|  | Gilles Barone-Rochette, MD | Gilles Barone-Rochette | Grenoble | Grenoble University Hospital (2) |
|  | Alain Furber, MD | Charles Cornet, MD, PhD | Angers Cedex 9 | Centre Hospitalier Universitaire d'Angers (1) |
|  | Loïc Bière, MD | Jeremy Rautureau, MD, PhD |  |  |
| Lithuania (39) |  |  |  |  |
|  |  | Agne Juceviciene, MD | Vilnius | Vilnius University Hospital Santariskes Clinic (39) |
|  |  | Irma Kalibataite-Rutkauskiene, MD |  |  |
|  |  | Laura Keinaite |  |  |
|  | Aleksandras Laucevicius, MD | Monika Laukyte |  |  |
|  | Jelena Celutkiene, MD | Gelmina Mikolaitiene |  |  |
|  |  | Akvile Smigelskaite, MD |  |  |
|  |  | Ilona Tamasauskiene, MD |  |  |
|  |  | Agne Urboniene, MD |  |  |
| *Netherlands (37) |  |  |  |  |
|  | Elvin Kedhi MD, PhD |  | Zwolle | Isala Klinieken (25) |
|  | Jorik Timmer, MD | Ilse Bouwhuis |  |  |
|  | Rik Hermanides, MD | Lia Nijmeijer |  |  |
|  | Eliza Kaplan, MD |  |  |  |
|  | Robert K. Riezebos, MD, PhD |  | Amsterdam | Cardio Research Hartcentrum OLVG (11) |
|  | Pouneh Samadi, MD | Jeannette, J. M. Schoep, RN |  |  |
|  | Elise van Dongen, MD | Elisabeth, M. Janzen, RN |  |  |
|  | Sander R. Niehe, MD |  |  |  |
|  | Harry Suryapranata, MD | Sandra Ahoud | Nijmegen | Radboudumc (1) |
|  | Stijn van Vugt, MD, PhD |  |  |  |
| Portugal (33) |  |  |  |  |
|  | Ruben Ramos, MD |  | Lisbon | Hospital de Santa Marta (25) |
|  | Duarte Cacela, MD |  |  |  |
|  | Ana Santana, MD |  |  |  |
|  | Antonio Fiarresga, MD |  |  |  |
|  | Lidia Sousa, MD |  |  |  |
|  | Hugo Marques, MD |  |  |  |
|  | Lino Patricio, MD | Mafalda Selas |  |  |
|  | Luis Bernanrdes, MD | Filipa Silva |  |  |
|  | Pedro Rio, MD | Cláudia Freixo |  |  |
|  | Ramiro Carvalho, MD |  |  |  |
|  | Rui Ferreira, MD |  |  |  |
|  | Tiago Silva, MD |  |  |  |
|  | Ines Rodrigues, MD |  |  |  |
|  | Pedro Modas, MD |  |  |  |
|  | Guilherme Portugal, MD |  |  |  |
|  | Jose Fragata, MD |  |  |  |
|  | Fausto J. Pinto, PhD | Inês Zimbarra Cabrita, PhD | Lisbon | Santa Maria University Hospital, Cardiology Department, CHLN (6) |
|  | Miguel Nobre Menezes, MD | Andreia Rocha, MSc |  |  |
|  | Guilhermina Cantinho Lopes, MD | Francisca Patuleia Figueiras, PhD |  |  |
|  | Ana Gomes Almeida, PhD | Andreia Coelho, BSc |  |  |
|  | Pedro Canas Silva, MD | Marta Capinha |  |  |
|  | Angelo Nobre, MD | Maria Inês Caetano |  |  |
|  | Ana Rita Francisco, MD | Susana Silva |  |  |
|  | Nuno Ferreira, MD |  | Vila Nova de Gaia | Centro Hospitalar de Vila Nova de Gaia/Espinho, EPE (2) |
|  | Ricardo L. Lopes, MD |  |  |  |
| Argentina (29) |  |  |  |  |
| Country Leader |  |  |  |  |
| Rafael Diaz, MD *(past)* |  |  |  |  |
|  | Luis Guzman, MD | Veronica Tinnirello | Cordoba | Instituto Medico DAMIC (11) |
|  | Julio César Figal, MD | Matías Nicolás Mungo | Ciudad Autonoma de Buenos Aires | Fundación Favaloro (10) |
|  | Oscar Méndiz, MD |  |  |  |
|  | Claudia Cortés, MD |  |  |  |
|  | Roberto René Favaloro, MD |  |  |  |
|  | Carlos Alvarez, MD | Marina Garcia | Bahia Blanca | Hospital Italiano Regional del Sur Bahia Blanca (3) |
|  | Javier Courtis, MD | Valeria Godoy | Cordoba | Clinica Romagosa and Clinica De La Familia (2) |
|  | Gabriela Zeballos, MD |  |  |  |
|  | Lilia Schiavi, MD | Maria Victoria Actis | Cordoba | Clinica Del Prado (2) |
|  | Mariano Rubio, MD | Graciela Scaro, MD | Cordoba | Clínica Privada Vélez Sarsfield (1) |
| *New Zealand (28) |  |  |  |  |
| Country Leader |  |  |  |  |
| Harvey Douglas White, MD |  |  |  |  |
| Country Coordinator |  |  |  |  |
| Caroline Alsweiler |  |  |  |  |
|  | Gerard Patrick Devlin, MD | Liz Low, RN | Hamilton | Waikato Hospital (22) |
|  | Raewyn Fisher, MD | Jayne Scales, RN |  |  |
|  |  | Kirsty Abercrombie, RN |  |  |
|  | Ralph Alan Huston Stewart, MCChB, MD | Leah Howell , RN | Auckland | Auckland City Hospital (6) |
|  | Harvey Douglas White, MD | Cathrine Patten, RN |  |  |
|  | Jocelyne Benatar, MD |  |  |  |
| *Macedonia (28) |  |  |  |  |
|  | Sasko Kedev, MD, PhD |  | Skopje | University Clinic of Cardiology (28) |
|  | Irena Peovska Mitevska, MD, PhD |  |  |  |
|  | Elizabeta Srbinovska Kostovska, MD, PhD |  |  |  |
|  | Hristo Pejkov, MD, PhD |  |  |  |
| *Sweden (23) |  |  |  |  |
| Country Leader |  |  |  |  |
| Claes Held, MD, PhD |  |  |  |  |
|  | Claes Held, MD, PhD |  | Uppsala | Uppsala University (18) |
|  | Kai Eggers, MD, PHhD |  |  |  |
|  | Gunnar Frostfelt, MD, PhD | Christina Björklund, RN |  |  |
|  | Nina Johnston, MD, PhD | Maria Andreasson, RN |  |  |
|  | Maciej Olsowka, MD | Marie Essermark, RN |  |  |
|  | Axel Åkerblom, MD, PhD |  |  |  |
|  | Inga Soveri, MD, PhD |  |  |  |
|  | Johannes Aspberg, MD | Liselotte Persson | Stockholm | Karolinska Institutet at Danderyd Hospital (5) |
| Israel (15) |  |  |  |  |
| Country Leaders |  |  |  |  |
| Rafael Beyar, MD, MD, DSc, MPH | |  |  |  |
| Tali Sharir, MD |  |  |  |  |
| Country Coordinator |  |  |  |  |
| Eugenia Nikolsky, MD |  |  |  |  |
|  | Tali Sharir, MD | Or Harel, MA | Tel-Aviv | Assuta Medical Centers (9) |
|  | Dan Elian, MD |  |  |  |
|  | Arthur Kerner, MD | Margalit Bentzvi | Haifa | Rambam Medical Center (6) |
|  | Samia Massalha, MD | Ludmila Helmer |  |  |
| Japan (14) |  |  |  |  |
| Country Leader |  |  |  |  |
| Shun Kohsaka, MD |  |  |  |  |
|  | Keiichi Fukuda, MD, PhD | Ikuko Ueda, PhD | Shinjuku-ku | Keio University (7) |
|  | Shun Kohsaka, MD | Jun Fujita, MD |  |  |
|  | Satoshi Yasuda, MD, PhD | Akemi Furukawa, RN | Suita-shi | National Cerebral and Cardiovascular Center |
|  |  | Kanae Hirase, RN |  | -4 |
|  |  | Toshiyuki Nagai, MD, PhD |  |  |
|  |  | Fumiyuki Otsuka, MD, PhD |  |  |
|  | Shigeyuki Nishimura, MD | Shintaro Nakano | Hidaka | Saitama Medical University (3) |
| *Belgium (7) |  |  |  |  |
| Country Leader |  |  |  |  |
| Frans Van de Werf, MD, PhD |  |  |  |  |
| Country Coordinator |  |  |  |  |
| Kaatje Goetschalckx, MD |  |  |  |  |
|  | Kaatje Goetschalckx, MD | Valerie Robesyn | Leuven | University Hospital Leuven (7) |
|  | Frans Van de Werf, PhD, MD |  |  |  |
|  | Kathleen Claes, PhD, MD |  |  |  |
| *Taiwan (7) |  |  |  |  |
| Country Leader |  |  |  |  |
| Harvey Douglas White, MD |  |  |  |  |
| Country Coordinator |  |  |  |  |
| Caroline Alsweiler |  |  |  |  |
|  | Chung-Lieh Hung, MD | Yi-Hsuan Yang | Taipei City | Mackay Memorial Hospital (7) |
|  | Chun-Ho Yun, MD |  |  |  |
|  | Charles Jia-Yin Hou, MD |  |  |  |
|  | Jen-Yuan Kuo, MD |  |  |  |
|  | Hung-I Yeh, MD, PhD |  |  |  |
|  | Ta-Chuan Hung, MD |  |  |  |
|  | Jiun-Yi Li , MD, PhD |  |  |  |
|  | Chen-Yen Chien, MD, PhD |  |  |  |
|  | Cheng-Ting Tsai, MD |  |  |  |
|  | Chun-Chieh Liu, MD |  |  |  |
|  | Fa-Chang Yu, MD |  |  |  |
|  | Yueh-Hung Lin, MD |  |  |  |
|  | Wei-Ren Lan, MD |  |  |  |
|  | Chih-Hsuan Yen, MD |  |  |  |
|  | Jui-Peng Tsai, MD |  |  |  |
|  | Kuo-Tzu Sung, MD |  |  |  |
| *South Africa (7) |  |  |  |  |
|  | Mpiko Ntsekhe, MD |  | Cape Town | Groote Schuur Hospital / University of Cape Town (7) |
|  | Shaheen Pandie, MD | Constance Philander (Nee Talliard), ND | |  |
|  | Charle A Viljoen, MD | Noloyiso Mtana, RN |  |  |
|  | Marianne De Andrade, MD |  |  |  |
| *Switzerland (7) |  |  |  |  |
| Country Leader |  |  |  |  |
| Aldo P. Maggioni, MD |  |  |  |  |
|  | Tiziano Moccetti, MD | Adriana Anesini, RN | Lugano | Cardiocentro (7) |
|  | M.Grazia Rossi, MD | Simona Maspoli, RN |  |  |
|  |  | Manuela Mombelli, RN |  |  |
| Egypt (6) |  |  |  |  |
|  | Magdy Abdelhamid, MD | Ahmed Talaat, MD | Cairo | Cairo University (6) |
|  | Ahmed Adel, MD |  |  |  |
|  | Ahmed Kamal, MsC |  |  |  |
|  | Hossam Mahrous, MD |  |  |  |
|  | Sameh El Kaffas, MD |  |  |  |
|  | Hussien El Fishawy, MD |  |  |  |
| Romania (5) |  |  |  |  |
|  | Calin Pop, MD, PhD |  | Bucharest | Emergency County Hospital Baia Mare (4) |
|  | Matei Claudia, MD, PhD |  |  |  |
|  | Bogdan A. Popescu, MD, PhD |  | Bucharest | Emergency Institute of Cardiovascular Diseases ''Prof. Dr. C. C. Iliescu'' (1) |
|  | Carmen Ginghina, MD, PhD | Monica Rosca, MD, PhD |  |  |
|  | Dan Deleanu, MD, PhD | Carmen C. Beladan, MD, PhD |  |  |
|  | Vlad A. Iliescu, MD, PhD |  |  |  |
| *Saudi Arabia (5) |  |  |  |  |
|  | Mouaz H. Al-Mallah, MD MSc | Sarah Zahrani, RN | Central Province | King AbdulAziz Cardiac Center (5) |
|  | Ahmed Aljzeeri, MD |  |  |  |
|  | Hani Najm, MD |  |  |  |
|  | Ali Alghamdi, MD |  |  |  |
| *Peru (4) |  |  |  |  |
|  | Walter Enrique Mogrovejo Ramos, MD | Marco Antonio Monsalve Davila, RN | Mirafloes | Instituto Neuro Cardiovascular De Las Americas (4) |
| Thailand (3) |  |  |  |  |
| bCountry Leader |  |  |  |  |
| Harvey Douglas White, MD |  |  |  |  |
| Country Coordinator |  |  |  |  |
| Caroline Alsweiler |  |  |  |  |
|  | Srun Kuanprasert, MD |  | Chiang Mai | Maharaj Nakorn Chiang Mai Hospital (2) |
|  | Arintaya Prommintikul, MD |  |  |  |
|  | Weerachai Nawarawong, MD | Supatchara Khwakhong, RN |  |  |
|  | Surin Woragidpoonpol, MD | Anong Chaiyasri, RN |  |  |
|  | Thitipong Tepsuwan, MD | Warangkana Mekara, RN |  |  |
|  | Noppon Taksaudom, MD | Supap Kulthawong, RN |  |  |
|  | Chataroon Rimsukcharoenchai, MD | Anong Amaritakomol, RN |  |  |
|  | Juntima Euathrongchit, MD |  |  |  |
|  | Yutthaphan Wannasopha, MD |  |  |  |
|  | Sukit Yamwong, MD | Pachara Panpunuan, RN | Bangkok | Ramathibodi Hospital (1) |
|  | Piyamitr Sritara, MD |  |  |  |
|  | Suthara Aramcharoen, MD |  |  |  |
|  | Krissada Meemuk, MD |  |  |  |
| *Malaysia (2) |  |  |  |  |
| Country Leader |  |  |  |  |
| Harvey Douglas White, MD |  |  |  |  |
| Country Coordinator |  |  |  |  |
| Caroline Alsweiler |  |  |  |  |
|  | Ahmad Khairuddin, MD | Noor Syamira Mokhtar, RN | Kuala Lumpur | Institut Jantung Negara (2) |
|  | Hafidz Abd Hadi, MD | Nor Asiah Basri, RN |  |  |
|  | Shaiful Azmi Yahaya, MD | Irni Yusnida, RN |  |  |
|  |  | Humayrah Hashim |  |  |
|  |  |  |  |  |
| ** Countries participated in Economics Quality of Life (EQoL) Questionnaires* | | |  |  |
| ***This site received one participant in transfer that was randomized at another site* | | |  |  |

# Appendix II: ISCHEMIA Committee, CCC, Trial-Related Personnel

| **Past and Current Committee Members** |
| --- |
| **Leadership Committee** |
| Judith S. Hochman (Chair) |
| David J. Maron (Co-Chair) |
| William Boden (Co-Principal Investigator) |
| Robert Harrington (Co-Principal Investigator) |
| Gregg W. Stone (Co-Principal Investigator) |
| David Williams (Co-Principal Investigator) |
|  |
| **Executive Committee** |
| Judith S. Hochman (Chair) |
| David J. Maron (Co-Chair) |
| Karen P. Alexander |
| Sripal Bangalore |
| Jeffrey Berger |
| William Boden |
| Robert Harrington |
| Daniel Mark |
| Sean M. O'Brien |
| Harmony R. Reynolds |
| Yves Rosenberg |
| Leslee J. Shaw |
| John Spertus |
| Gregg W. Stone |
|  |
| **Steering Committee** |
| Judith S. Hochman (Chair) |
| David J. Maron (Co-Chair) |
| *Members of Executive Committee* |
| Christie Ballantyne*** |
| Daniel Berman |
| Rafael Beyar*** |
| Balram Bhargava |
| Chris Buller*** |
| Antonio (Tony) Carvalho** |
| Bernard R. Chaitman |
| Rafael Diaz*** |
| Rolf Doerr |
| Vladimir Dzavik |
| Shaun Goodman |
| Gilbert Gosselin |
| Rory Hachamovitch*** |
| Christian Hamm*** |
| Claes Held |
| Malte Helm*** |
| Kurt Huber*** |
| Lixin Jiang |
| Matyas Keltai |
| Shun Kohsaka |
| Irene Lang*** |
| Renato Lopes |
| Jose Lopez-Sendon |
| Aldo Maggioni |
| John Mancini |
| C. Noel Bairey Merz |
| James Min |
| Eric Peterson*** |
| Michael H. Picard |
| Witold Ruzyllo |
| Joseph Selvanayagam |
| Roxy Senior |
| Tali Sharir |
| Gabriel Steg |
| Hanna Szwed |
| Frans Van de Werf*** |
| William Weintraub |
| Harvey White |
| David Williams |
|  |
| **Optimal Medical Therapy Committee** |
| William Boden (Co-Chair) |
| David J. Maron (Co-Chair) |
| Christie Ballantyne |
| Sripal Bangalore |
| Karen Calfas **** |
| Bernard R. Chaitman |
| Mary Ann Champagne |
| Michael Davidson |
| Jerome Fleg |
| Peter A. McCullough |
| Jonathan Newman |
| Peter Stone |
|  |
| **Optimal Revascularization Therapy Planning Committee** |
| Gregg W. Stone (Chair) |
| **Subcommittee: CABG** |
| Philippe Menasche (Co-Chair) |
| Sripal Bangalore |
| Michael Davidson**** |
| Stephen Fremes |
| Robert Guyton |
| Michael Mack |
| Fred Mohr |
| Anupama Rao |
| Joe Sabik |
| Oz Shapira |
| David Taggart |
| James Tatoulis |
|  |
| **Subcommittee: PCI** |
| David Williams (Co-Chair) |
| Sripal Bangalore |
| Jim Blankenship |
| Sorin Brener |
| Chris Buller |
| Antonio Colombo |
| Bernard de Bruyne |
| Philippe Généreux |
| Robert Harrington |
| Dean Kereiakes |
| Thierry Lefevre |
| Jeffrey Moses |
|  |
| **Clinical Events** |
| **Endpoint Definition Panel** |
| Bernard R. Chaitman (Chair) |
| Karen P. Alexander |
| Judith S. Hochman |
| Ken Mahaffey |
| David J. Maron |
| Gregg W. Stone |
| Harvey White |
| **Clinical Event Review Committee** |
| Bernard R. Chaitman (Chair) |
| Salvador Cruz-Flores |
| Nicholas Danchin |
| Eli Feen |
| Mario J. Garcia |
| Paul Hauptman |
| Abhay A. Laddu |
| Eugene Passamani |
| Ileana L. Pina |
| Maarten Simoons |
| Hicham Skali |
| Kristian Thygesen |
| David Waters |
| **CEC Administrative Group** |
| Karen P. Alexander |
| Patricia Endsley*** |
| Gerard Esposito |
| Jeffrey Kanters |
| John Pownall |
| Dimitrios Stournaras |
|  |
| **ISCHEMIA Imaging Committee** |
| Leslee J. Shaw (Chair) |
| Daniel Berman |
| Matthias Friedrich |
| Rory Hachamovitch |
| Raymond Kwong |
| John Mancini |
| James Min |
| Dana Oliver |
| Michael H. Picard |
| Harmony R. Reynolds |
|  |
| **Biostatistics Planning Committee** |
| Frank Harrell (Chair) |
| Jeffrey Blume |
| Kerry Lee |
| Sean M. O'Brien |
|  |
| **BioRepository Committee** |
| Jeffrey Berger (Chair) |
| Claes Held |
| Iftikhar Kullo |
| Bruce McManus |
| Kristin Newby |
|  |
| **EQOL Committee** |
| Daniel Mark (Co-Chair) |
| John Spertus (Co-Chair) |
| David Cohen |
| William Weintraub |
|  |
| **Recruitment for Women & Minorities** |
| C. Noel Bairey Merz (Chair) |
| Raffaele Bugiardini |
| Jelena Celutkiene |
| Jorge Escobedo |
| Angela Hoye |
| Radmila Lyubarova |
| Deirdre Mattina |
| Jesus Peteiro |
| Harmony R. Reynolds |
| Paola Smanio |
|  |
| **Publications** |
| David J. Maron (Chair) |
| Karen P. Alexander |
| Sripal Bangalore |
| Jeffrey Berger |
| William Boden |
| Robert Harrington |
| Judith S. Hochman |
| Sean M. O'Brien |
| Harmony R. Reynolds |
| Yves Rosenberg |
| Gregg W. Stone |
| **Publication Subcommittees** |
| **Economics** |
| Daniel Mark (Chair) |
| John Spertus |
| **QOL** |
| John Spertus (Chair) |
| Daniel Mark |
| **Stress Testing** |
| Leslee J. Shaw (Chair) |
| Dan Berman |
| Bernard R. Chaitman |
| Jerome Fleg |
| Raymond Kwong |
| Michael H. Picard |
| Harmony R. Reynolds |
| Roxy Senior |
| **CCTA** |
| James Min (Chair) |
| Jonathan Leipsic |
| John Mancini |
| **Angiography/Optimal Revascularization Therapy** |
| Gregg W. Stone (Chair) |
| Ziad Ali (Co-chair) |
| Sripal Bangalore |
| David Williams |
| (Philippe Genereux, former Chair, Angiography Subcommittee)* |
| **Optimal Medical Therapy** |
| William Boden (Co-Chair) |
| David J. Maron (Co-Chair) |
| Jerome Fleg |
| Jonathan Newman |
| **Biorepository** |
| Jeffrey Berger (Chair) |
| **CEC** |
| Bernard R. Chaitman (Chair) |
| Karen P. Alexander |
| **CKD** |
| Sripal Bangalore (Chair) |
| Karen P. Alexander |
| Jerome Fleg |
| Judith S. Hochman |
| David J. Maron |
| Roy Mathew |
| Sean M. O'Brien |
| Harmony R. Reynolds |
| Mandeep Sidhu |
| **CIAO** |
| Harmony R. Reynolds (Chair) |
|  |
|  |
| **DSMB Members** |
| Lawrence Friedman (Chair) |
| Jeffrey Anderson |
| Jessica Berg *** |
| David DeMets |
| C. Michael Gibson |
| Gervasio Lamas |
| Nicole Deming |
| Jonathan Himmelfarb |
| Pamela Ouyang |
| Pamela Woodard |
|  |
| **Independent Statistical Analysis Center for DSMB Reporting** |
| Frank Harrell |
| Samuel Nwosu |
|  |
| **NHLBI Program Staff** |
| **Project Office** |
| Yves Rosenberg (Project Officer) |
| Jerome Fleg |
| Ruth Kirby |
| **Statisticians** |
| Neal Jeffries |
|  |
| **ISCHEMIA Clinical Coordinating Center (CCC)** |
| **Study Leadership** |
| Judith S. Hochman (Study Chair, Director of CCC) |
| David J. Maron (Study Co-Chair, Co-Director of CCC, US Country Leader) |
| **CCC Faculty** |
| Sripal Bangalore (Optimal Revascularization Therapy CCC Director, Regional Leader) |
| Jeffrey Berger (Director of the Biorepository, Regional Leader) |
| William Boden (US-VA Regional Leader) |
| Jonathan Newman (Optimal Medical Therapy CCC Director, Regional Leader) |
| Harmony R. Reynolds (Associate Director of CCC, CCC Imaging Lead, Regional Leader) |
| Mandeep Sidhu (US-VA Regional Co-Leader) |
| ***Program Directors*** |
| Jean E. Denaro**** |
| Stephanie Mavromichalis |
| ***Project Managers*** |
| Kevin Chan |
| Gia Cobb* |
| Aira Contreras |
| Diana Cukali* |
| Stephanie Ferket*** |
| Andre Gabriel*** |
| Antonietta Hansen* |
| Arline Roberts |
| ***Clinical Research Associates*** |
| Michelle Chang |
| Sharder Islam* |
| Graceanne Wayser* |
| Solomon Yakubov*** |
| Michelle Yee |
| ***Clinical Trial Assistants*** |
| Caroline Callison |
| Isabelle Hogan |
| Albertina Qelaj* |
| Charlotte Pirro* |
| Kerrie Van Loo |
| Brianna Wisniewski* |
| ***Grants and Finance Administration*** |
| Margaret Gilsenan (Grants Manager) |
| Bevin Lang |
| Samaa Mohamed |
| ***Publications Team*** |
| Shari Esquenazi-Karonika (Publications Manager) |
| Patenne Mathews  Anna Naumova  Jihyun Lyo* |
| ***Data Analyst*** |
| Vincent Setang* |
| Mark Xavier* |
|  |
| **Statistical and Data Coordinating Center (SDCC)** |
| Sean M. O’Brien (Principal Investigator) |
| Karen P. Alexander (Co-Principal Investigator) |
|  |
| **Economics and Quality of Life Coordinating Center (EQOL CC)** |
| *Duke Clinical Research Institute, Durham, NC* |
| Daniel B. Mark (Principal Investigator) |
| Kevin Anstrom |
| Khaula Baloch |
| Janet Blount |
| Patricia Cowper |
| Linda Davidson-Ray |
| Laura Drew |
| Tina Harding |
| J David Knight |
| Diane Minshall Liu |
| Betsy O’Neal |
| Thomas Redick |
|  |
| *Saint Luke’s Mid America Heart Institute, Kansas City, MO* |
| John Spertus (Principal Investigator) |
| Philip Jones |
| Karen Nugent |
| Grace Jingyan Wang |
|  |
| **ISCHEMIA Imaging Coordinating Center (ICC)** |
| Leslee J. Shaw (Principal Investigator) |
| Lawrence Phillips |
| Abhinav Goyal |
| Holly Hetrick |
| Dana Oliver |
| *Nuclear Core Lab* |
| Daniel Berman (Director) |
| Sean W. Hayes (Co-Director) |
| John D. Friedman |
| R. James Gerlach |
| Mark Hyun |
| Romalisa Miranda-Peats |
| Piotr Slomka |
| Louise Thomson |
| *CMR Core Lab* |
| Raymond Y. Kwong (Director) |
| Matthias Friedrich (Director)*** |
| Francois Pierre Mongeon (Co-Director) |
| Steven Michael |
| *Echo Core Lab* |
| Michael H. Picard (Director) |
| Judy Hung |
| Marielle Scherrer-Crosbie |
| Xin Zeng |
|  |
| **ECG/ETT CoreLab** |
| Bernard R. Chaitman (Director) |
| Jane Eckstein |
| Bandula Guruge |
| Mary Streif |
|  |
| **Angiographic Core Lab** |
| Ziad Ali (Director) |
| Philippe Genereux (Director)*** |
| Maria A. Alfonso |
| Maria P. Corral |
| Javier J. Garcia |
| Jennifer Horst |
| Ivana Jankovic |
| Maayan Konigstein |
| Mitchel B. Lustre* |
| Yolayfi Peralta |
| Raquel Sanchez |
|  |
| **CCTA Core Lab** |
| James Min (Director) |
| Reza Arsanjani |
| Matthew Budoff |
| Kimberly Elmore |
| Millie Gomez |
| Cameron Hague |
| Niree Hindoyan |
| Jonathan Leipsic |
| GB John Mancini |
| Rine Nakanishi |
| M. Barbara Srichai-Parsia |
| Eunice Yeoh |
| Tricia Youn |
|  |
| **Academic Research Organizations (AROs)** |
| *Associazione Nazionale Medici Cardiologi Ospedalieri (ANMCO) -Italy & Switzerland* |
| Aldo P. Maggioni (Country Leader) |
| Francesca Bianchini |
| Martina Ceseri |
| Andrea Lorimer |
| Marco Magnoni |
| Francesco Orso |
| Laura Sarti |
| Martinia Tricoli* |
| *Brazilian Clinical Research Institute (BCRI) - Brazil* |
| Antonio Carvalho (Country Leader)** |
| Renato Lopes (Country Leader) |
| Lilian Mazza Barbosa |
| Tauane Bello Duarte |
| Tamara Colaiácovo Soares |
| Julia de Aveiro Morata |
| Pedro Carvalho |
| Natalia de Carvalho Maffei |
| Flávia Egydio* |
| Anelise Kawakami* |
| Janaina Oliveira* |
| Elissa Restelli Piloto* |
| Jaqueline Pozzibon*** |
| *Canadian Heart Research Centre (CHRC) - Canada* |
| Shaun Goodman (Country Leader) |
| Diane Camara |
| Neamat Mowafy |
| Caroline Spindler |
| *China Oxford Centre for International Health Research - China* |
| Lixin Jiang (Country Leader) |
| Hao Dai |
| Fang Feng |
| Jia Li |
| Li Li* |
| Jiamin Liu |
| Qiulan Xie |
| Haibo Zhang |
| Jianxin Zhang |
| Lihua Zhang |
| Liping Zhang |
| Ning Zhang |
| Hui Zhong |
| *Estudios Clínicos Latino America (ECLA) - Argentina* |
| Rafael Diaz*** |
| Claudia Escobar |
| Maria Eugenia Martin* |
| Andrea Pascual* |
| *Foundation for Biomedical Research of La Paz University Hospital (FIBHULP) - Spain* |
| José Lopez-Sendon (Country Leader) |
| Paloma Moraga |
| Victoria Hernandez |
| Almudena Castro |
| Maria Posada* |
| Sara Fernandez |
| José Luis Narro Villanueva |
| Rafael Selgas |
| *French Alliance for Cardiovascular Trials (FACT) - France* |
| Gabriel Steg (Country Leader) |
| Helene Abergel |
| Jean Michel Juliard |
| *Green Lane Coordinating Centre Ltd. (GLCC) -Malaysia, New Zealand, Singapore, Taiwan, Thailand* |
| Harvey White (Country Leader) |
| Caroline Alsweiler |
| *KU Leuven Research & Development - Belgium** |
| Frans Van de Werf (Country Leader) |
| Kathleen Claes |
| Kaatje Goetschalckx |
| Ann Luyten |
| Valerie Robesyn |
| *South Australian Health and Medical Research Institute Ltd (SAHMRI) - Australia* |
| Joseph B. Selvanayagam (Country Leader) |
| Deirdre Murphy |
|  |
| **Contract Research Organizations (CROs) for ISCHEMIA Trial** |
| *FOCUS Clinical Research Center d.o.o. Belgrade - Serbia* |
| Nevena Garcevic |
| Jelena Stojkovic |
| *iProcess Global Research Inc. - India* |
| Asker Ahmed |
| Richa Bhatt |
| Nitika Chadha* |
| Vijay Kumar* |
| Sadath Lubna*** |
| Pushpa Naik |
| Shruti Pandey* |
| Karthik Ramasamy* |
| Mohammed Saleem |
| Pratiksha Sharma |
| Hemalata Siddaram* |
|  |
| **past members / past organizations*  ***deceased* |
